# Supplementary material for: Targeted proteomics as a tool to detect SARS-CoV-2 proteins in clinical specimens
Source: PLoS One. 2021 Nov 11;16(11):e0259165. doi: 10.1371/journal.pone.0259165 (PMC8584957; doi:10.1371/journal.pone.0259165)
Supplement: S1 Fig — MS/MS spectra of tryptic peptides A) GFYAEGSR, B) ADETQALPQR and C) EITVATSR. Data visualization in PDV proteomics viewer (pdv.zhang-lab.org). (PPTX) [file pone.0259165.s001.pptx]

## Slide 1
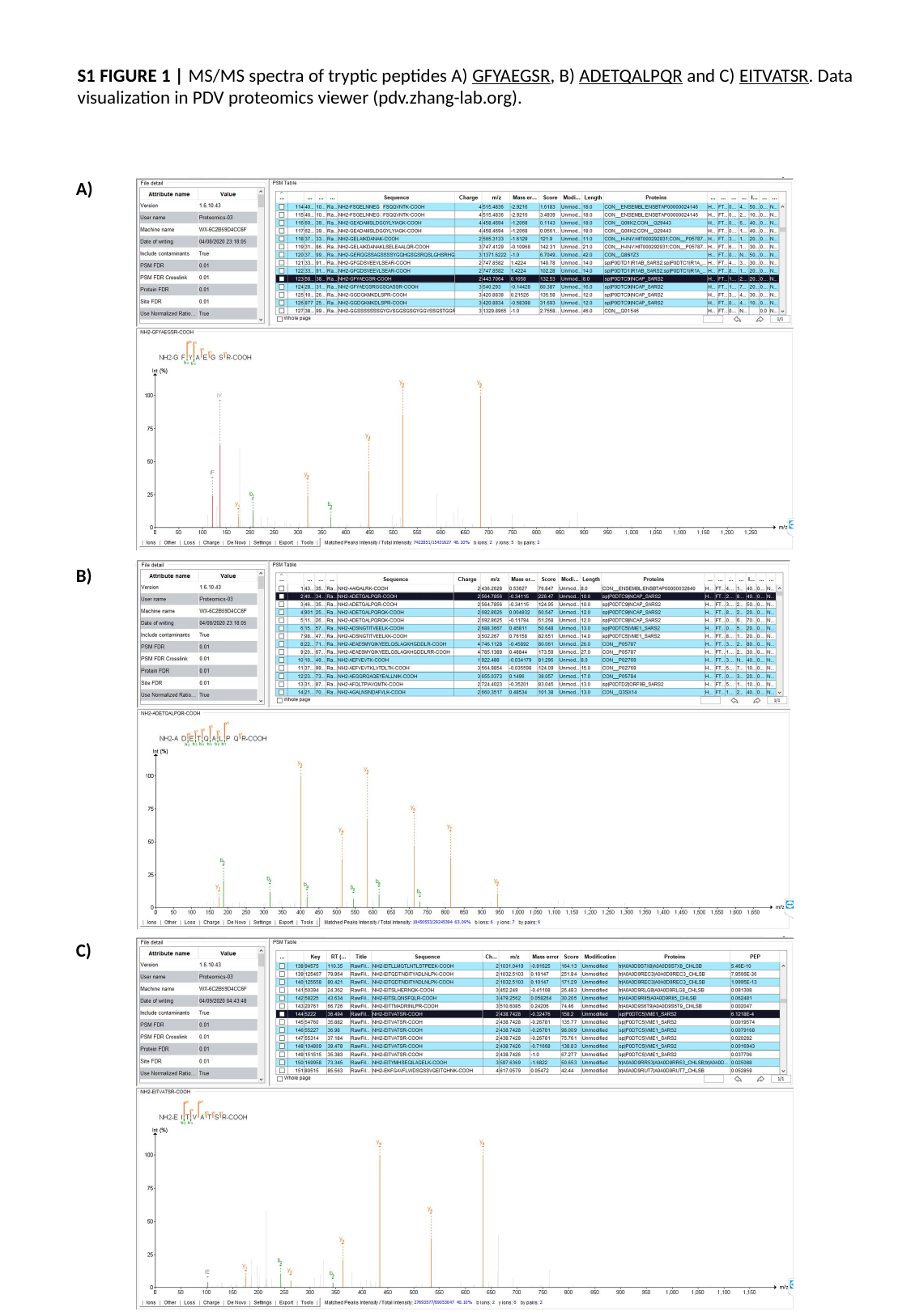

S1 FIGURE 1 | MS/MS spectra of tryptic peptides A) GFYAEGSR, B) ADETQALPQR and C) EITVATSR. Data visualization in PDV proteomics viewer (pdv.zhang-lab.org).
A)
B)
C)
