# Supplementary material for: Targeted proteomics as a tool to detect SARS-CoV-2 proteins in clinical specimens
Source: PLoS One. 2021 Nov 11;16(11):e0259165. doi: 10.1371/journal.pone.0259165 (PMC8584957; doi:10.1371/journal.pone.0259165)
Supplement: S2 Fig — Tryptic peptide coverage in light green of A) Nucleocapsid (NCAP_SARS2) and B) Membrane protein (VME1_SARS2). Data visualization in PD2.3. (PPTX) [file pone.0259165.s002.pptx]

## Slide 1
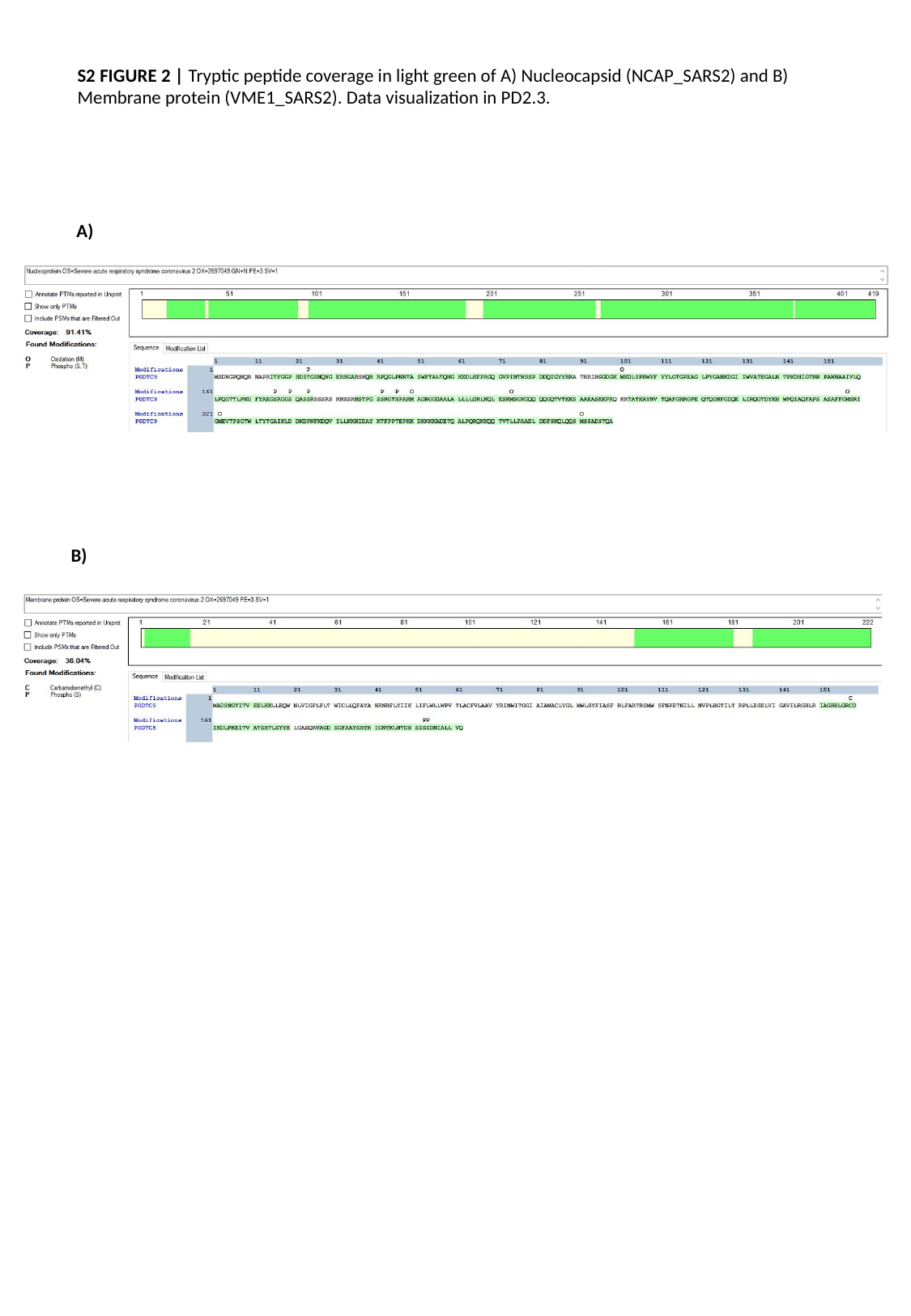

S2 FIGURE 2 | Tryptic peptide coverage in light green of A) Nucleocapsid (NCAP_SARS2) and B) Membrane protein (VME1_SARS2). Data visualization in PD2.3.
A)
B)
