# Supplementary material for: Targeted proteomics as a tool to detect SARS-CoV-2 proteins in clinical specimens
Source: PLoS One. 2021 Nov 11;16(11):e0259165. doi: 10.1371/journal.pone.0259165 (PMC8584957; doi:10.1371/journal.pone.0259165)
Supplement: S3 Fig — Fragment ion chromatograms for each of the Top5 or Top6 fragment ions are shown in different colors in a dilution series for tryptic peptides A) ADETQALPQR (NCAP_SARS2) and B) EITVATSR (VME1_SARS2). C) Library peptide fragmentation spectra for the indicated peptides. D) Calibration bar graphs for three target peptides. (PPTX) [file pone.0259165.s003.pptx]

## Slide 1
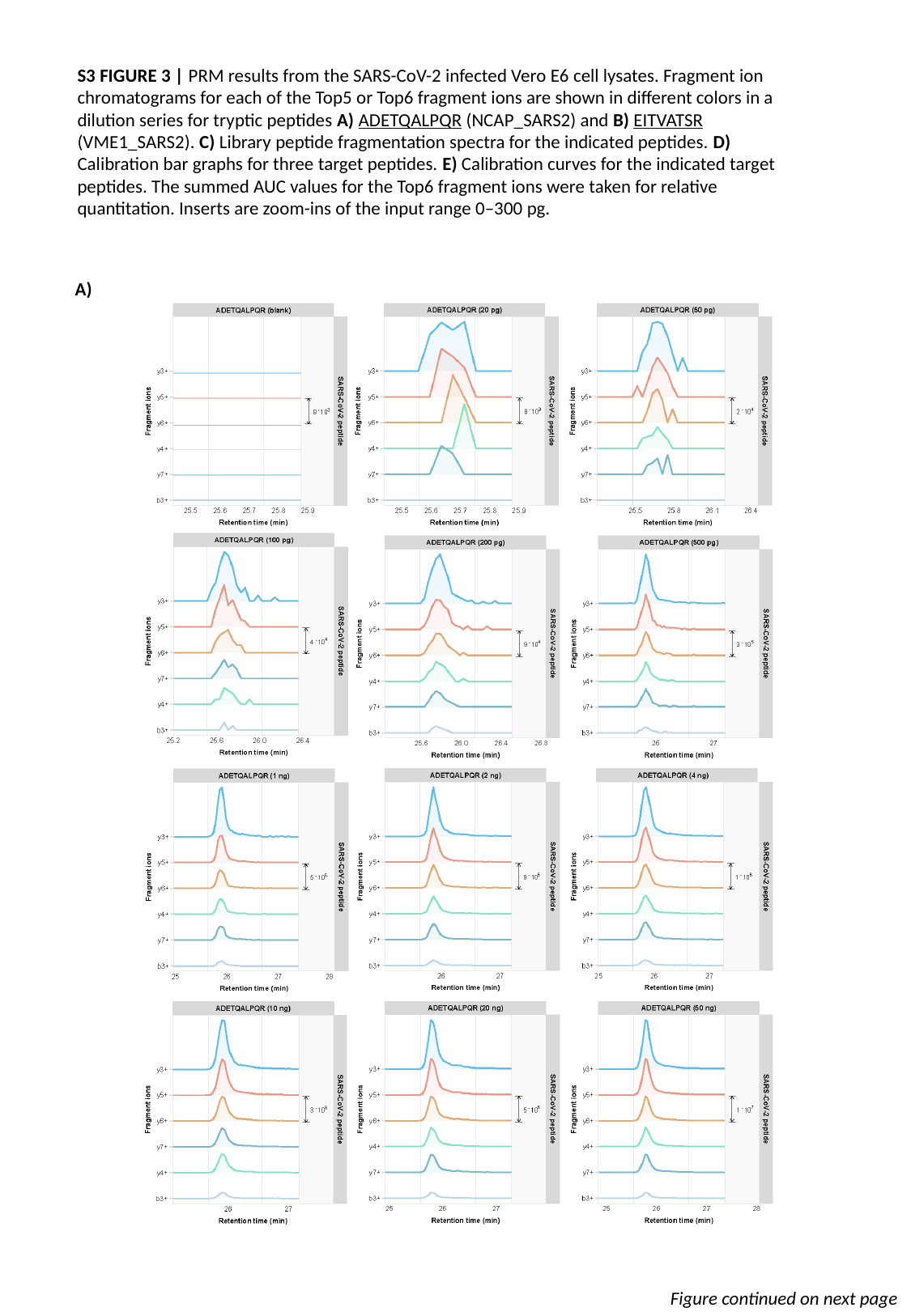

S3 FIGURE 3 | PRM results from the SARS-CoV-2 infected Vero E6 cell lysates. Fragment ion chromatograms for each of the Top5 or Top6 fragment ions are shown in different colors in a dilution series for tryptic peptides A) ADETQALPQR (NCAP_SARS2) and B) EITVATSR (VME1_SARS2). C) Library peptide fragmentation spectra for the indicated peptides. D) Calibration bar graphs for three target peptides. E) Calibration curves for the indicated target peptides. The summed AUC values for the Top6 fragment ions were taken for relative quantitation. Inserts are zoom-ins of the input range 0–300 pg.
A)
Figure continued on next page

## Slide 2
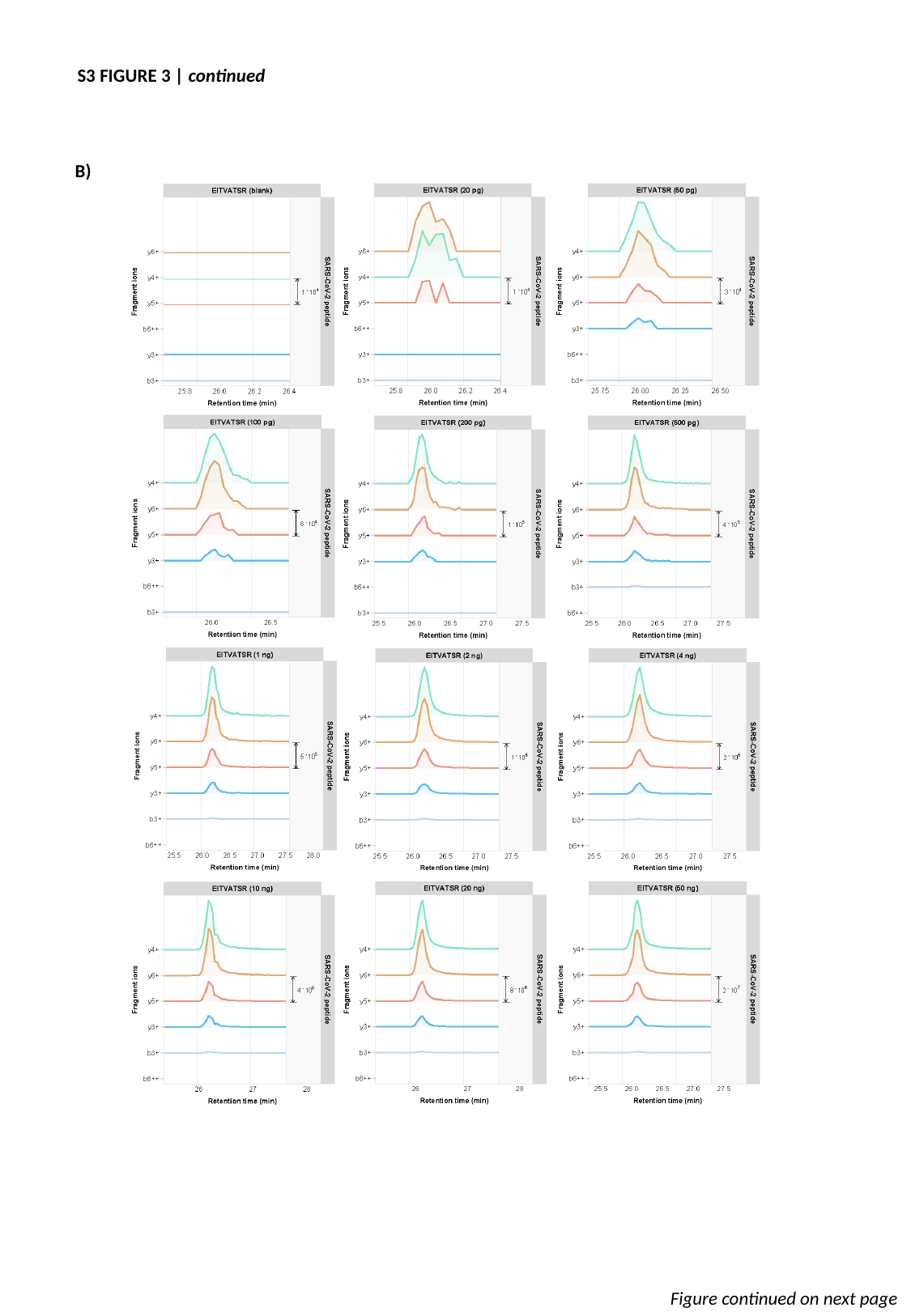

S3 FIGURE 3 | continued
B)
Figure continued on next page

## Slide 3
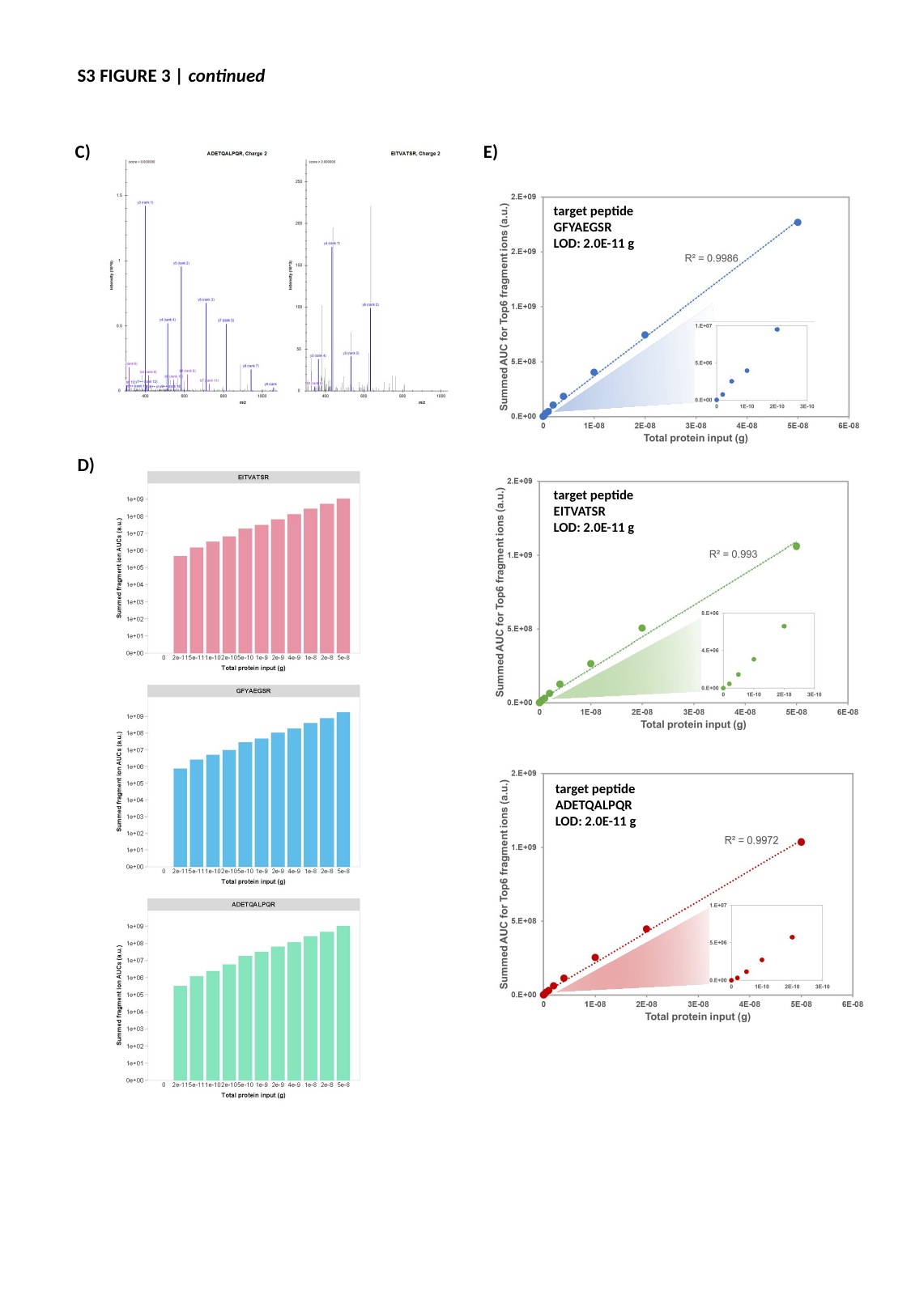

S3 FIGURE 3 | continued
C)
E)
target peptide
GFYAEGSR
LOD: 2.0E-11 g
D)
target peptide
EITVATSR
LOD: 2.0E-11 g
target peptide
ADETQALPQR
LOD: 2.0E-11 g
