# Supplementary material for: Targeted proteomics as a tool to detect SARS-CoV-2 proteins in clinical specimens
Source: PLoS One. 2021 Nov 11;16(11):e0259165. doi: 10.1371/journal.pone.0259165 (PMC8584957; doi:10.1371/journal.pone.0259165)
Supplement: S4 Fig — A) PRM chromatograms of SARS-CoV-2 Nucleocapsid and VME1 tryptic peptides AYNVTQAFGR and VAGDSFAAYSR in four additional COVID-19 patient sputum specimens (#s 3–6) and one specimen from a patient infected with influenza B serving as a negative control (# 3). Chromatograms for each of the Top6 fragment ions are shown in different colors. The upper panels show the fragment ion chromatograms of the corresponding synthetic AQUA peptide AYNVTQAFG[R] (m/z 568.79) and VAGDSFAAYS[R] (m/z 605.79). S3 File contains the output in table format, including Skyline library dot product and total area fragment values. B) The corresponding Ct values for the sputum and throat swab samples from PCR assays. (PPTX) [file pone.0259165.s004.pptx]

## Slide 1
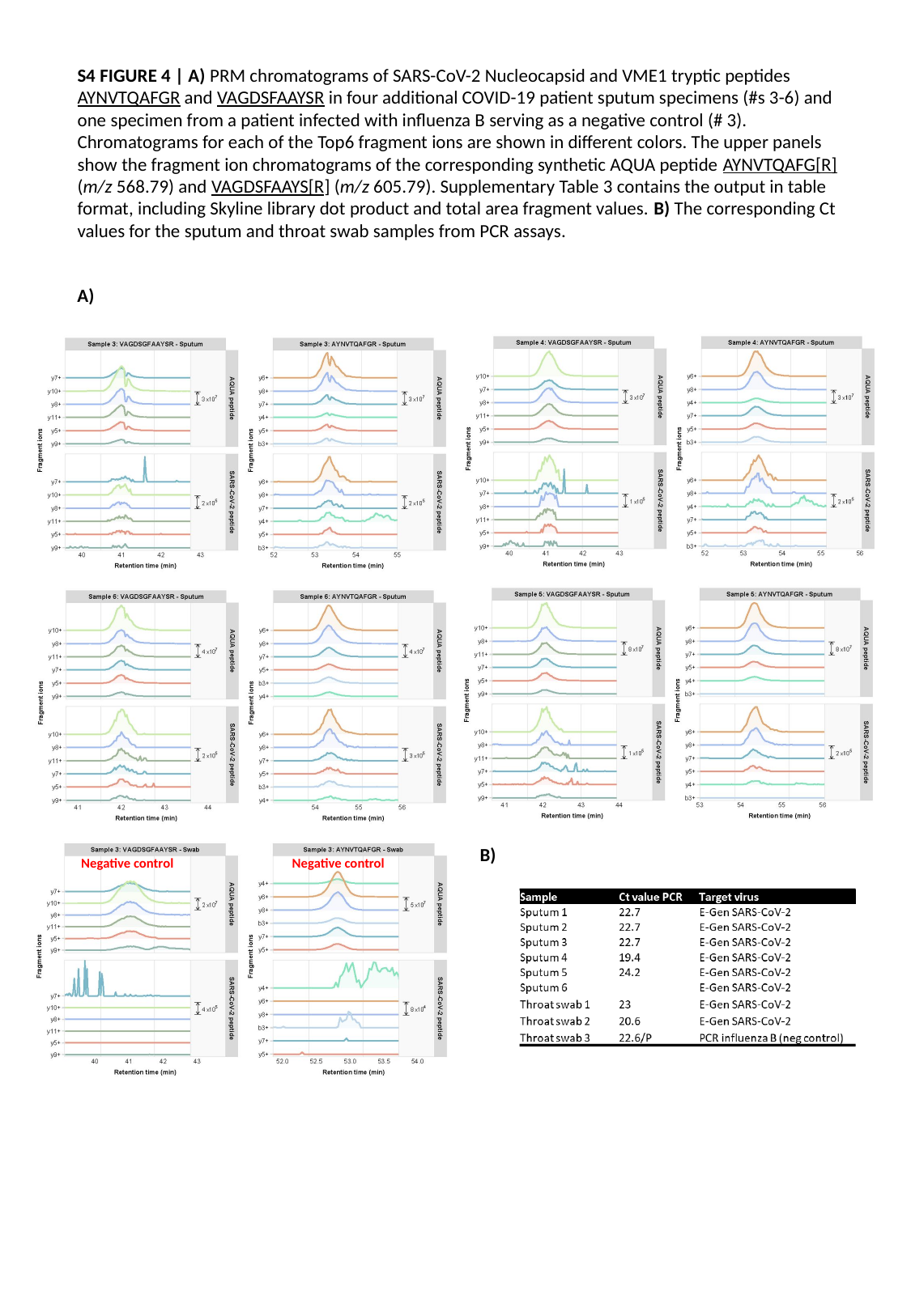

S4 FIGURE 4 | A) PRM chromatograms of SARS-CoV-2 Nucleocapsid and VME1 tryptic peptides AYNVTQAFGR and VAGDSFAAYSR in four additional COVID-19 patient sputum specimens (#s 3-6) and one specimen from a patient infected with influenza B serving as a negative control (# 3). Chromatograms for each of the Top6 fragment ions are shown in different colors. The upper panels show the fragment ion chromatograms of the corresponding synthetic AQUA peptide AYNVTQAFG[R] (m/z 568.79) and VAGDSFAAYS[R] (m/z 605.79). Supplementary Table 3 contains the output in table format, including Skyline library dot product and total area fragment values. B) The corresponding Ct values for the sputum and throat swab samples from PCR assays.
A)
B)
Negative control
Negative control
