# Supplementary material for: Targeted proteomics as a tool to detect SARS-CoV-2 proteins in clinical specimens
Source: PLoS One. 2021 Nov 11;16(11):e0259165. doi: 10.1371/journal.pone.0259165 (PMC8584957; doi:10.1371/journal.pone.0259165)
Supplement: S5 Fig — (PPTX) [file pone.0259165.s005.pptx]

## Slide 1
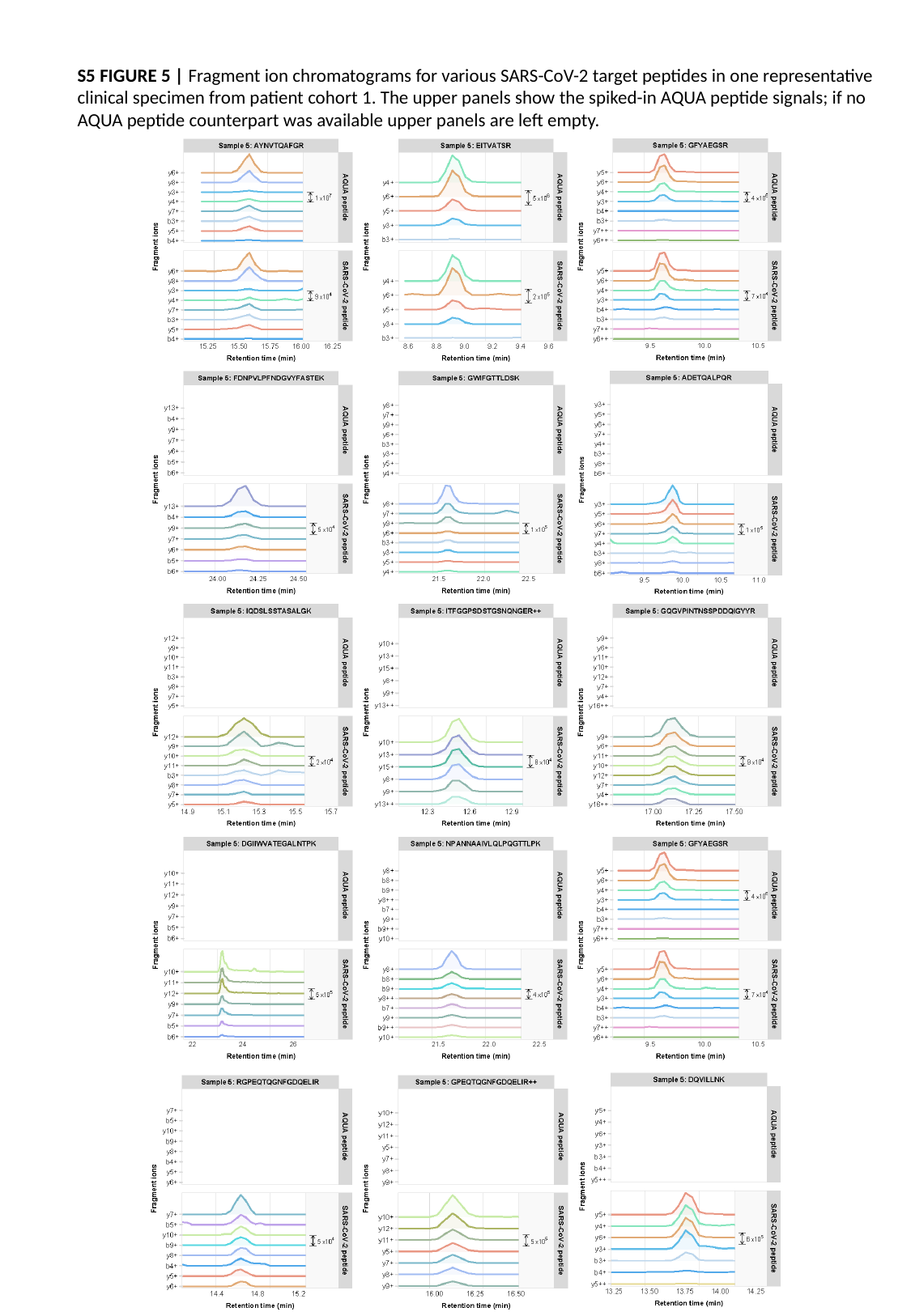

S5 FIGURE 5 | Fragment ion chromatograms for various SARS-CoV-2 target peptides in one representative clinical specimen from patient cohort 1. The upper panels show the spiked-in AQUA peptide signals; if no AQUA peptide counterpart was available upper panels are left empty.
