# Supplementary material for: Targeted proteomics as a tool to detect SARS-CoV-2 proteins in clinical specimens
Source: PLoS One. 2021 Nov 11;16(11):e0259165. doi: 10.1371/journal.pone.0259165 (PMC8584957; doi:10.1371/journal.pone.0259165)
Supplement: S6 Fig — (PPTX) [file pone.0259165.s006.pptx]

## Slide 1
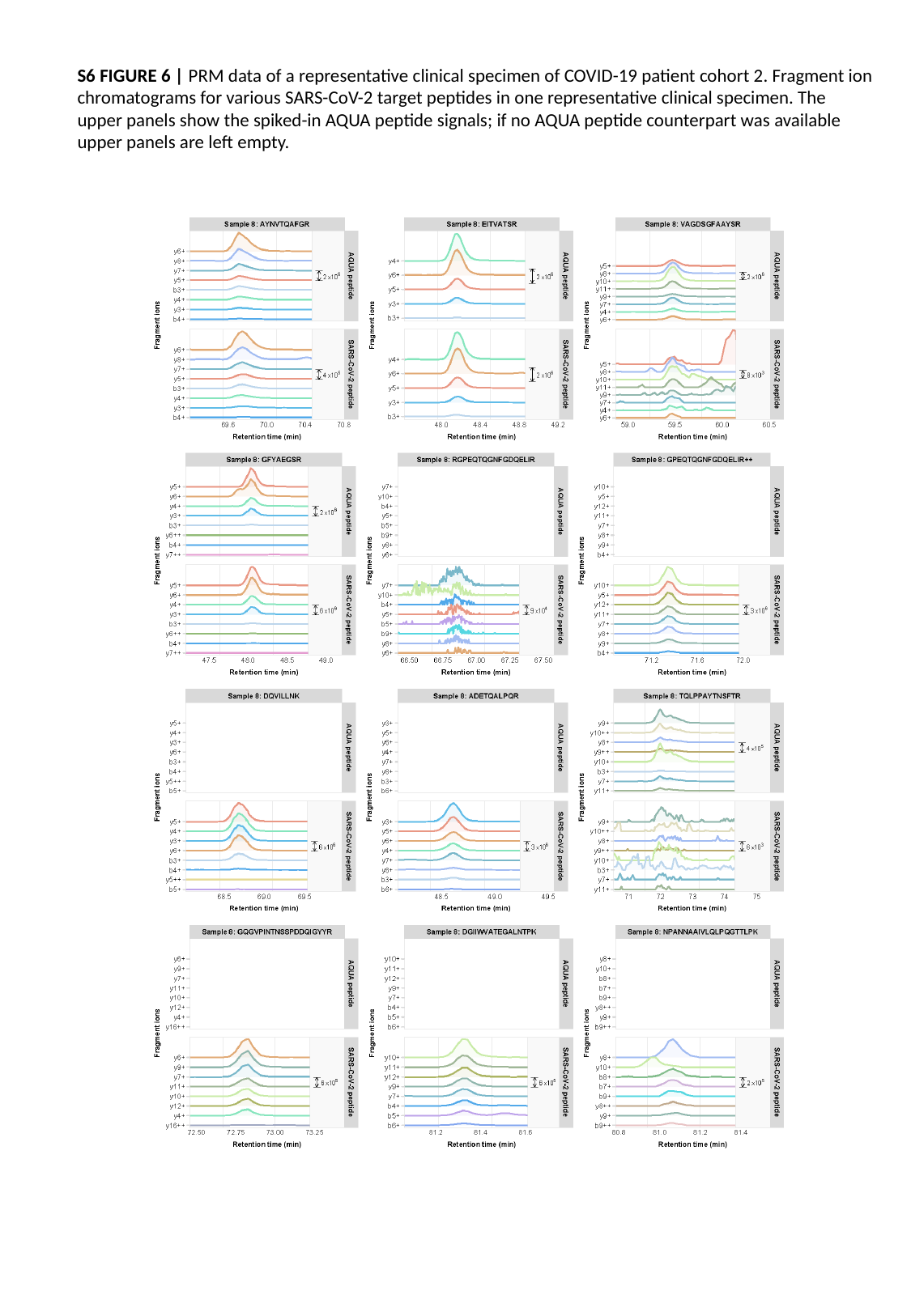

S6 FIGURE 6 | PRM data of a representative clinical specimen of COVID-19 patient cohort 2. Fragment ion chromatograms for various SARS-CoV-2 target peptides in one representative clinical specimen. The upper panels show the spiked-in AQUA peptide signals; if no AQUA peptide counterpart was available upper panels are left empty.
