# Supplementary figures and images for: Targeted proteomics as a tool to detect SARS-CoV-2 proteins in clinical specimens
Source: PLoS One. 2021 Nov 11;16(11):e0259165. doi: 10.1371/journal.pone.0259165 (PMC8584957; doi:10.1371/journal.pone.0259165)

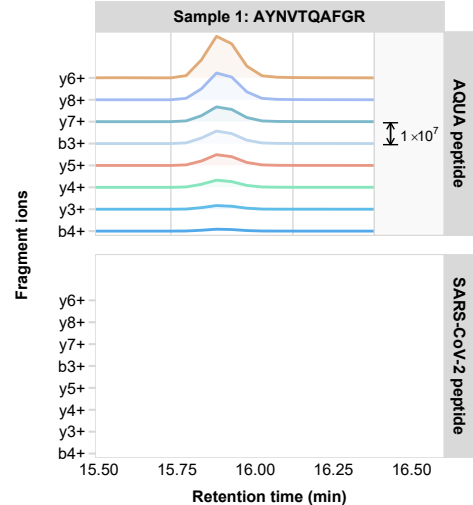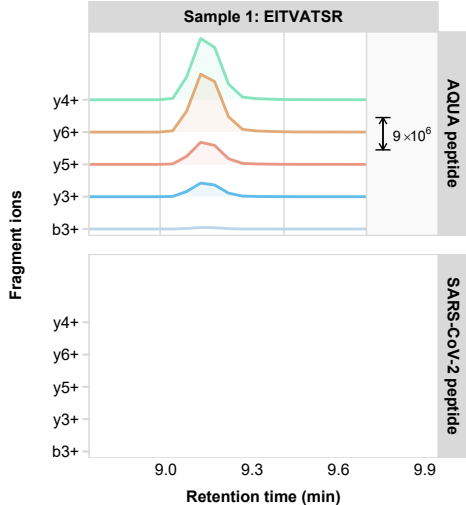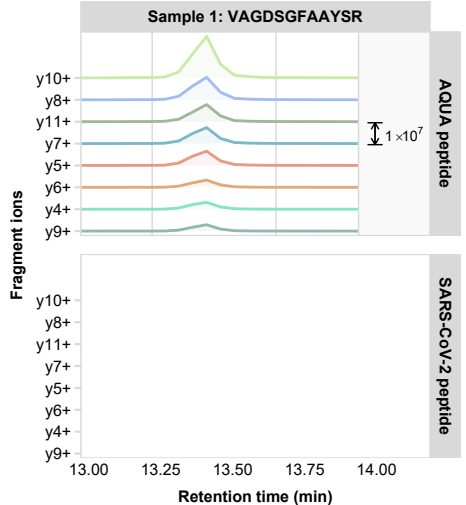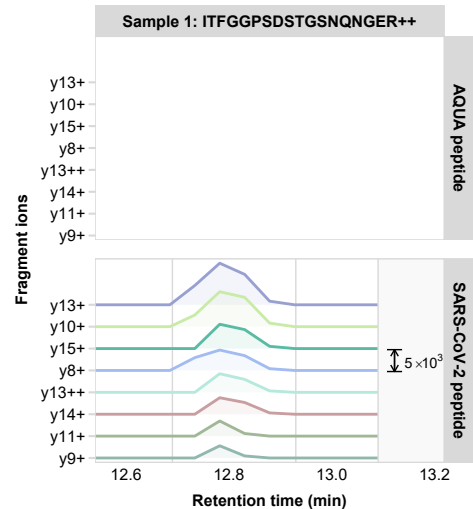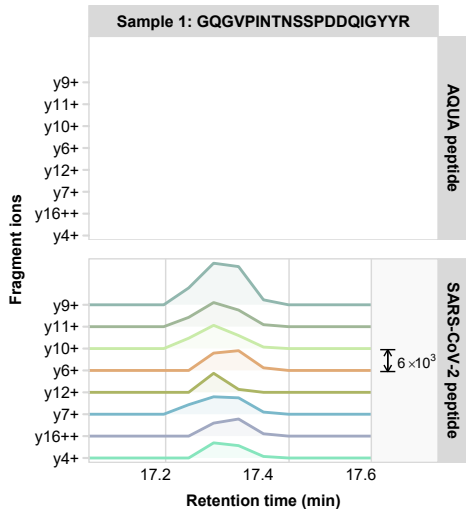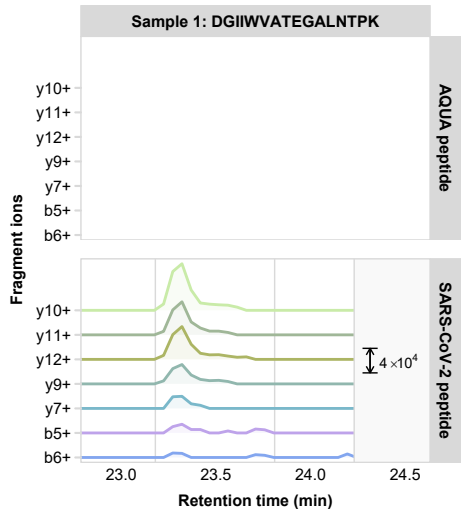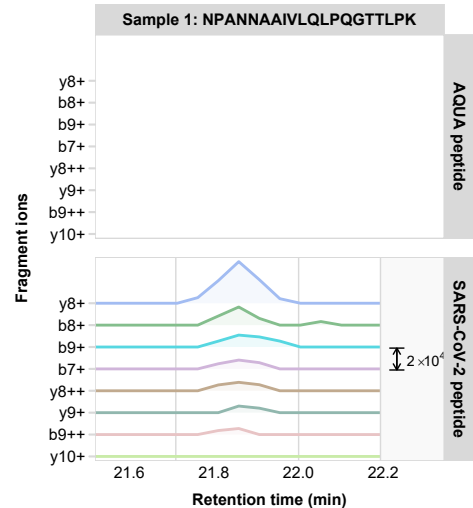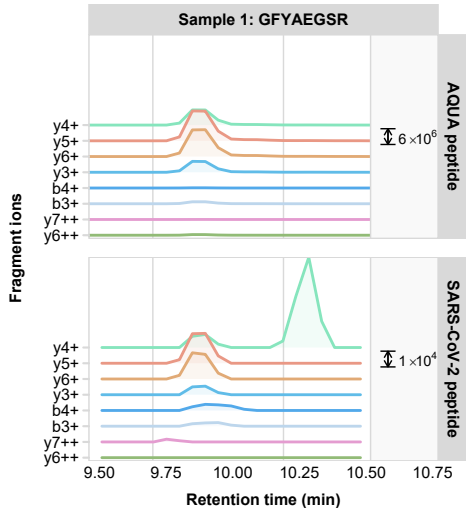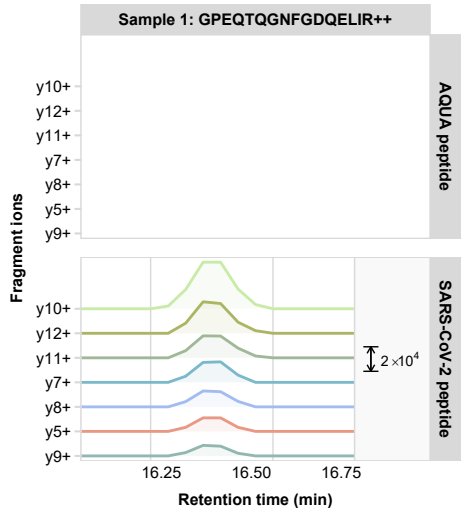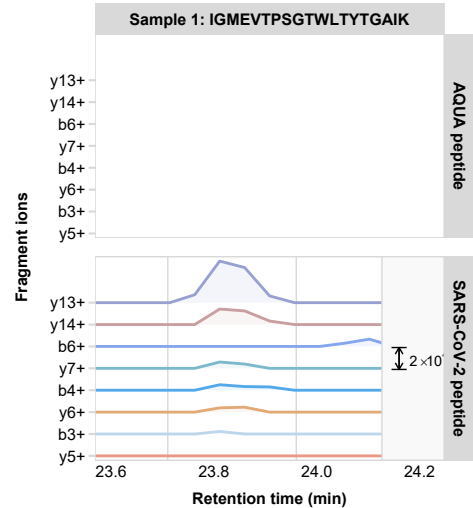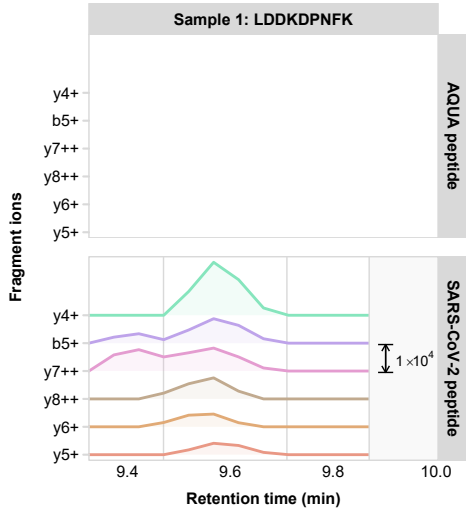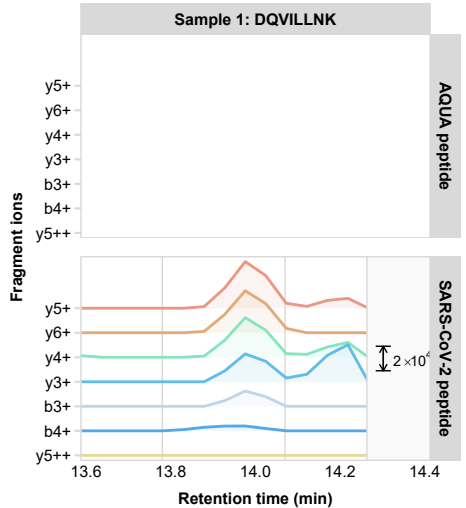

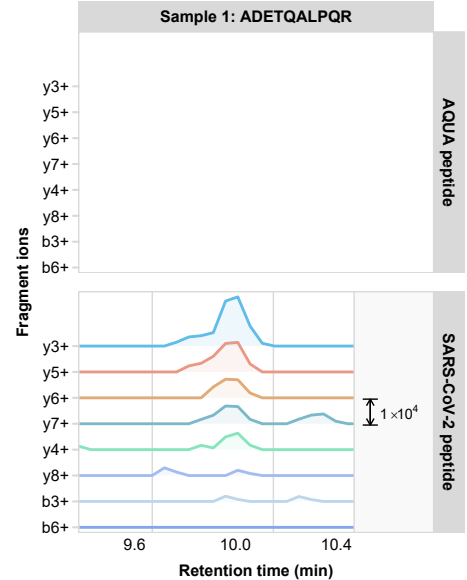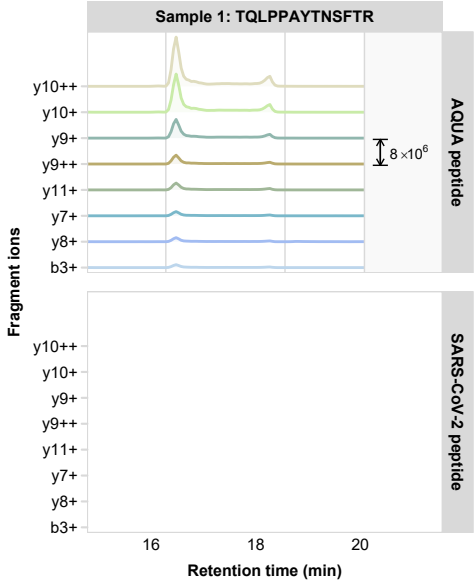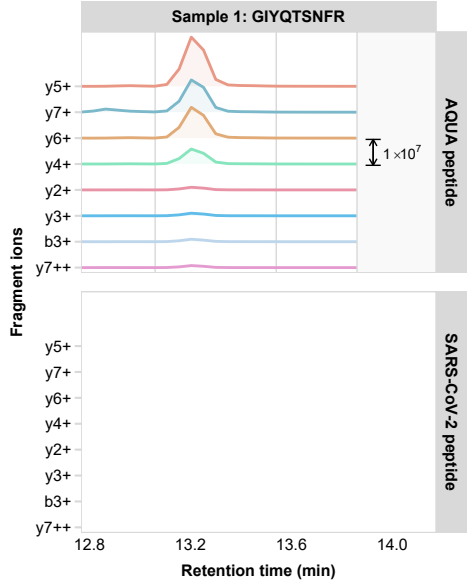

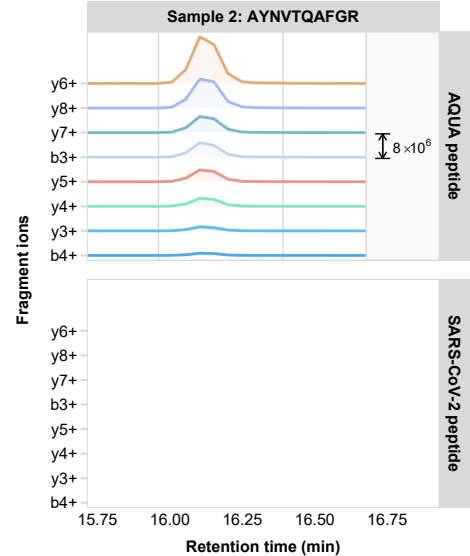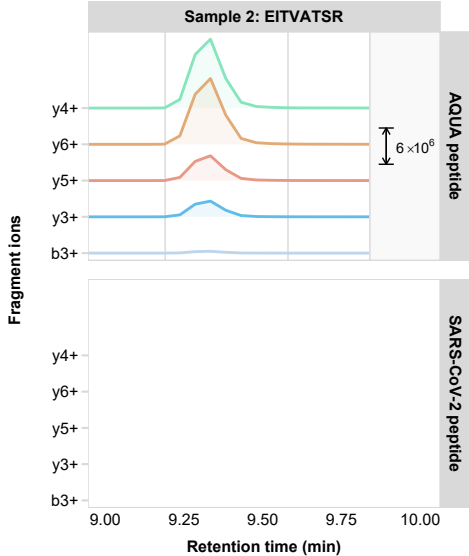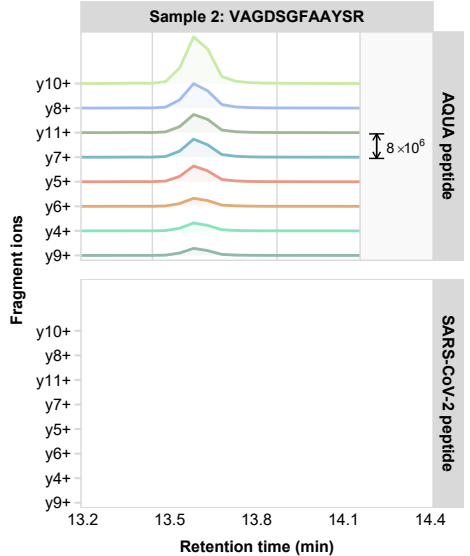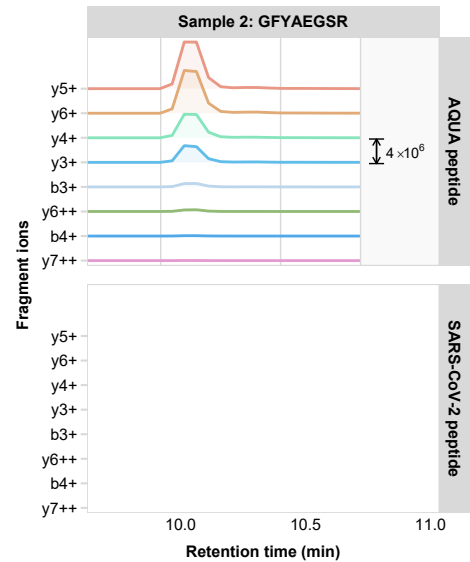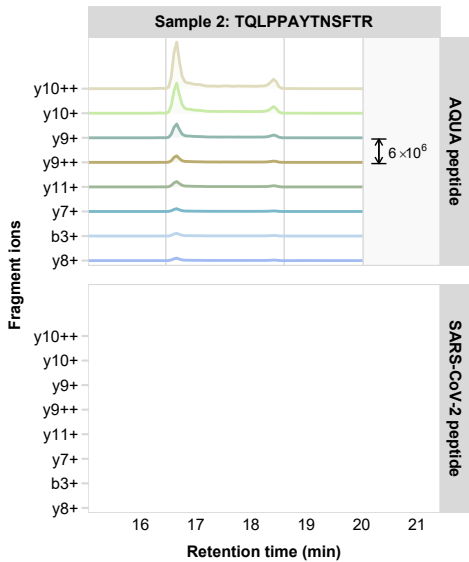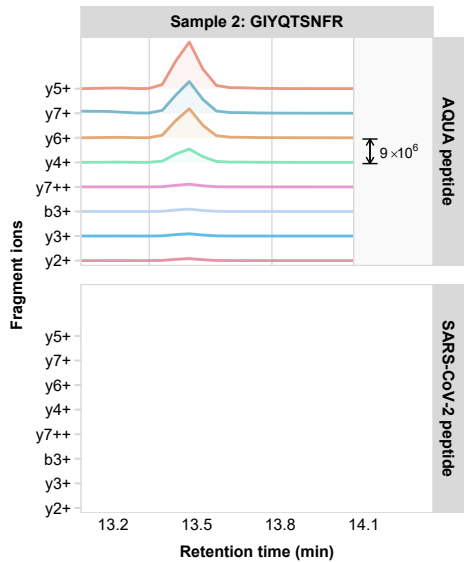

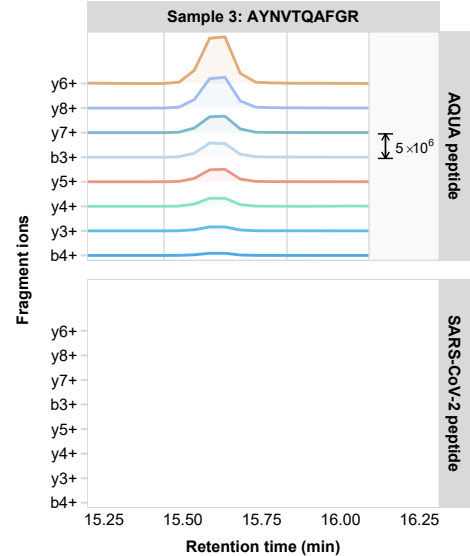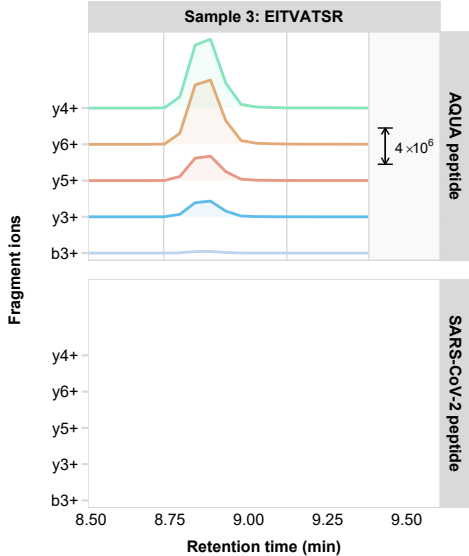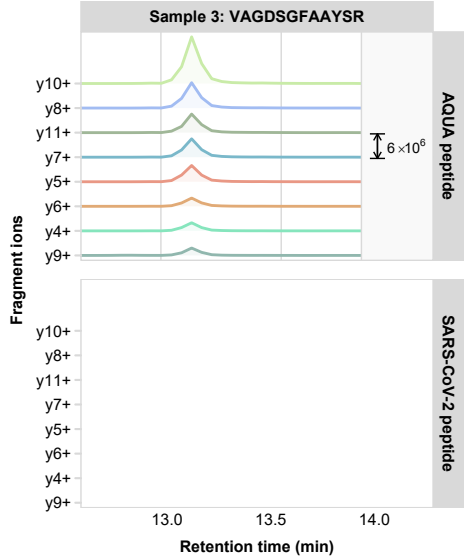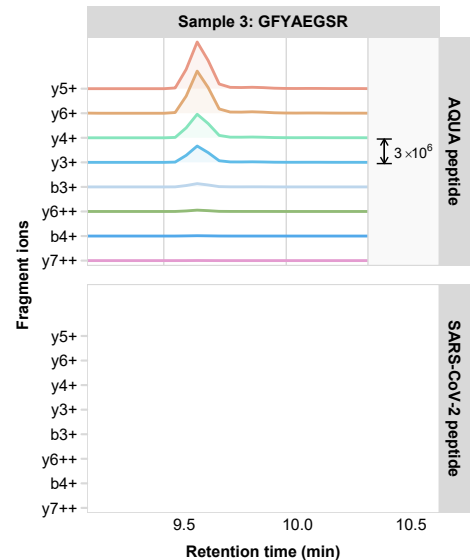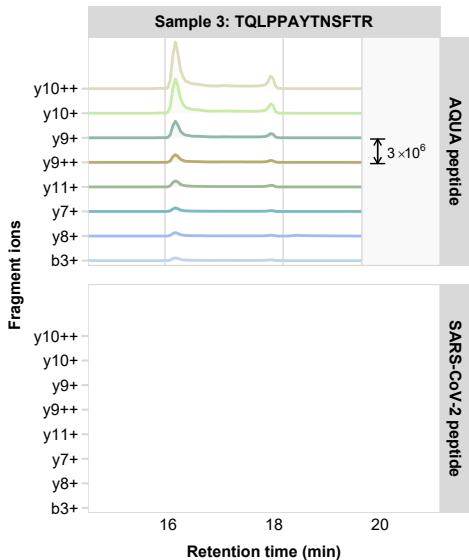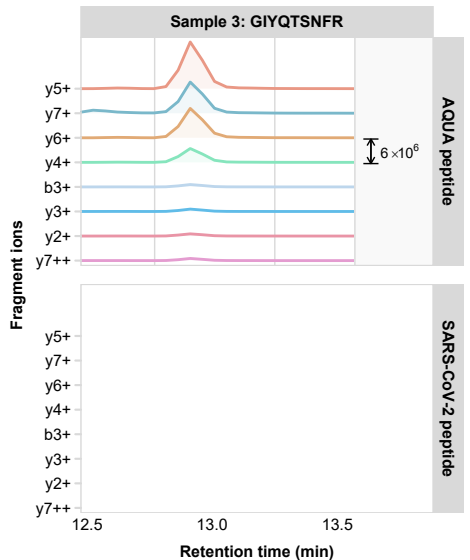

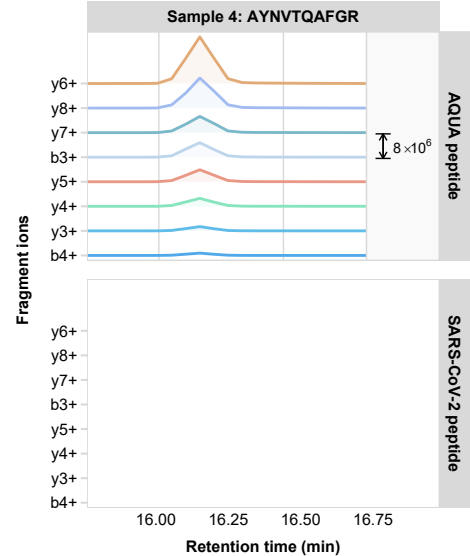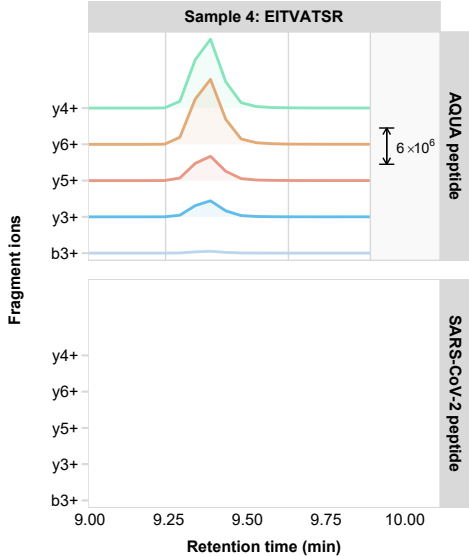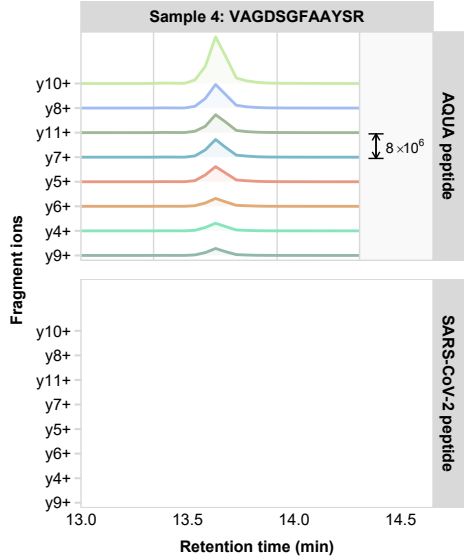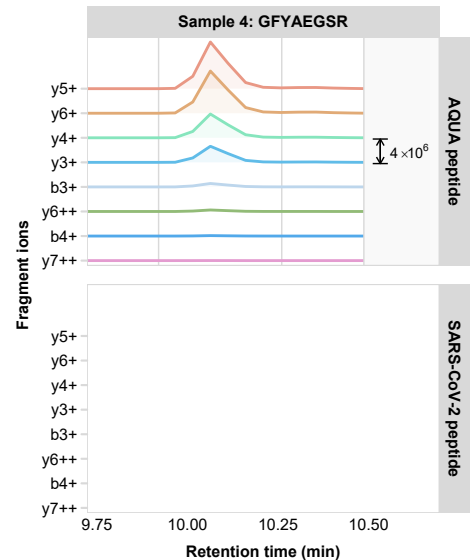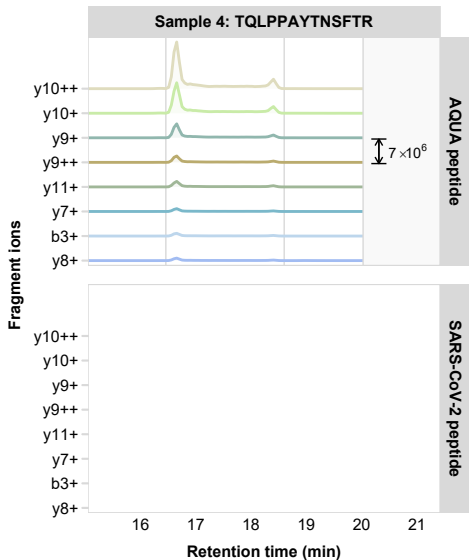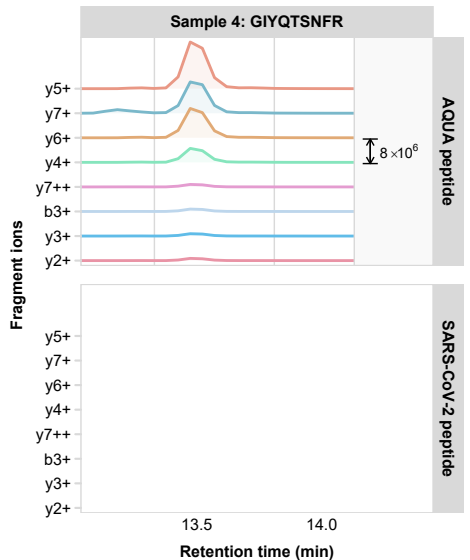

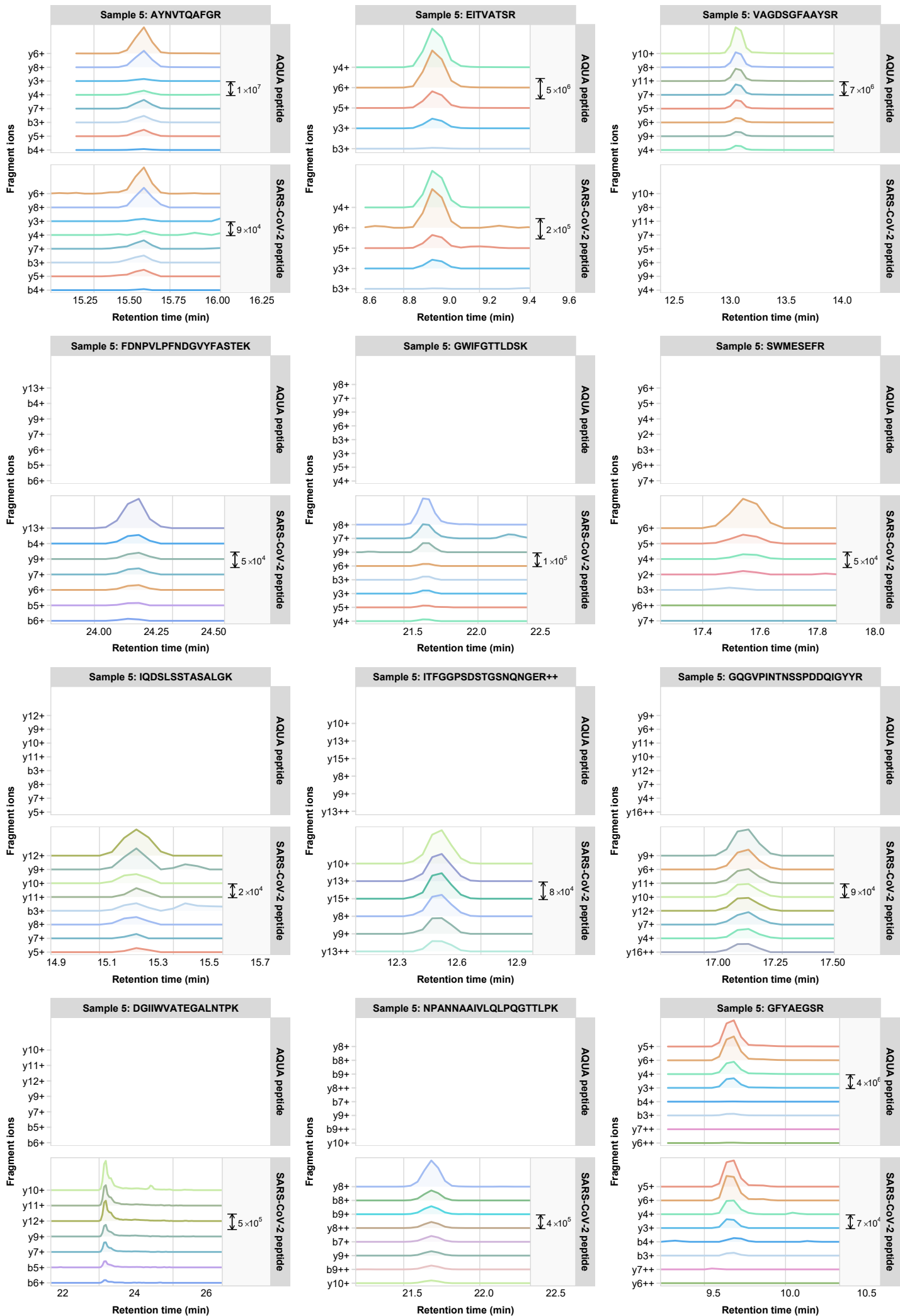

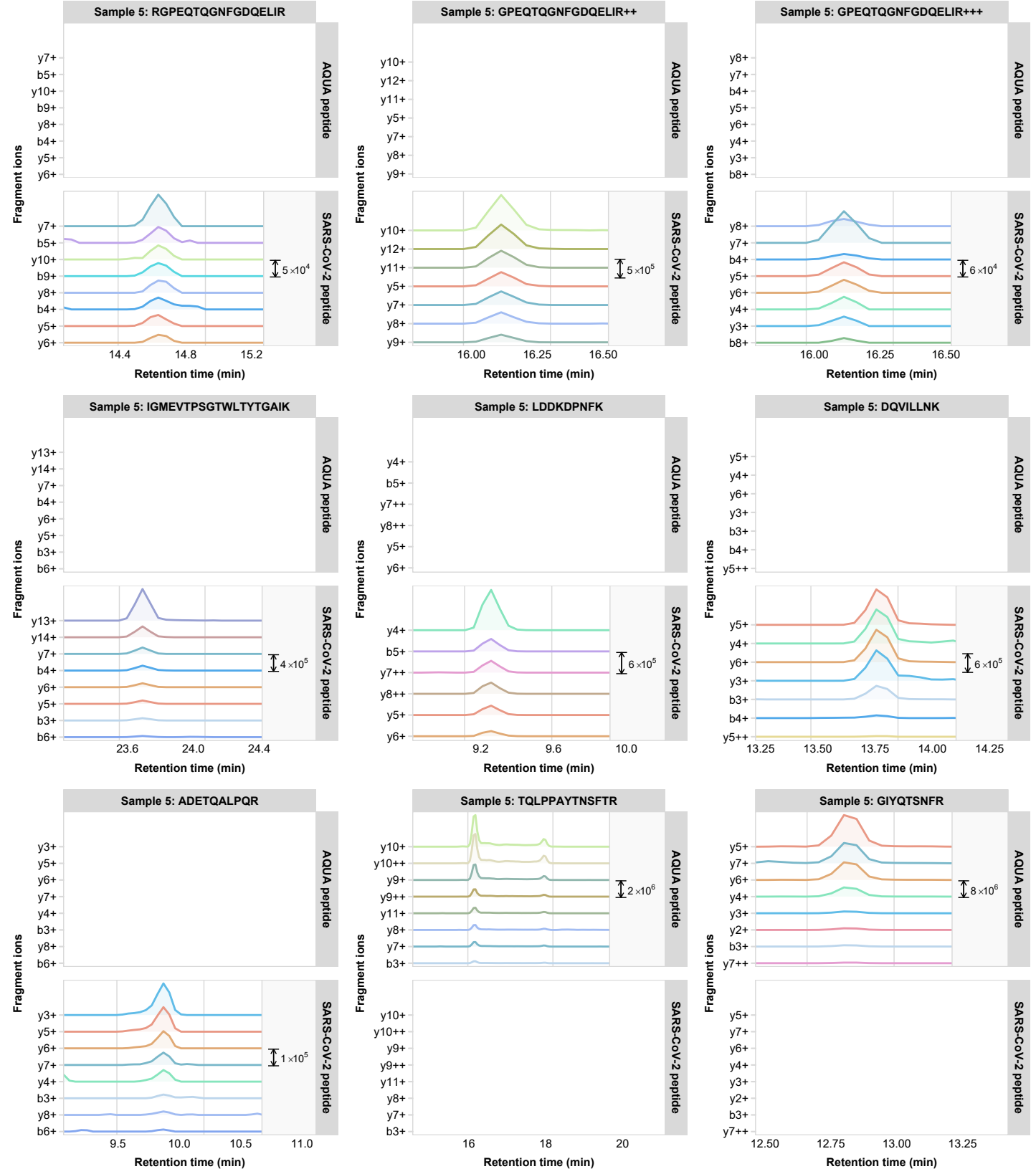

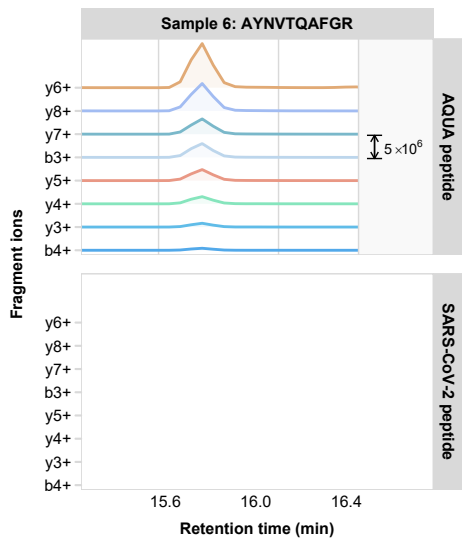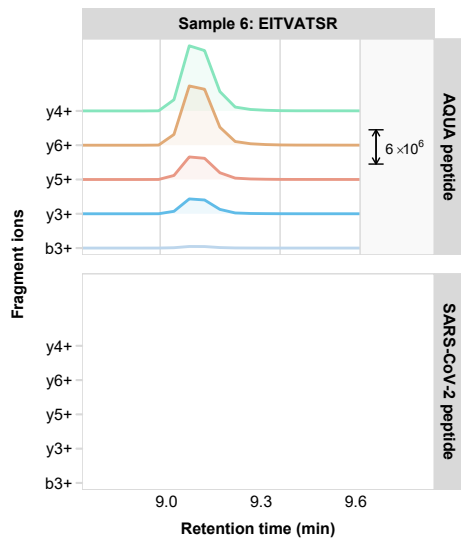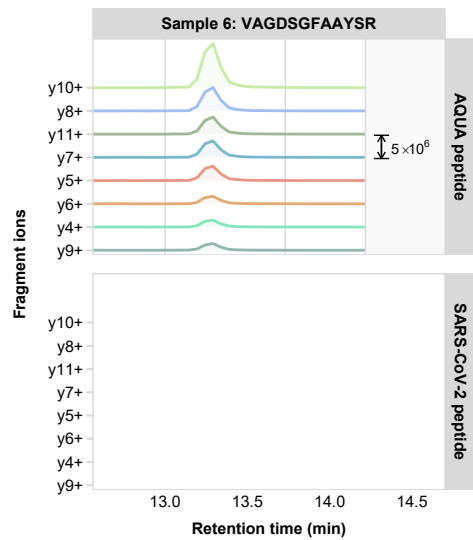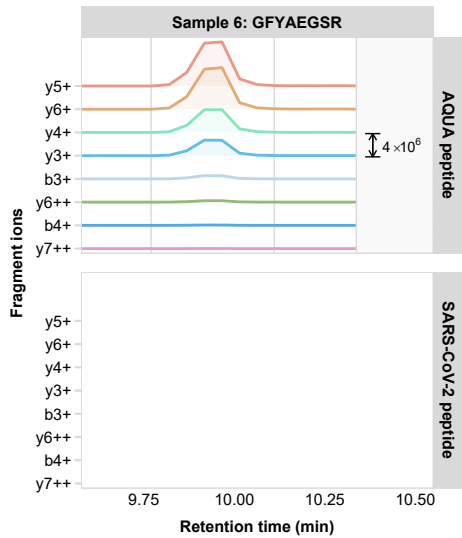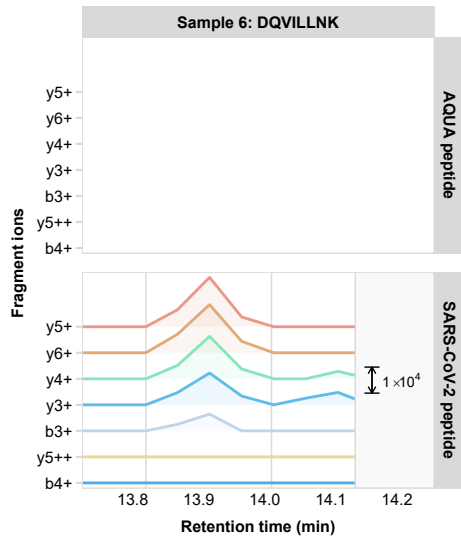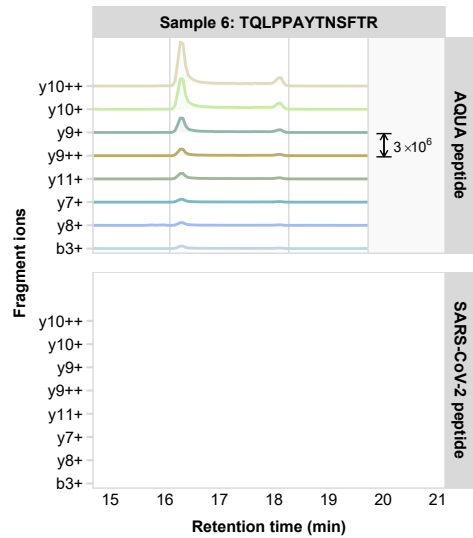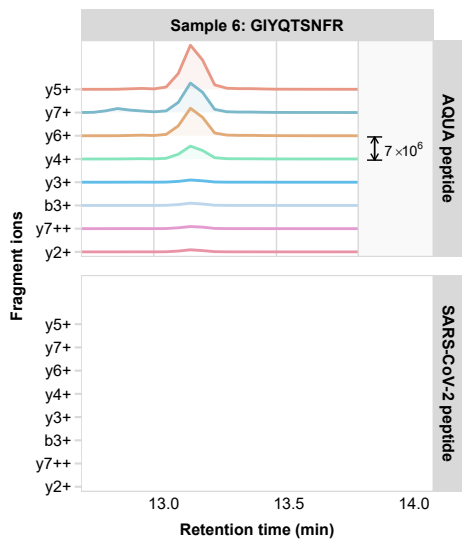

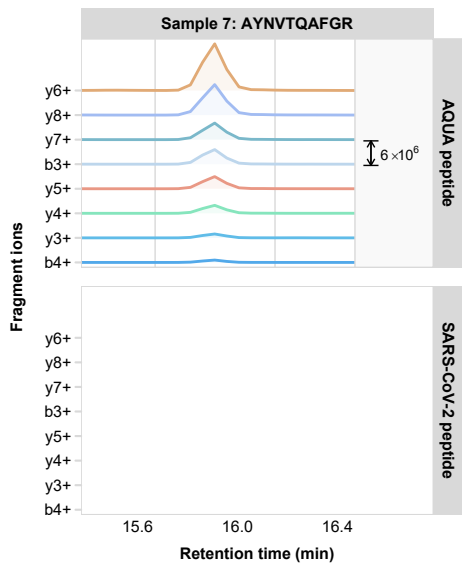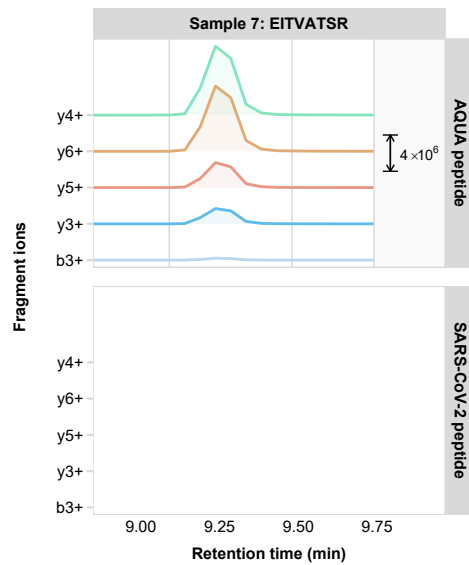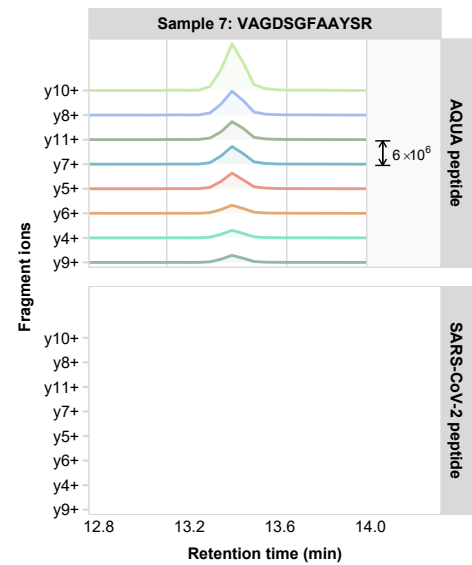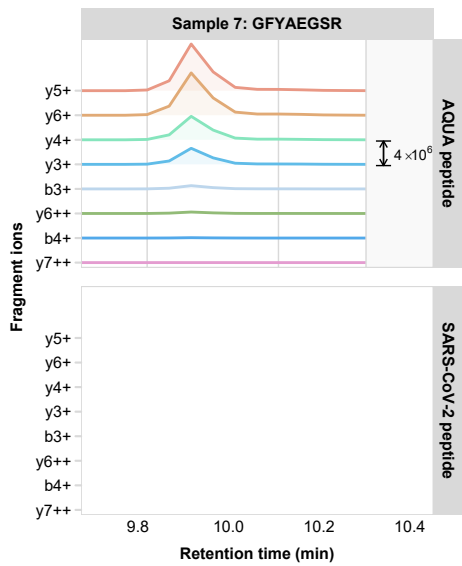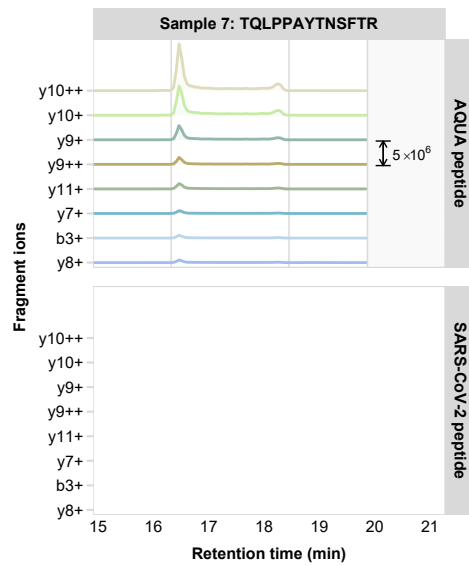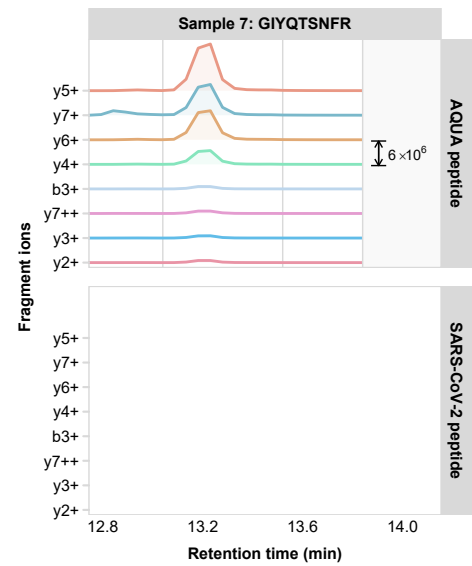

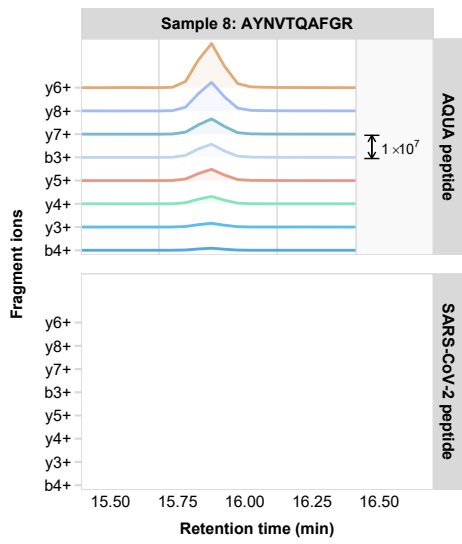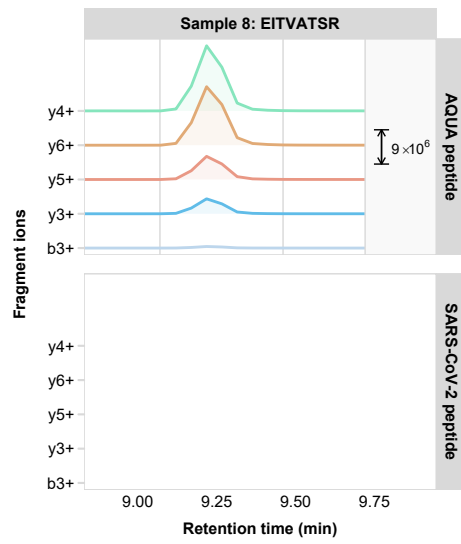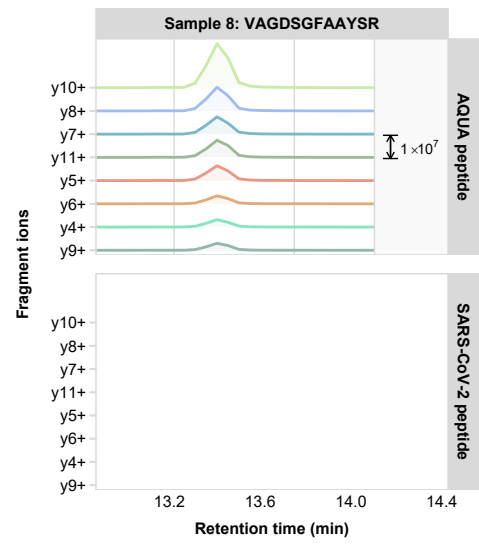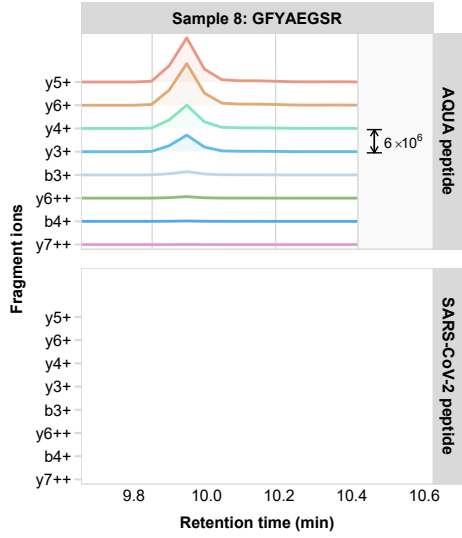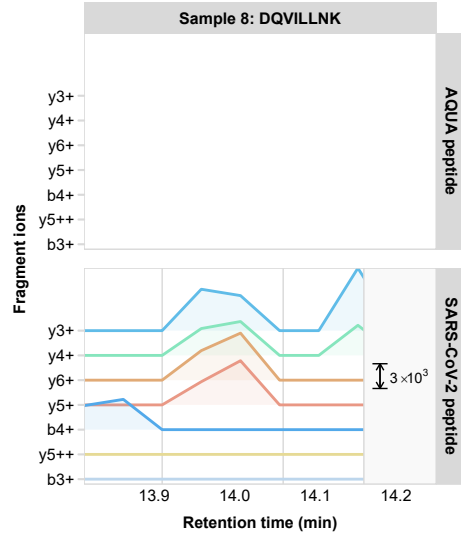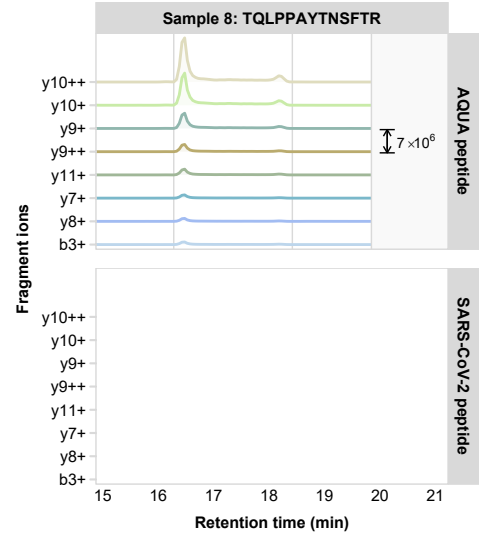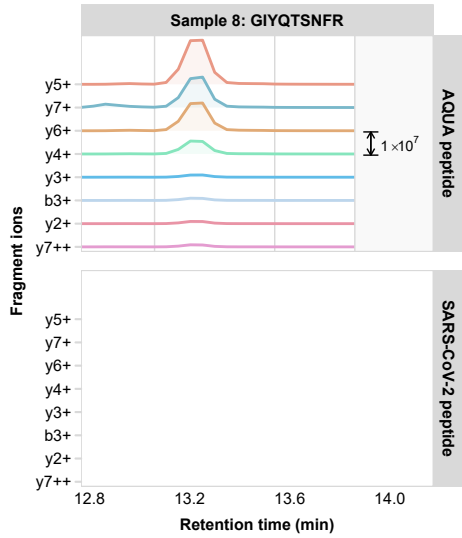

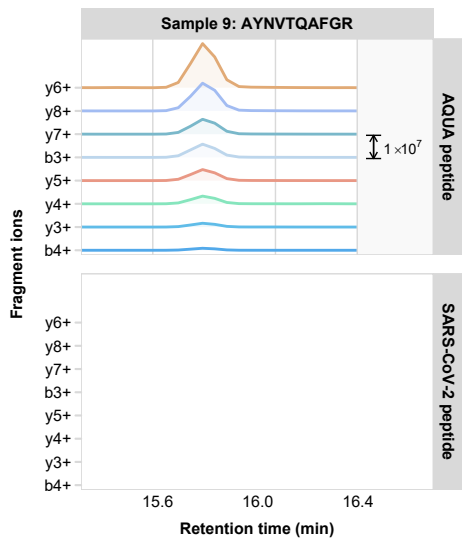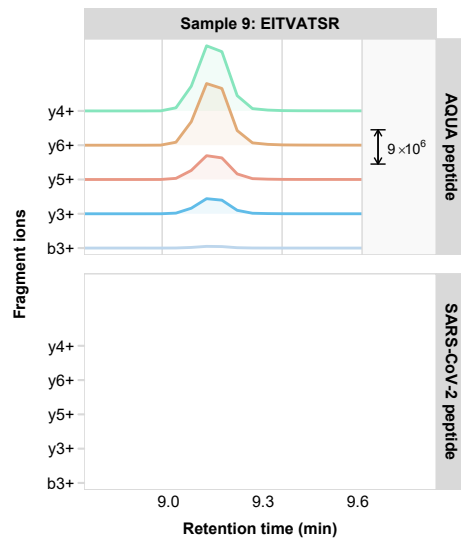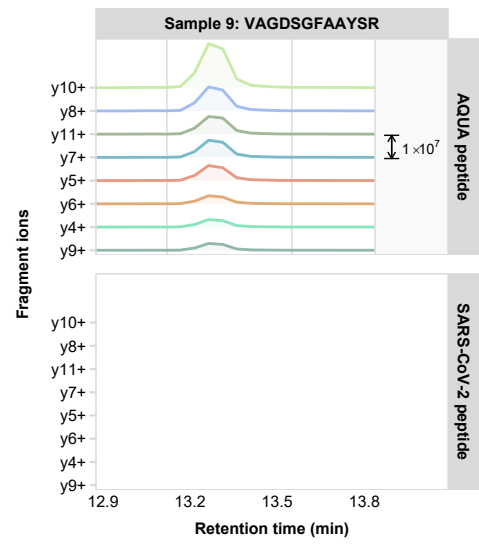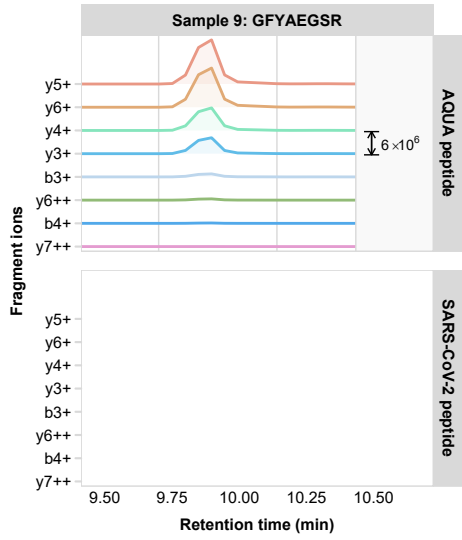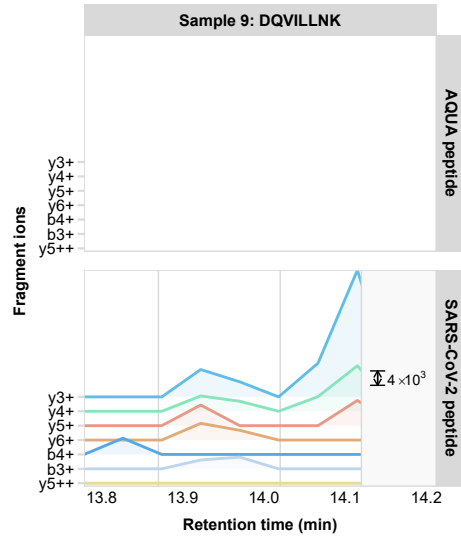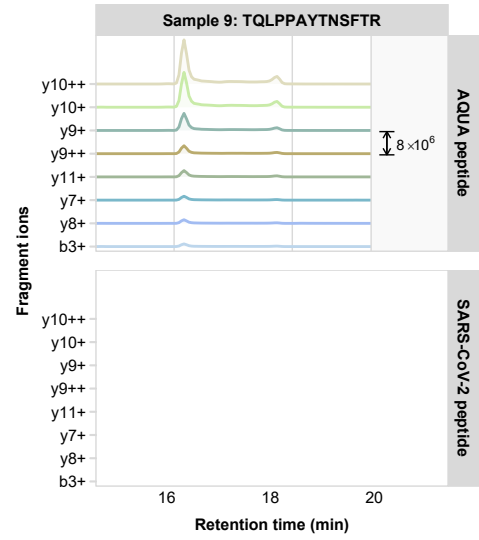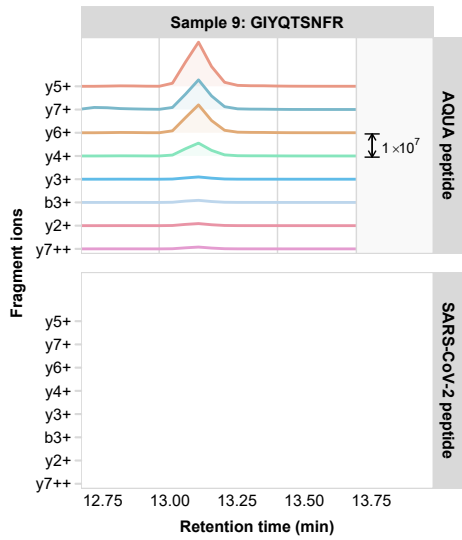

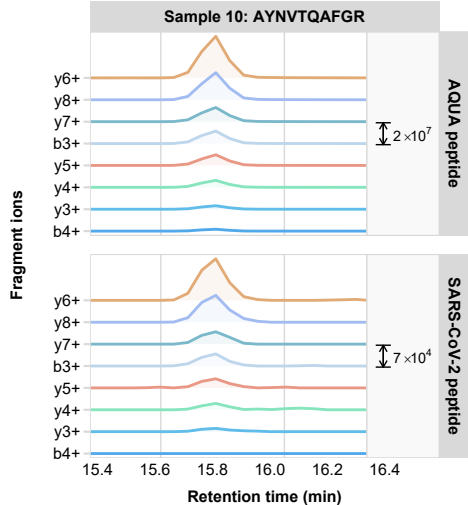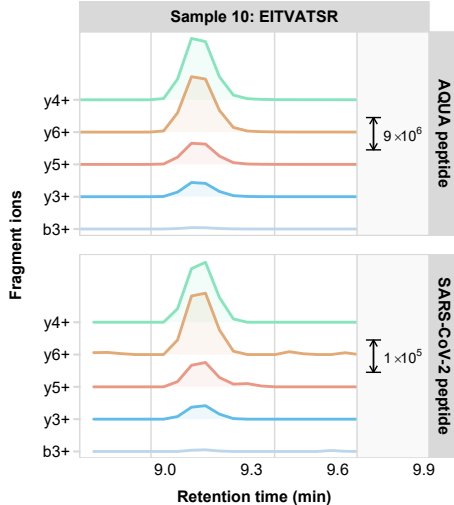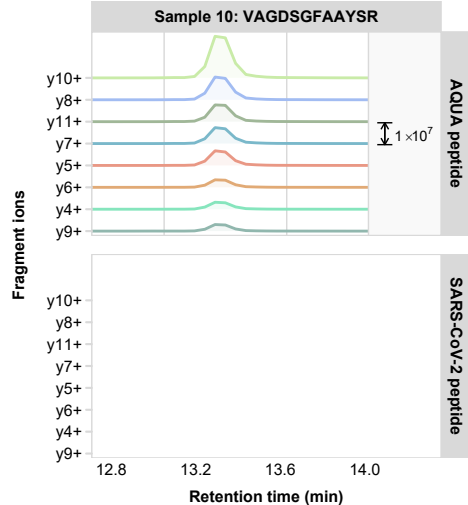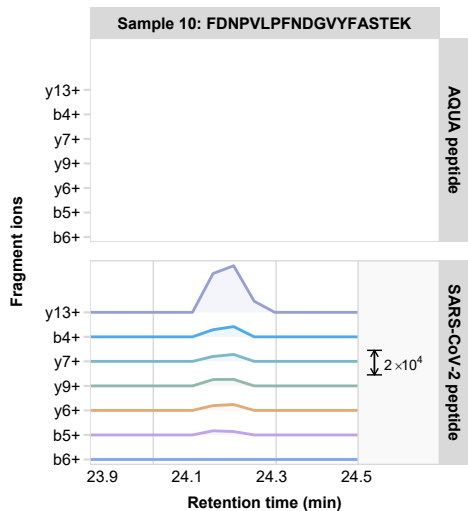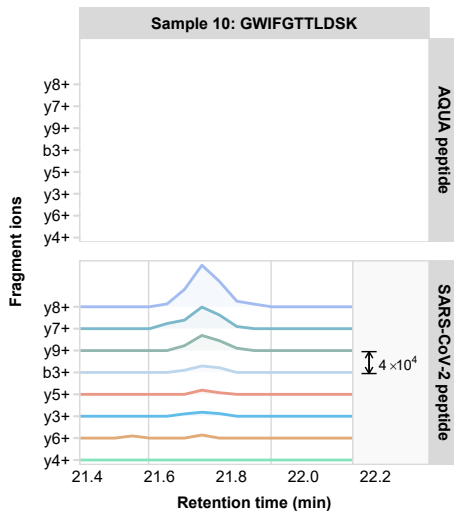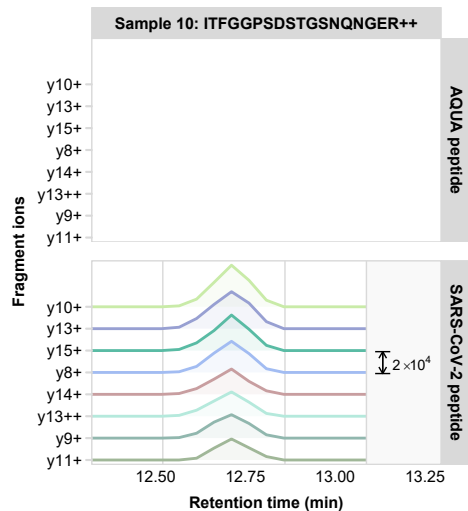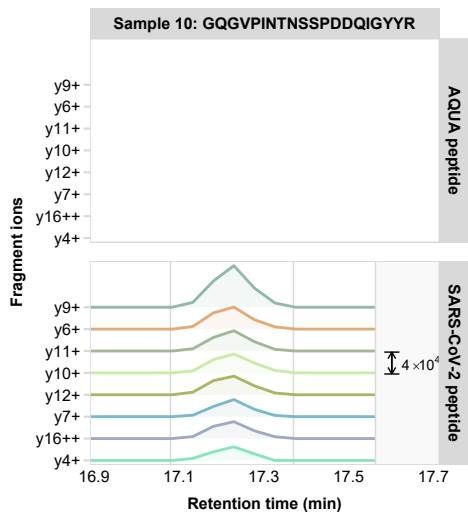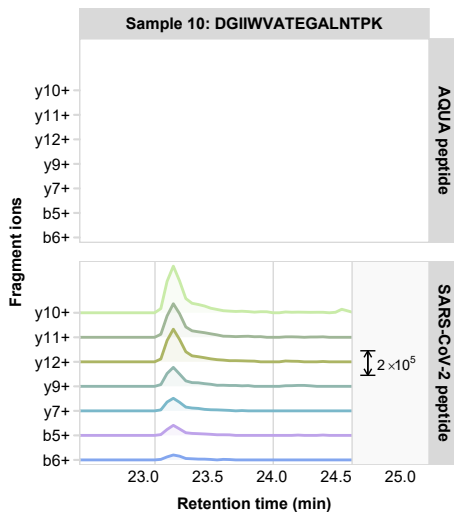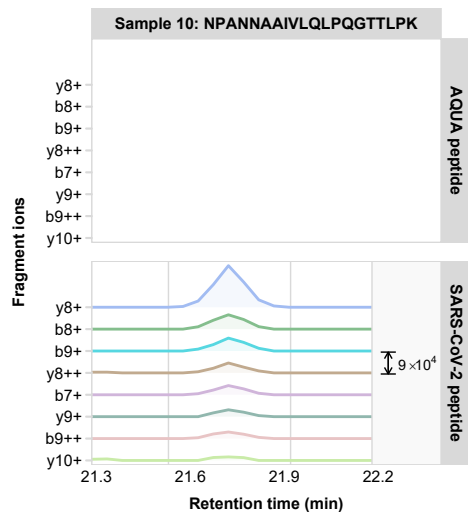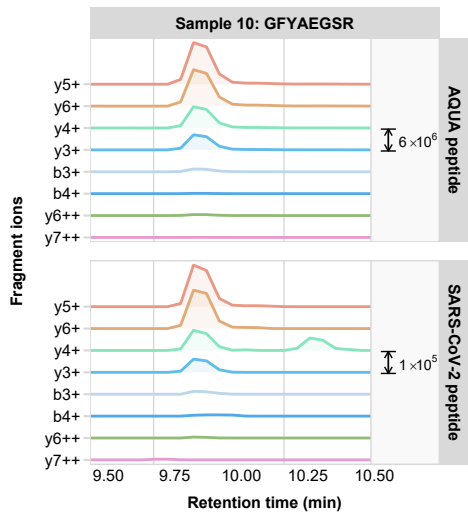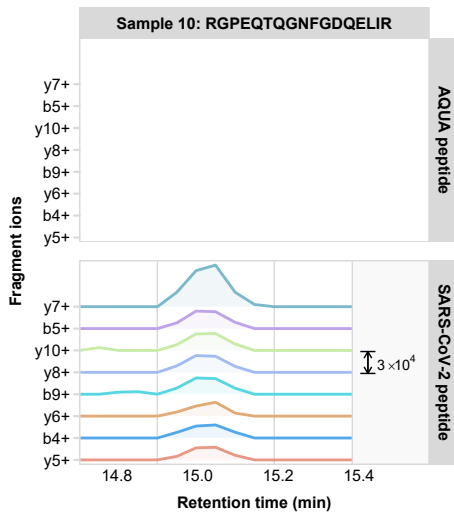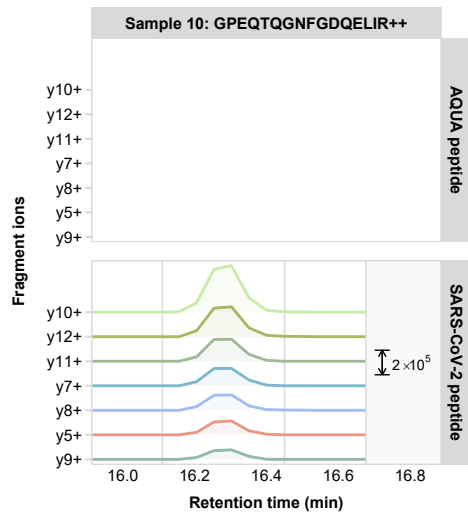

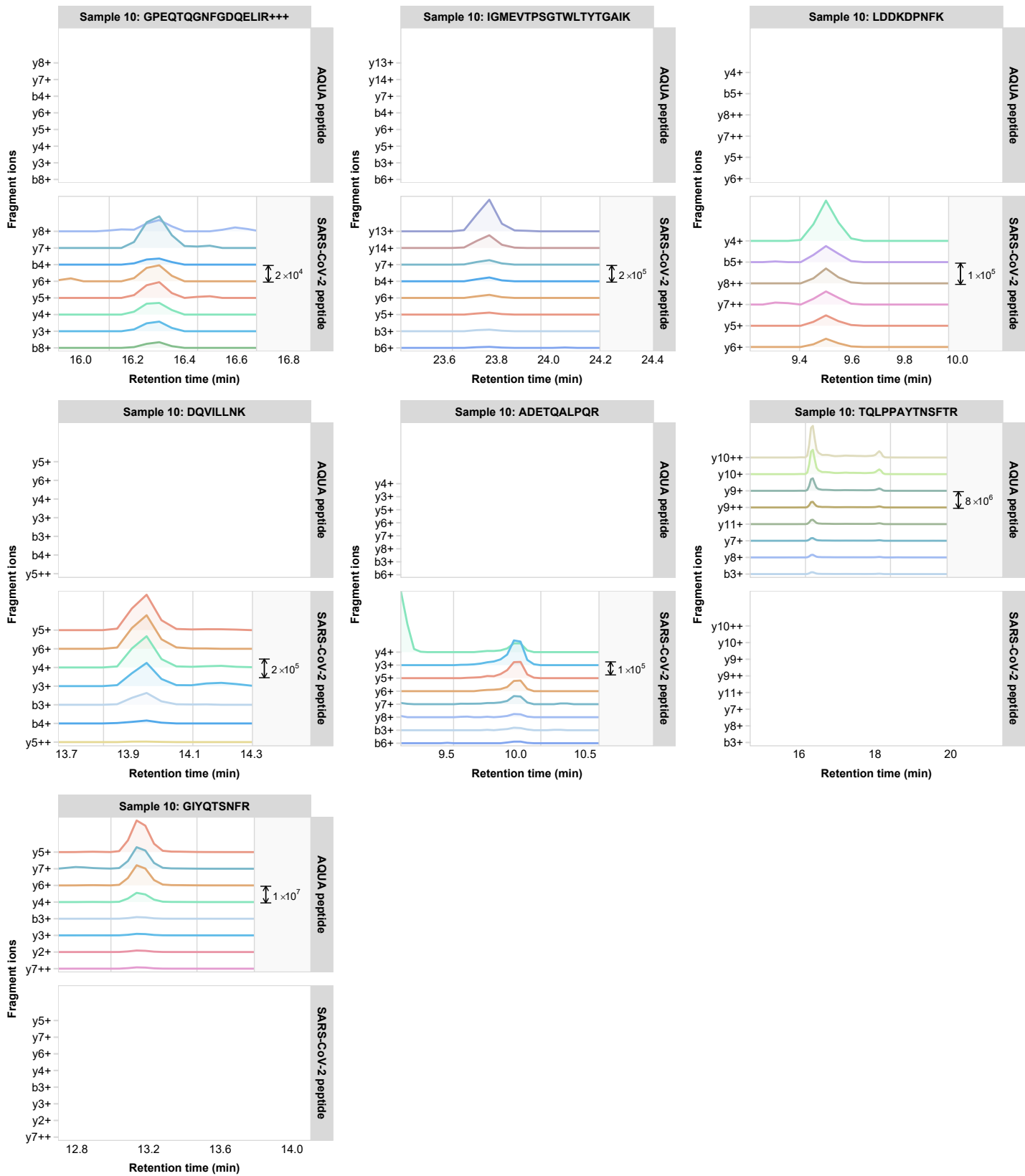



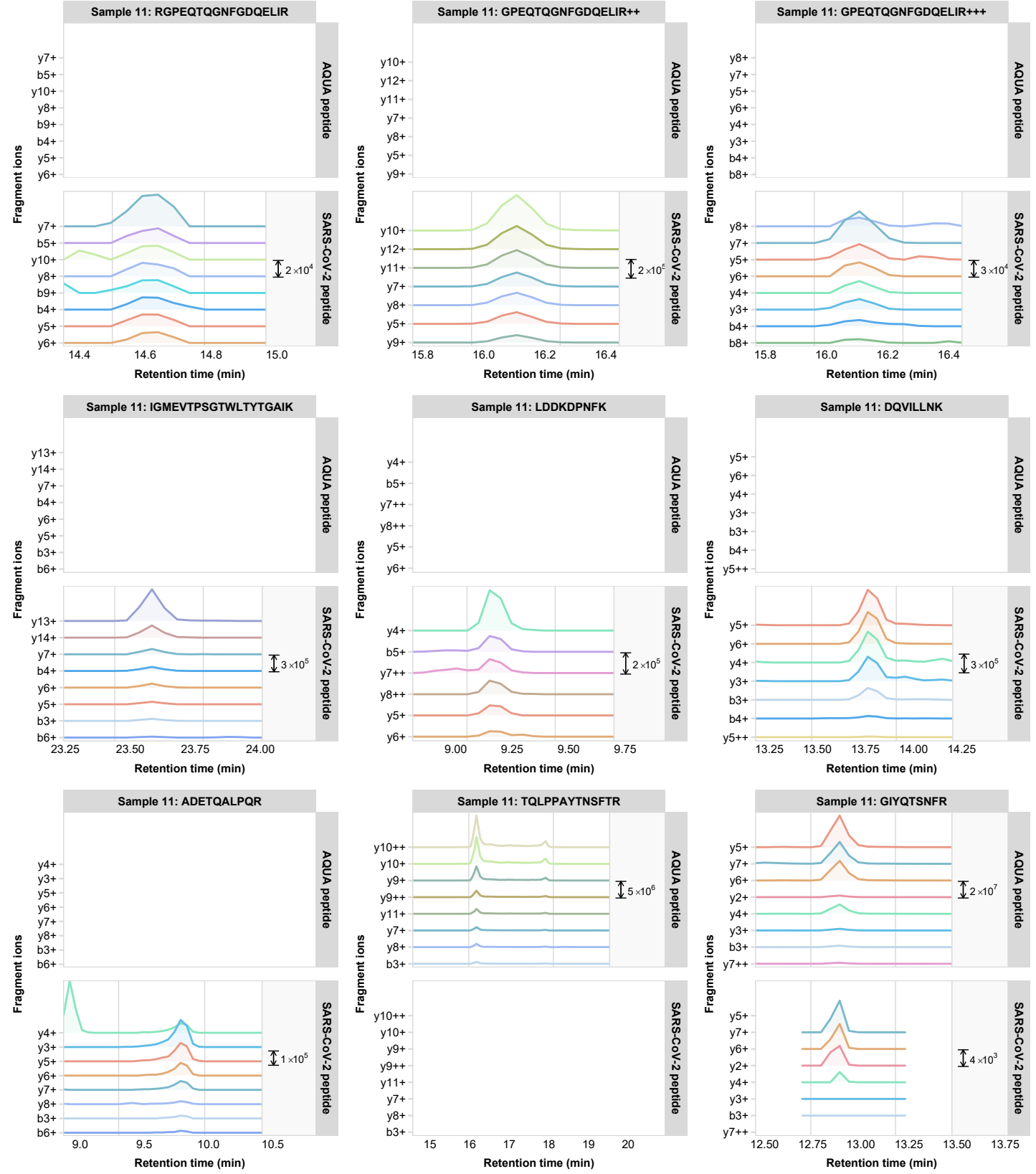

Supplement: S7 File — (PDF) [file pone.0259165.s013.pdf]

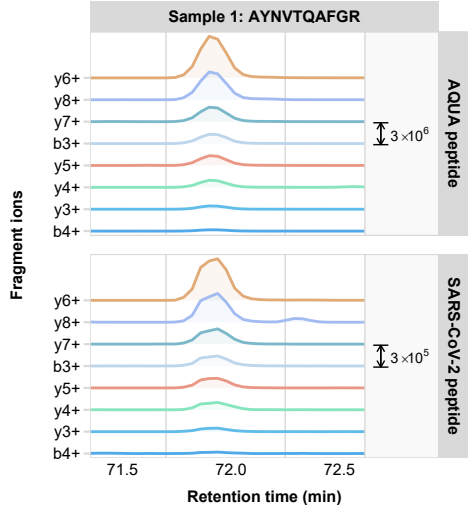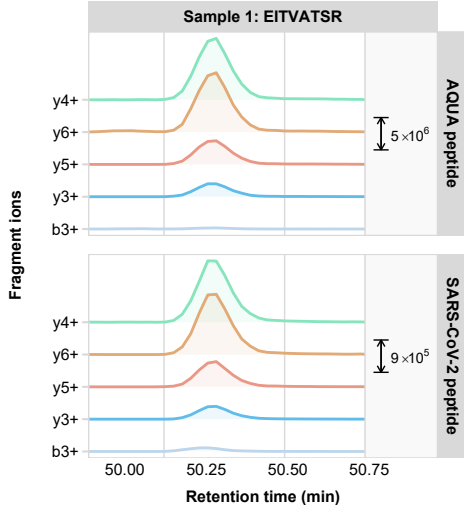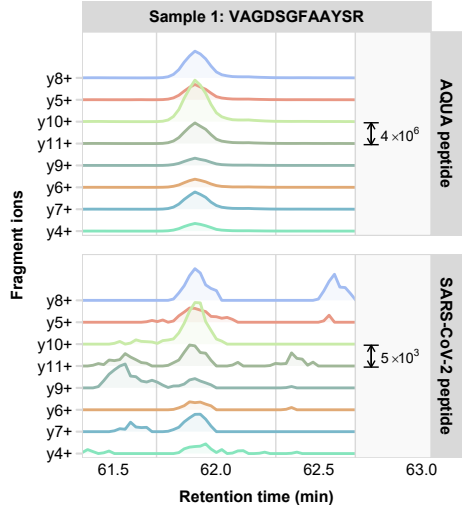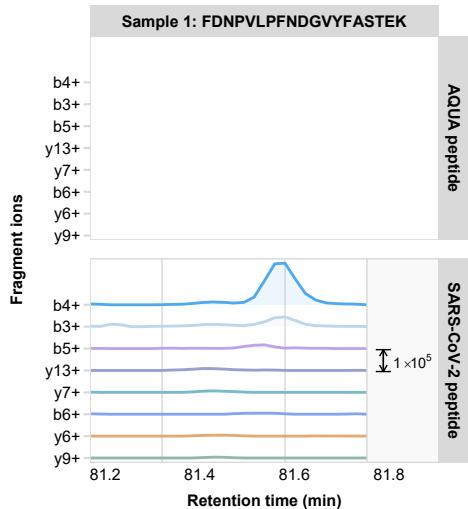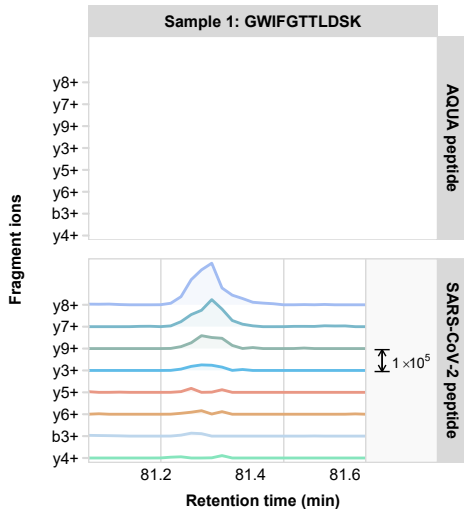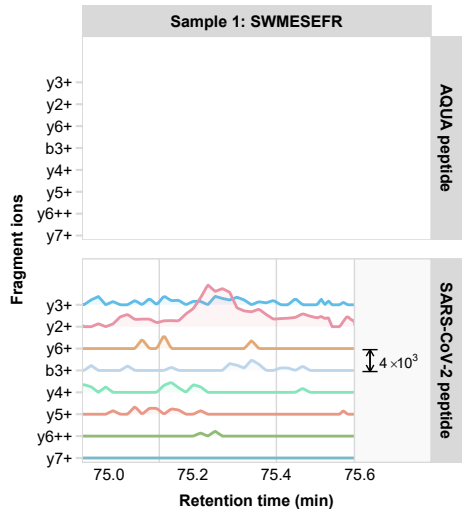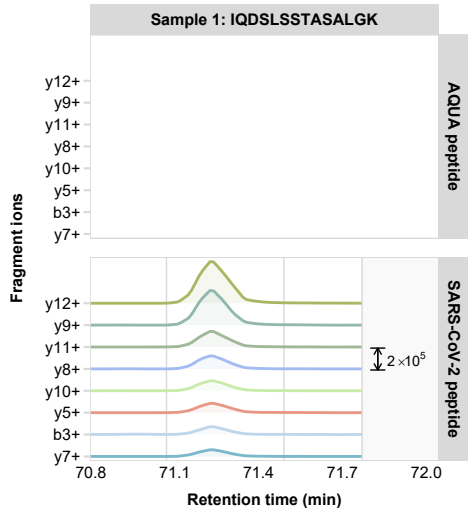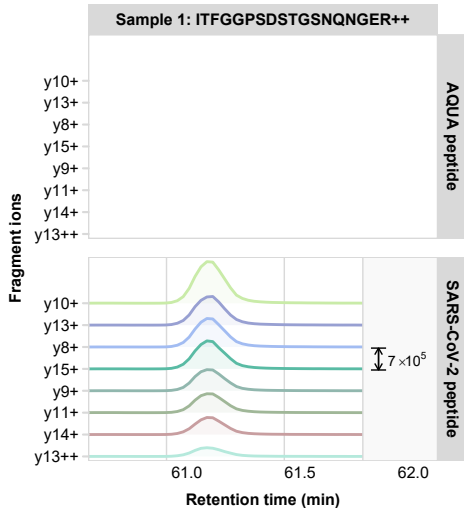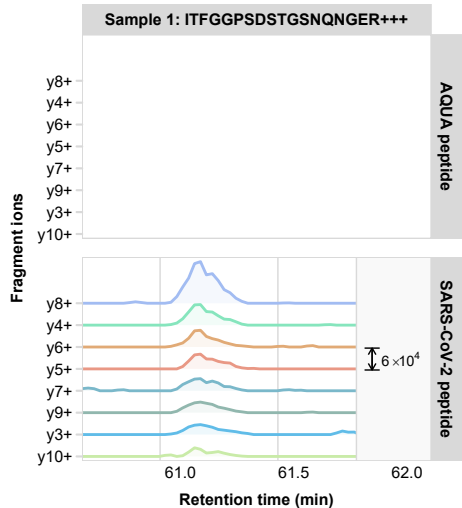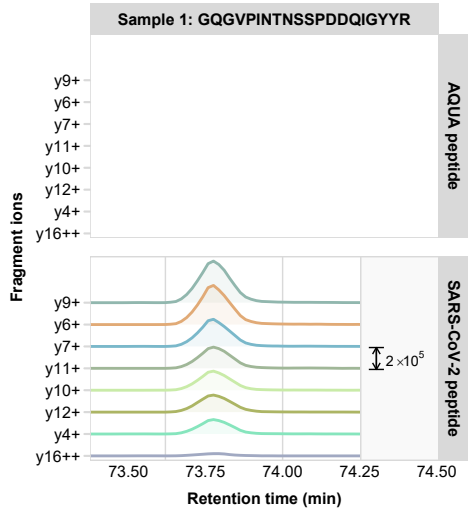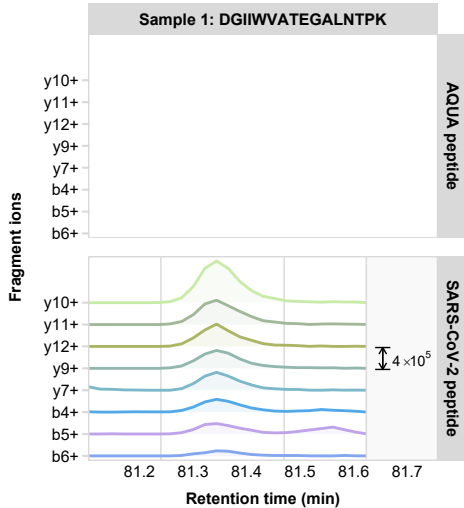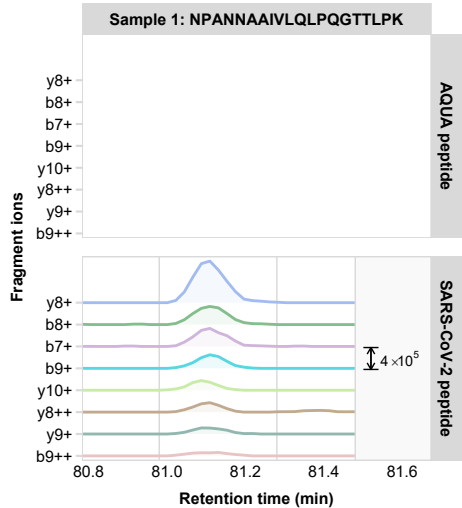

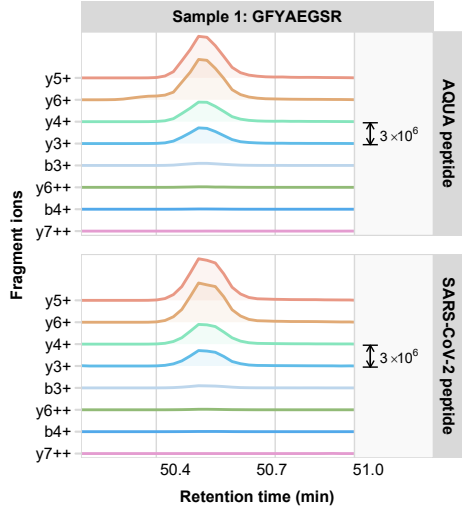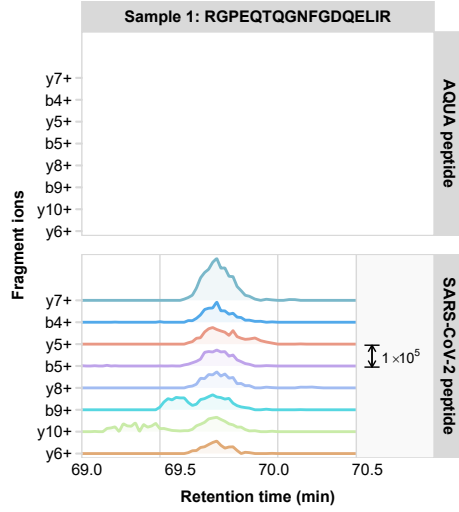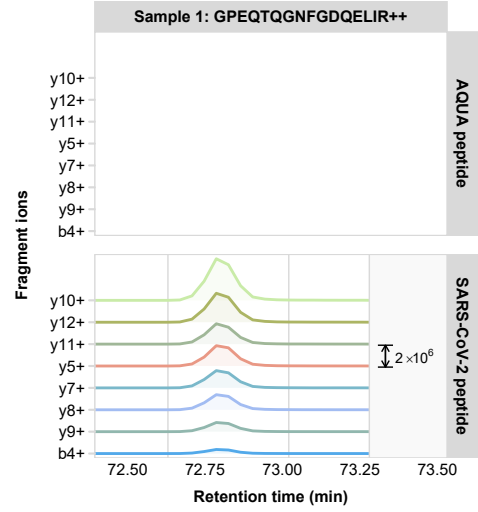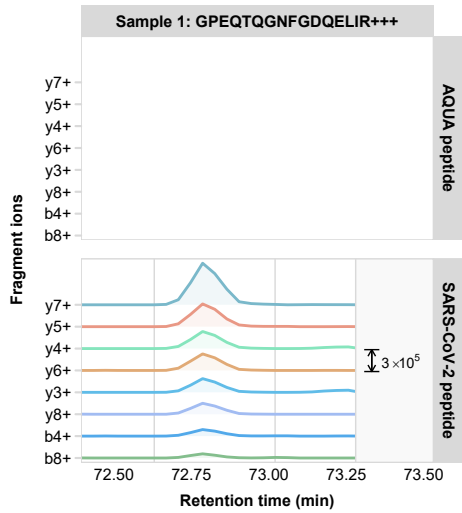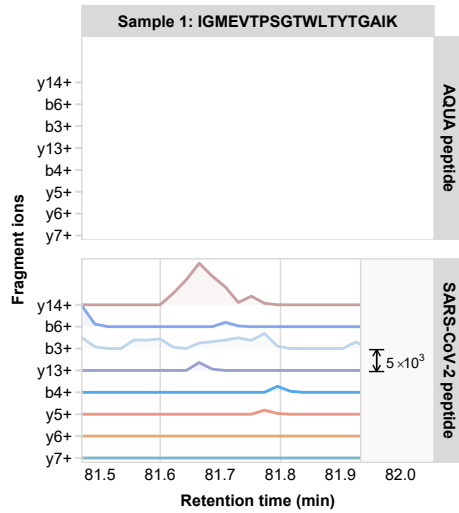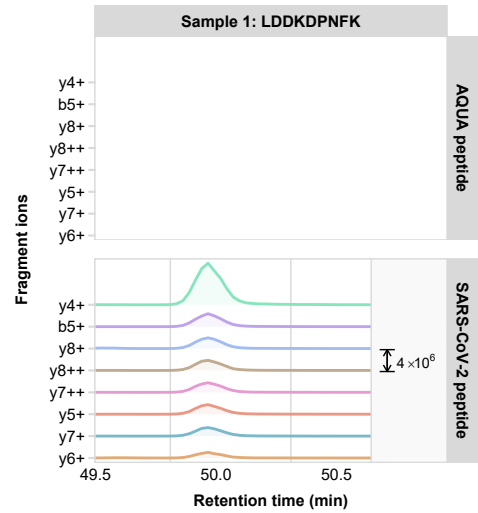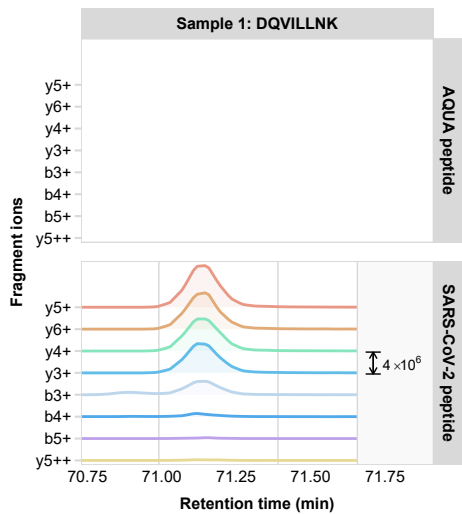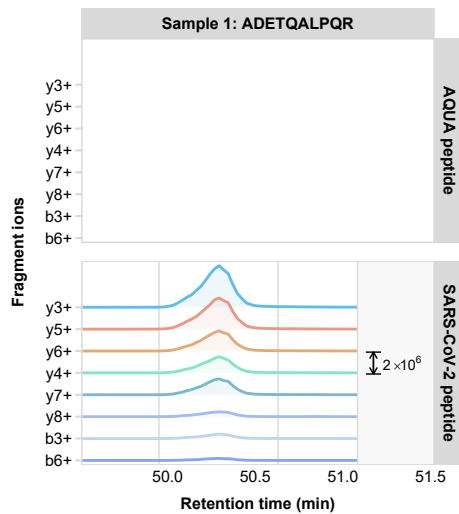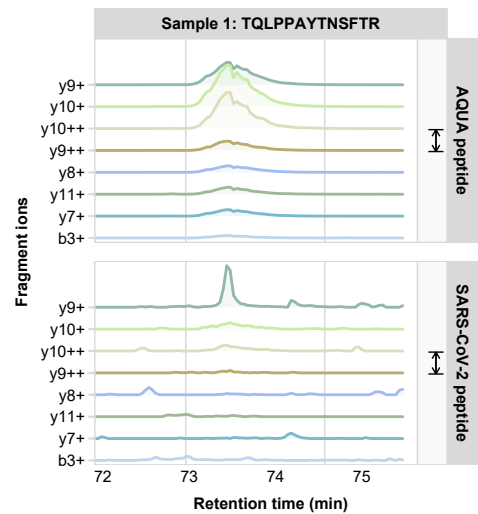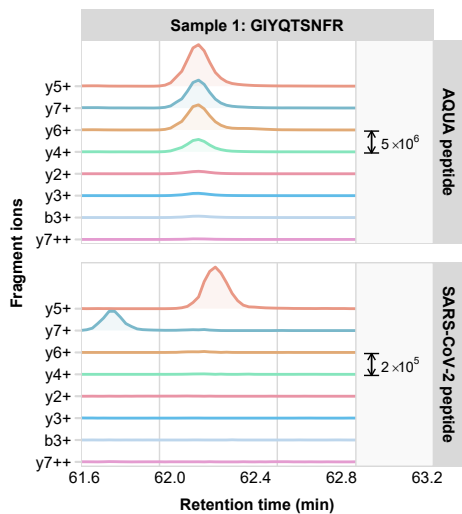

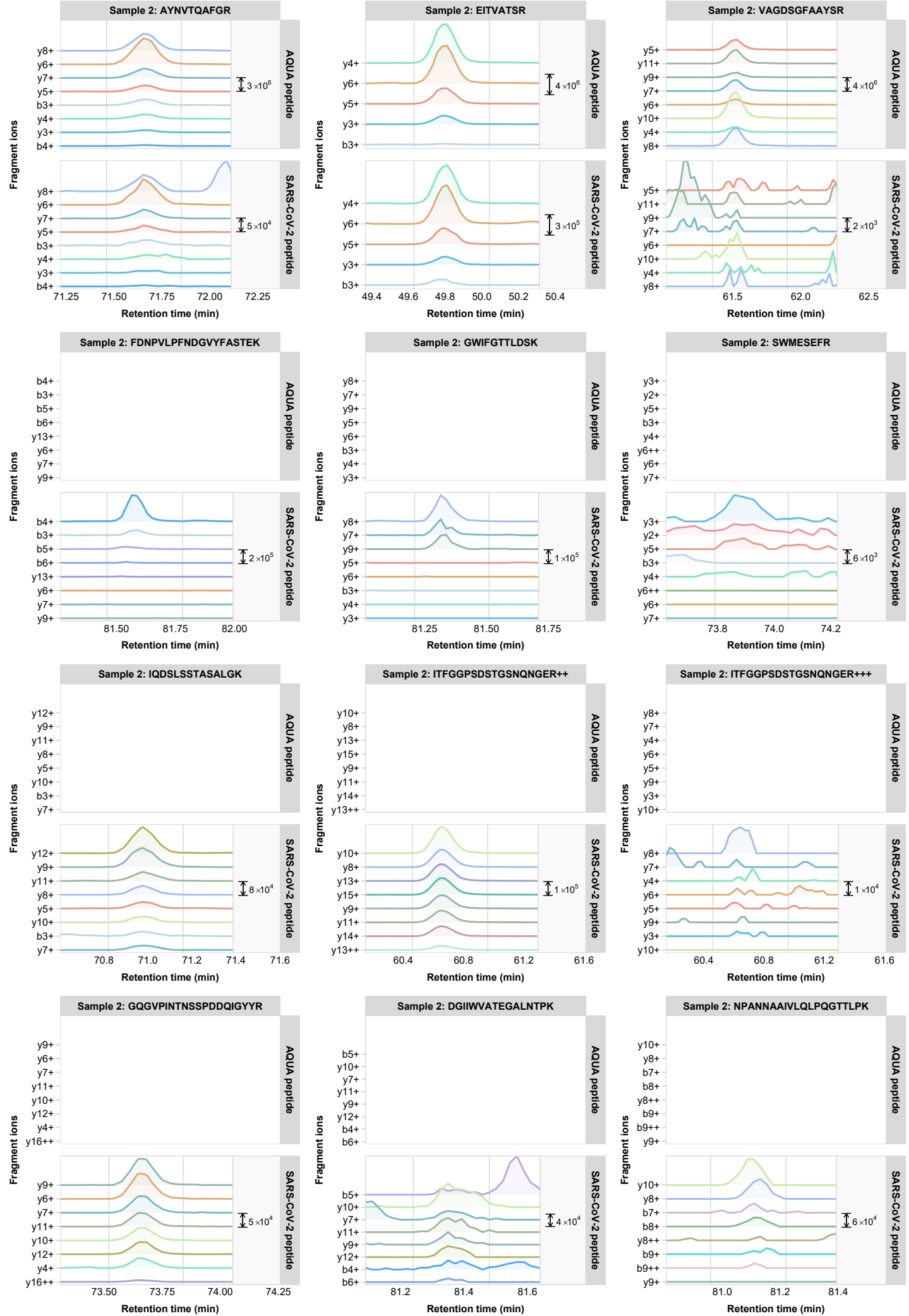

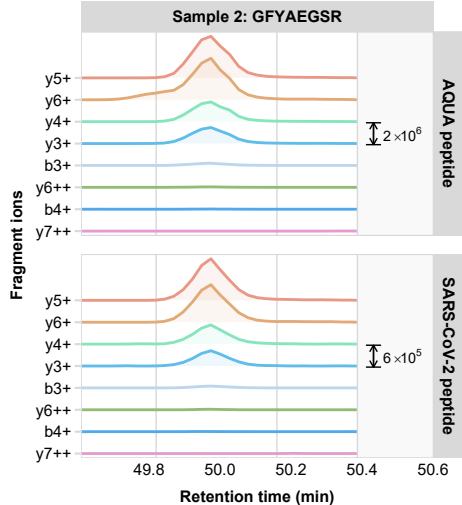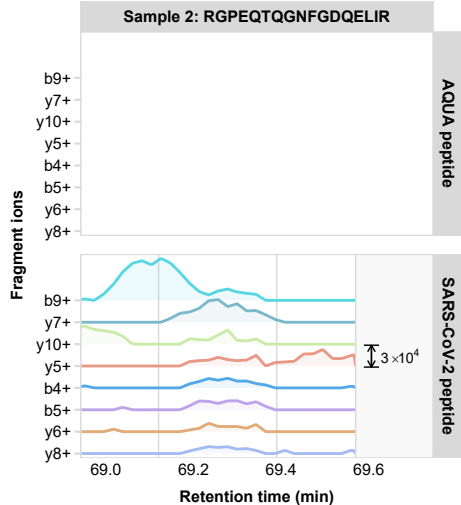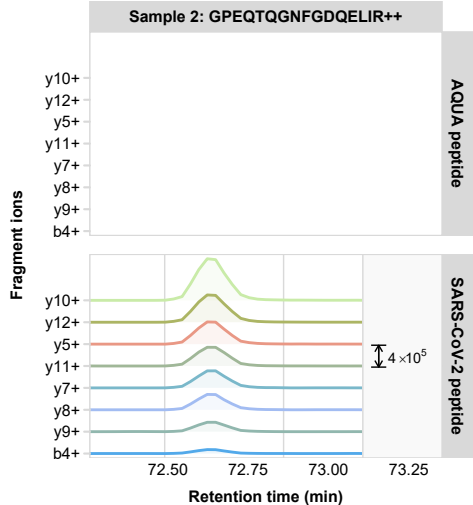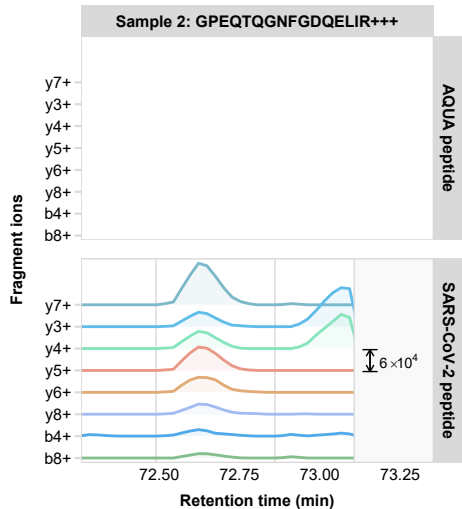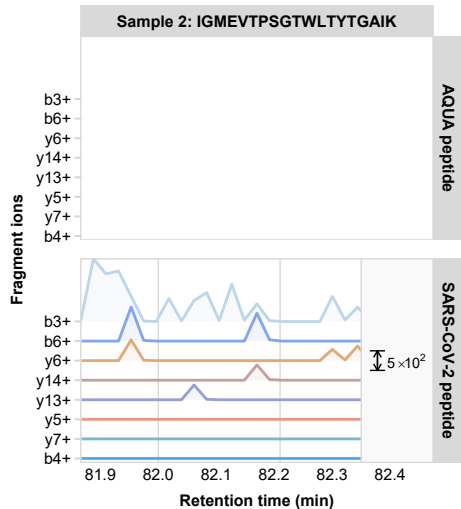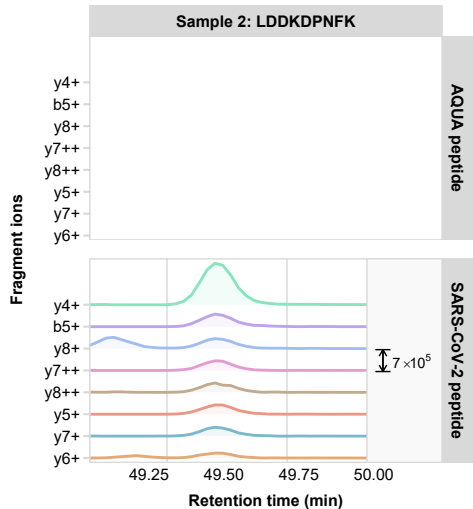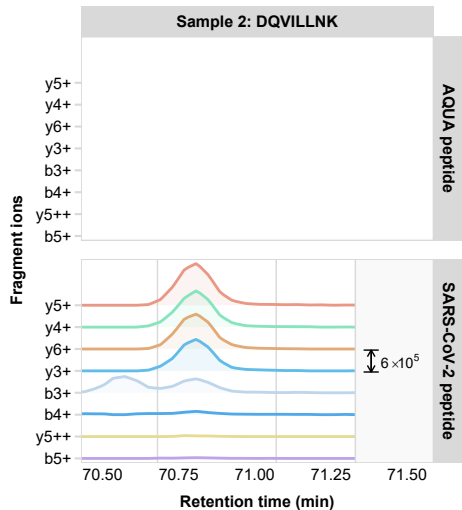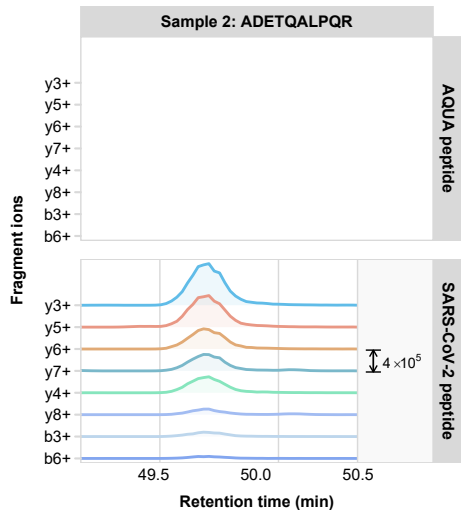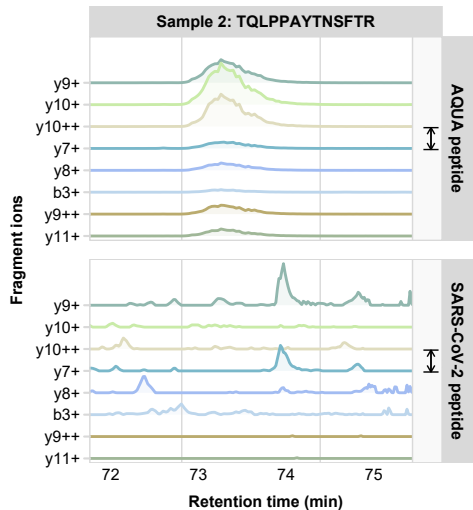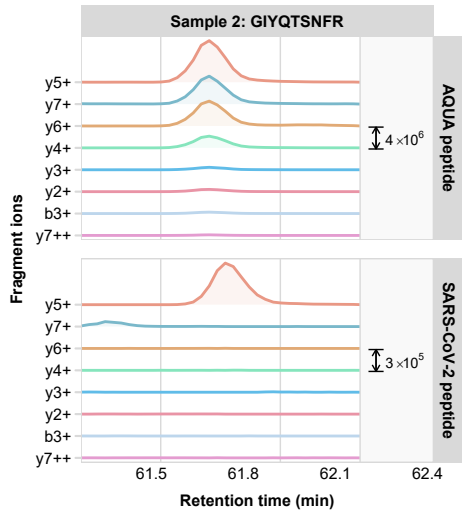

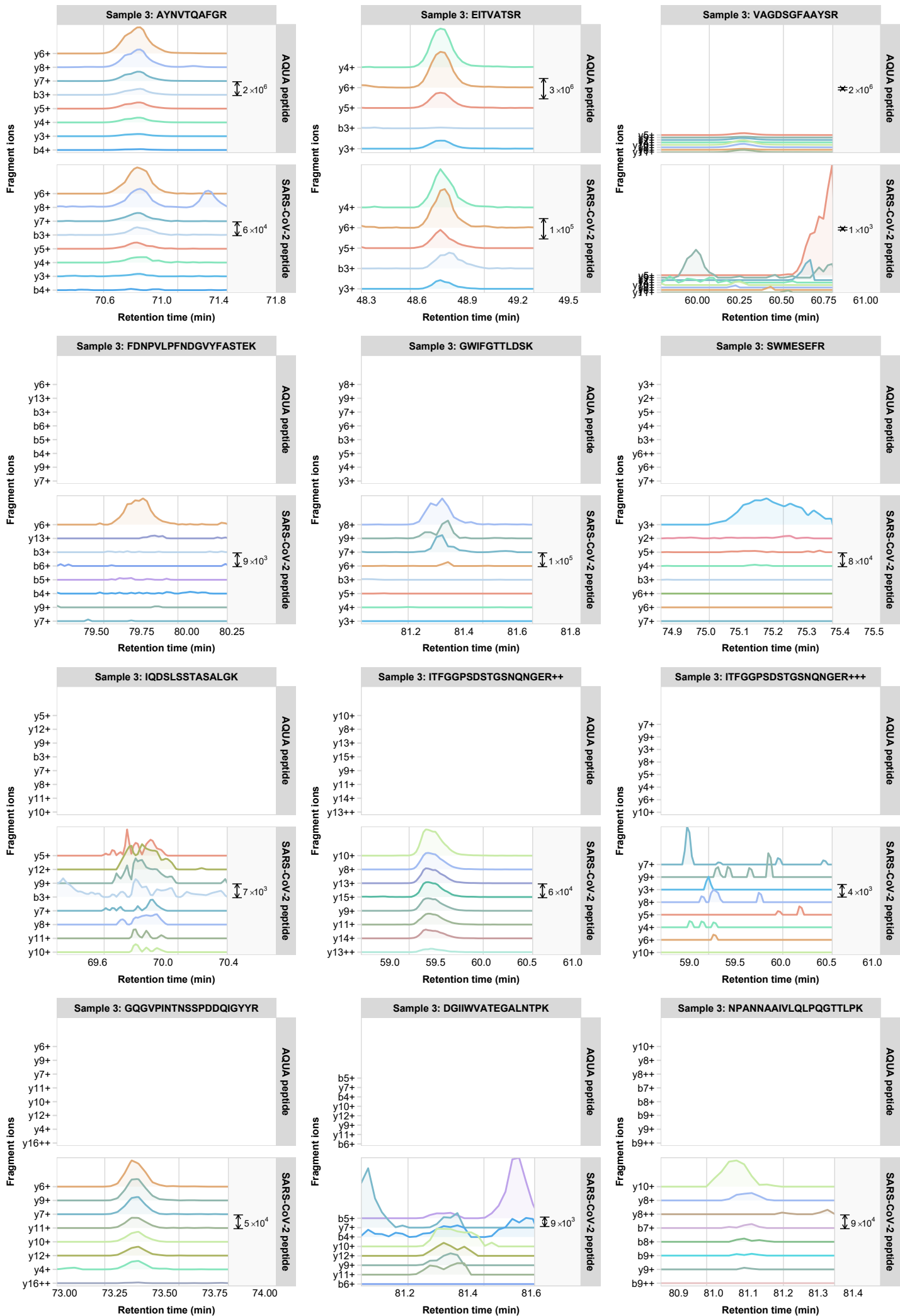

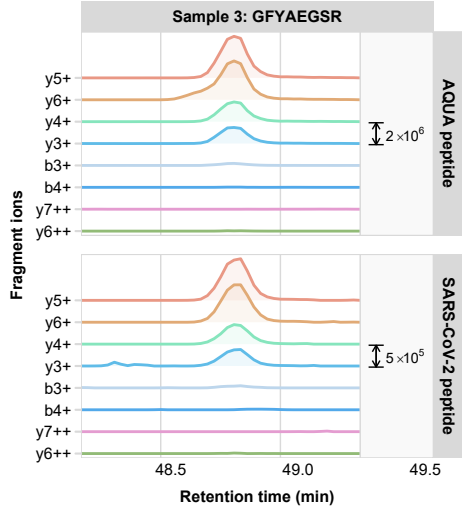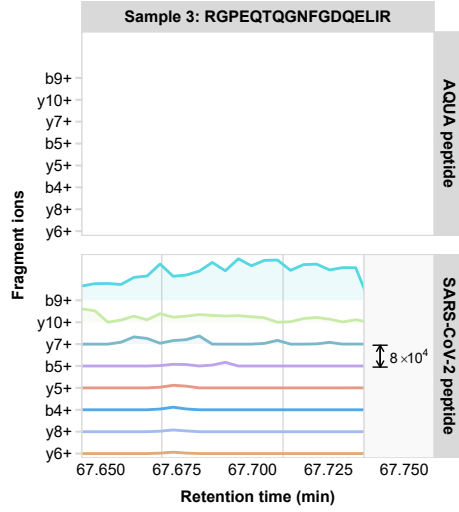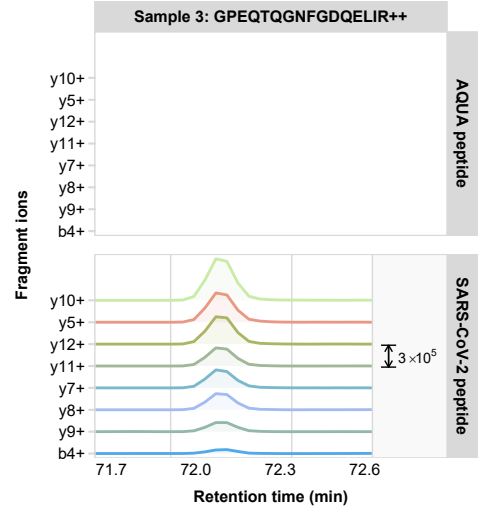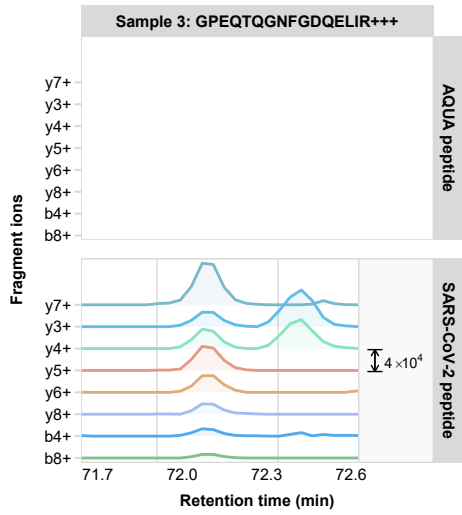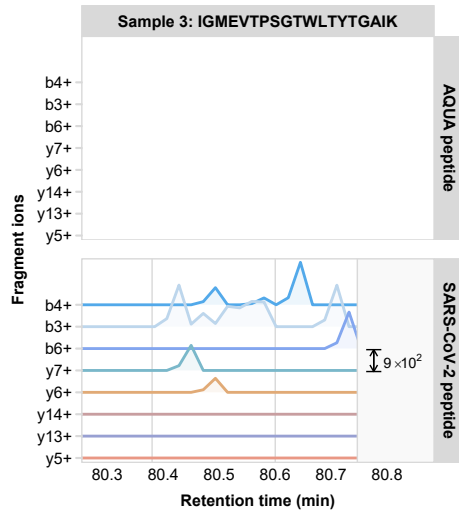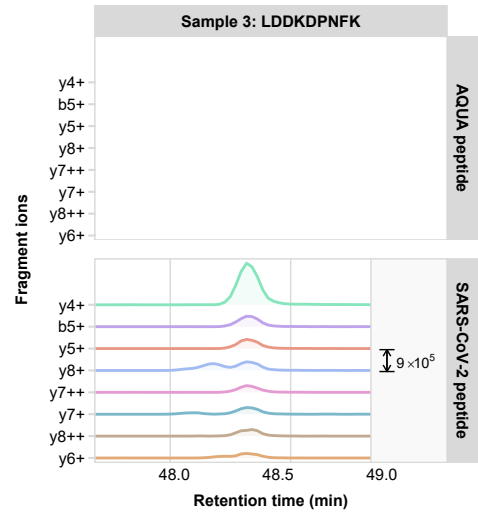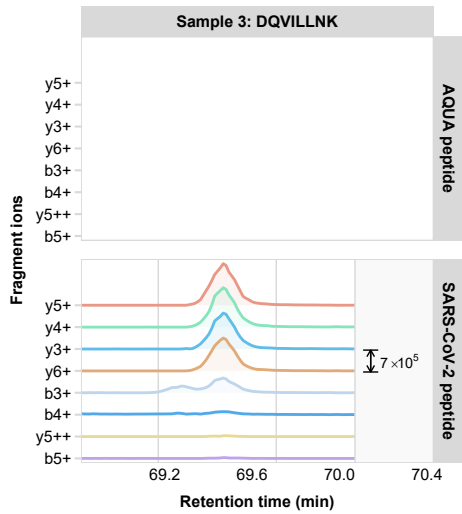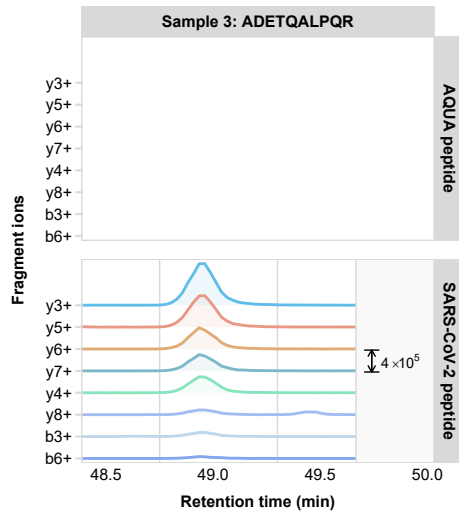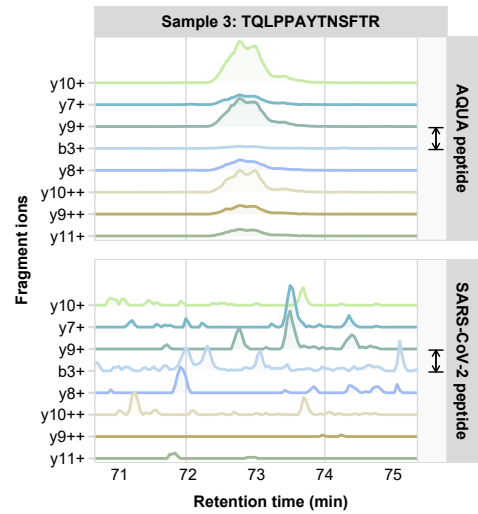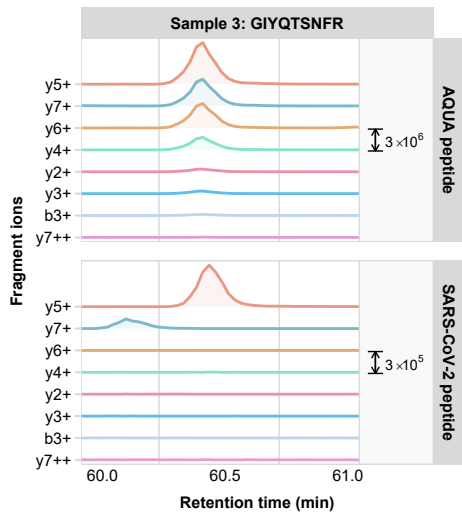

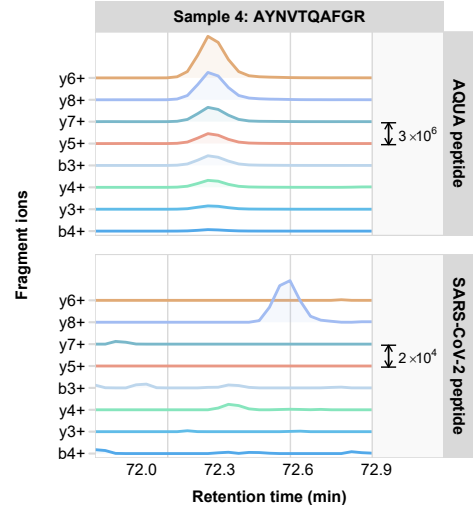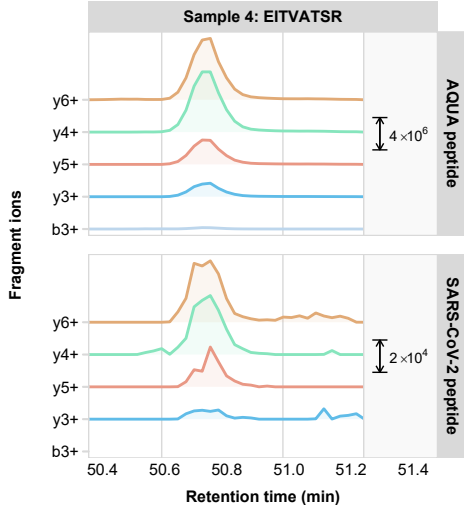

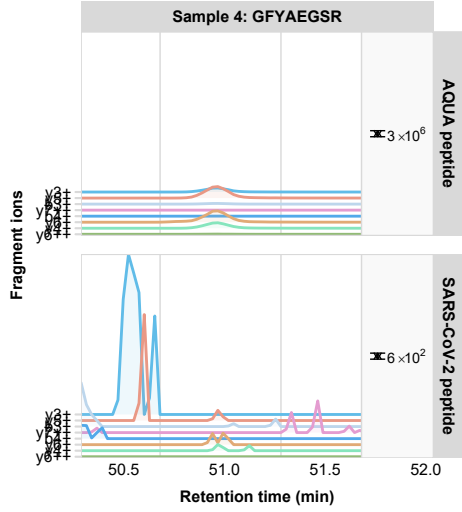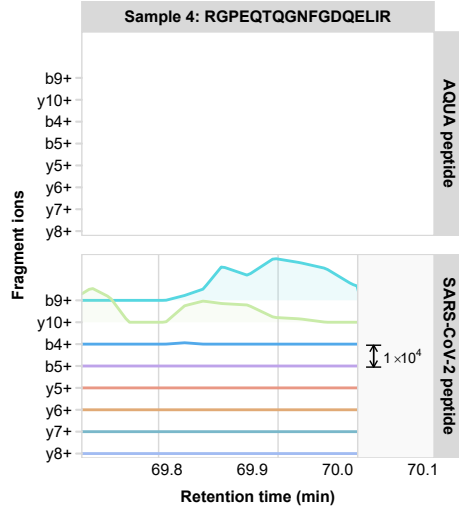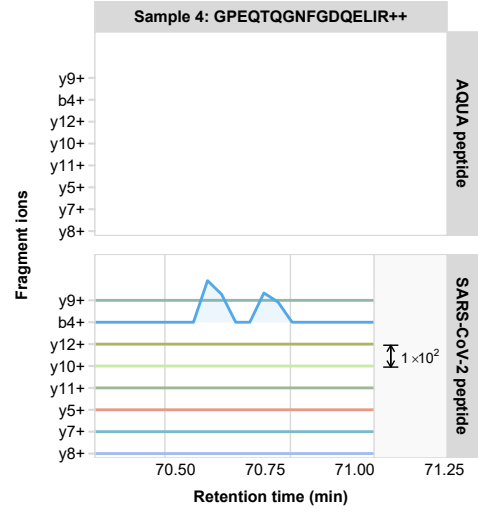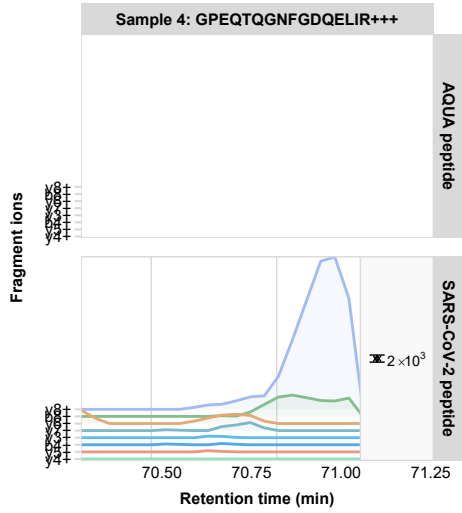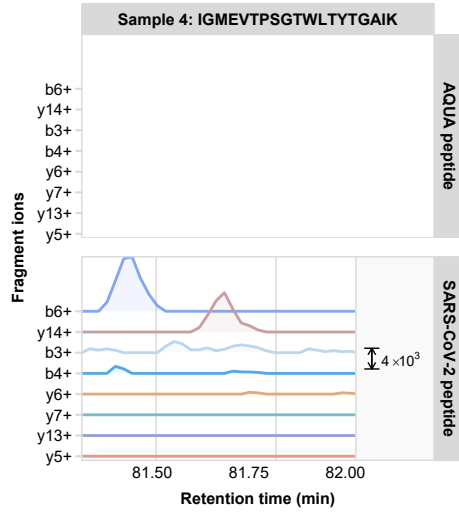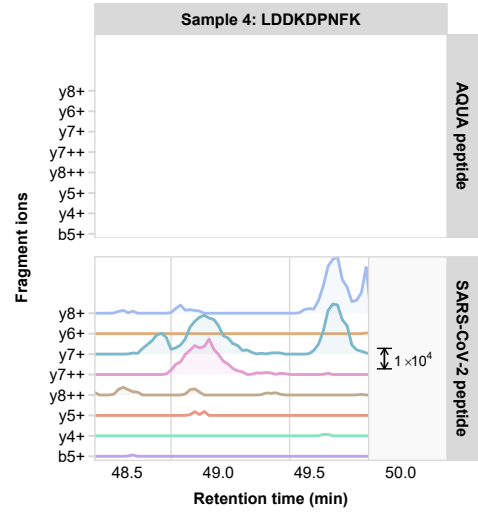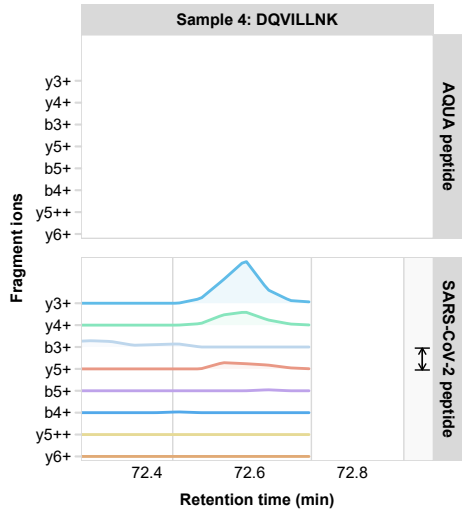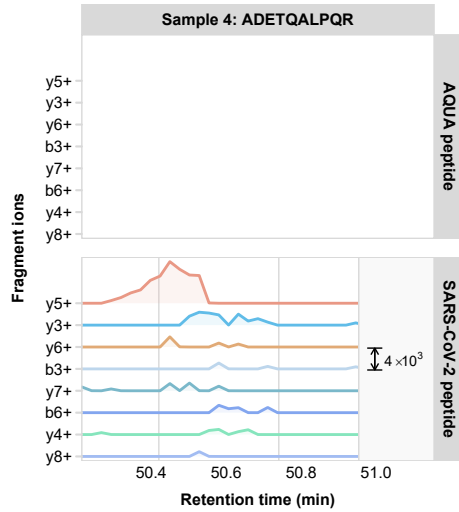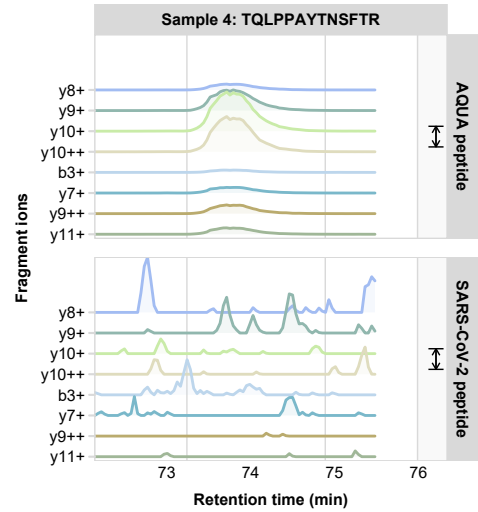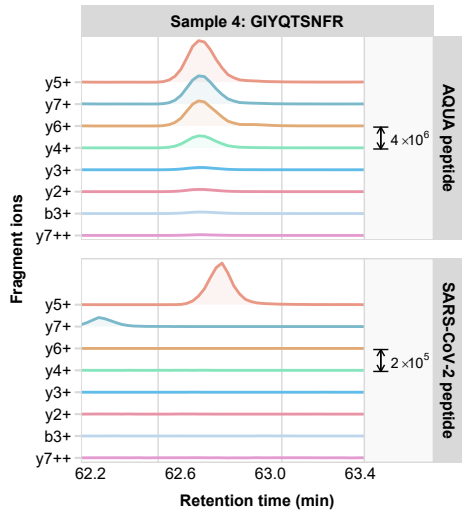

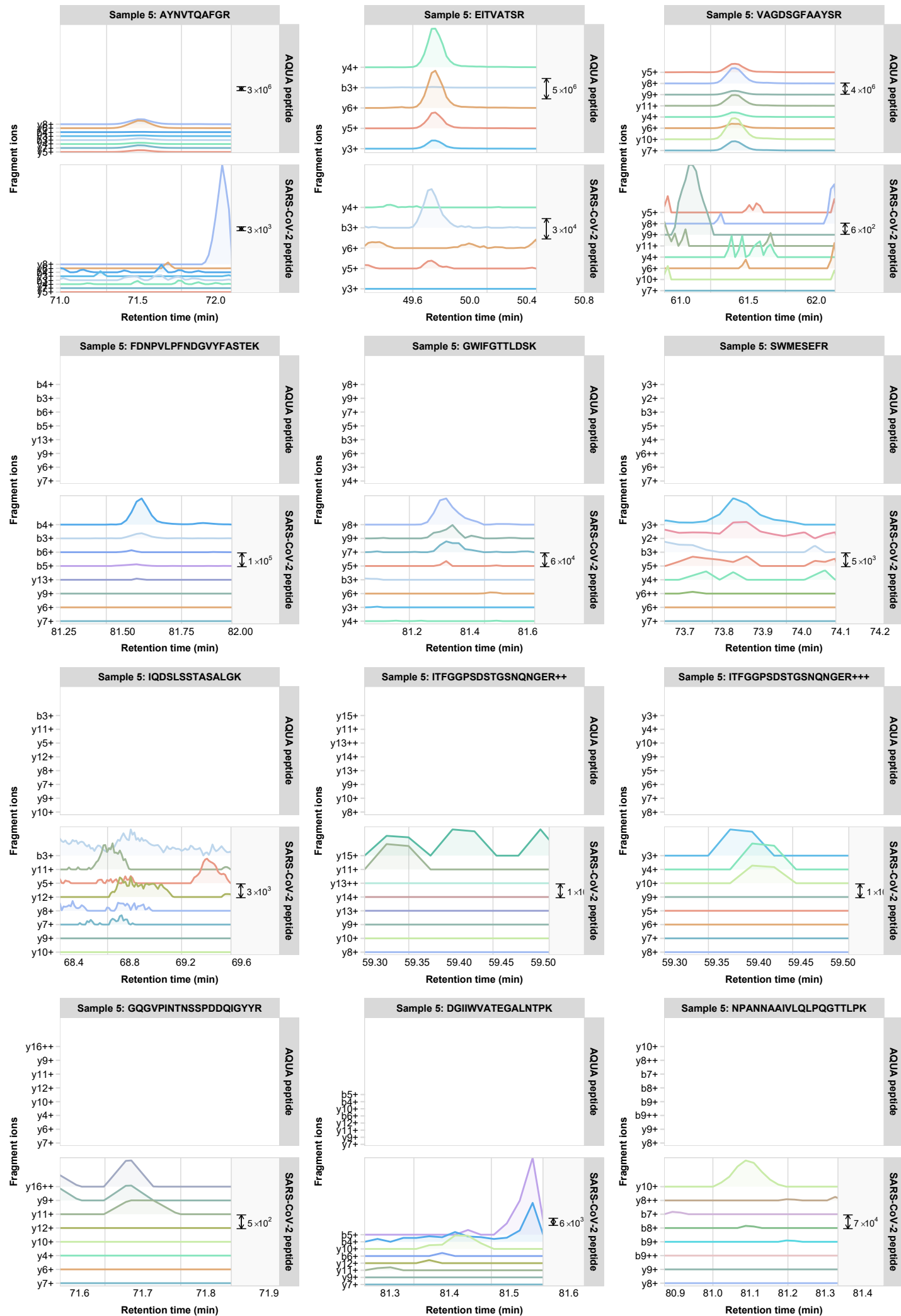

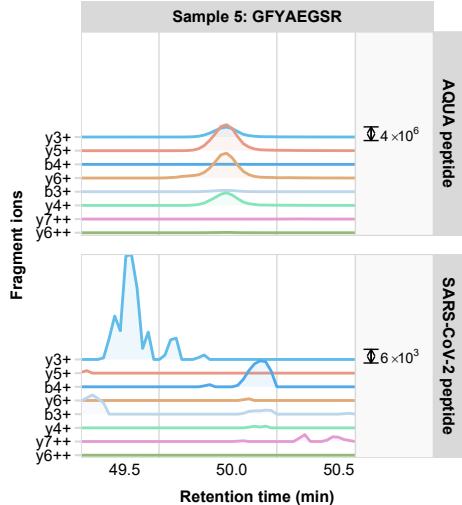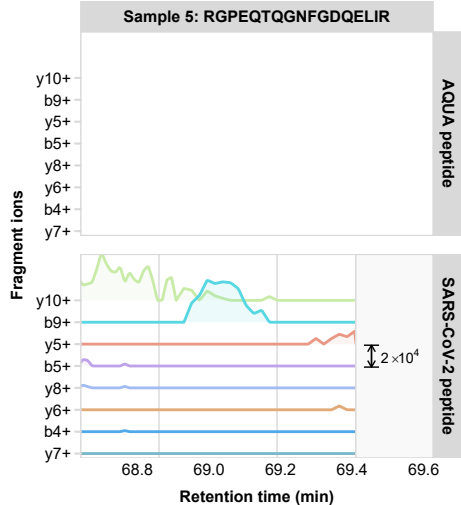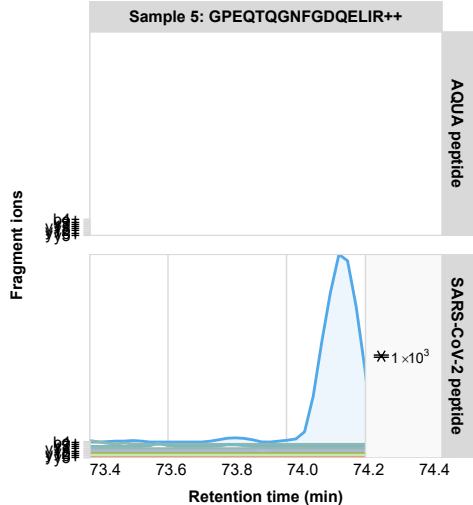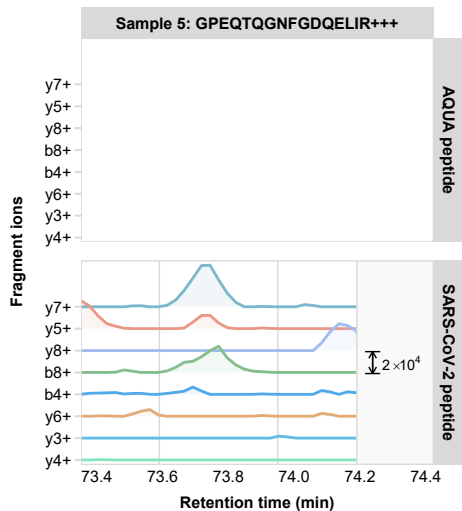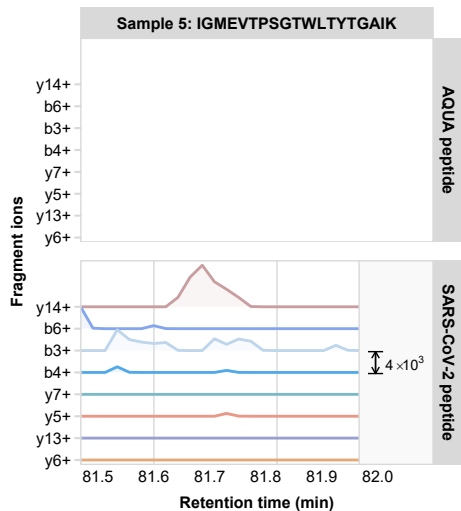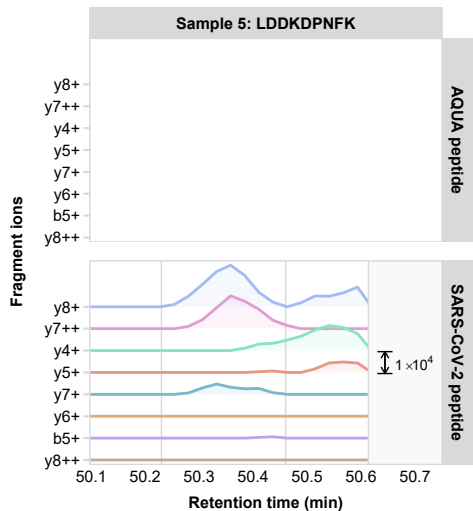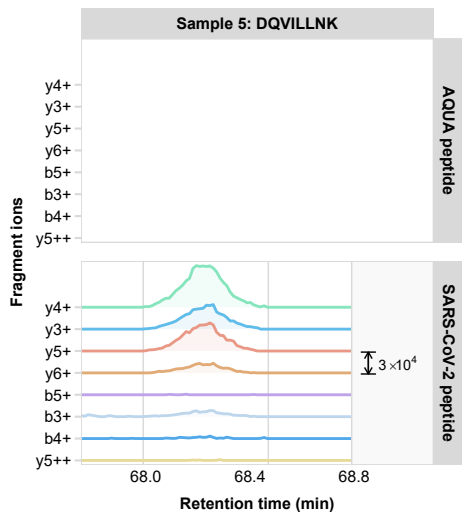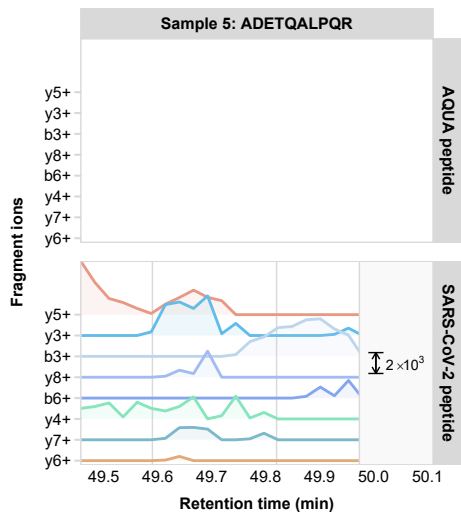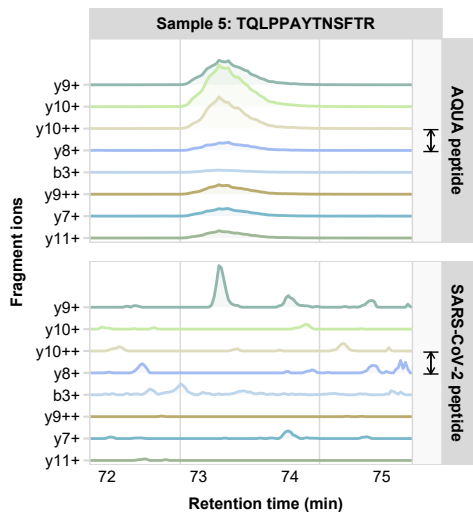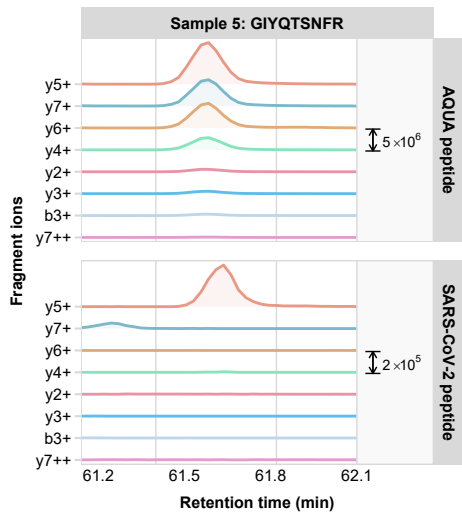

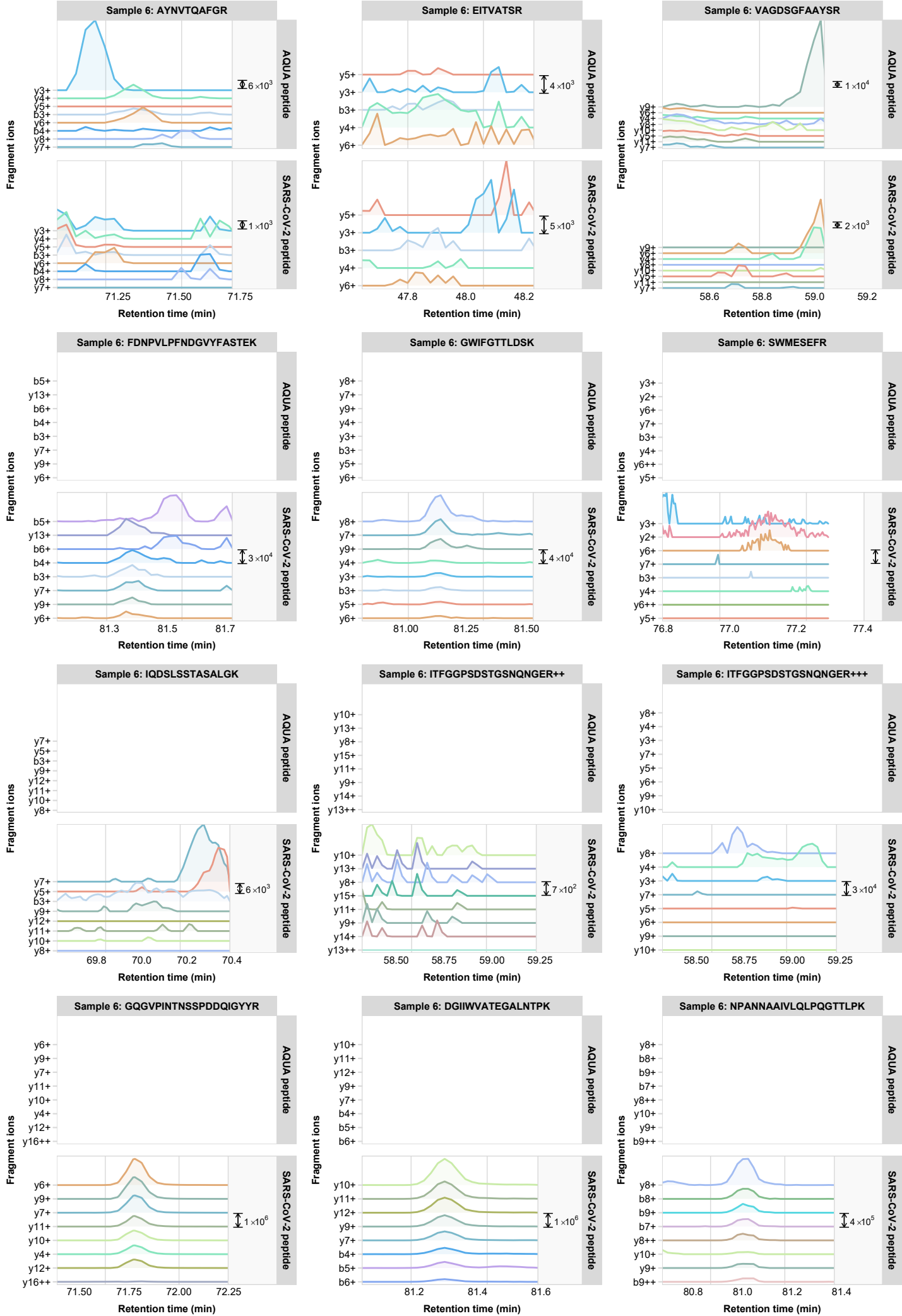

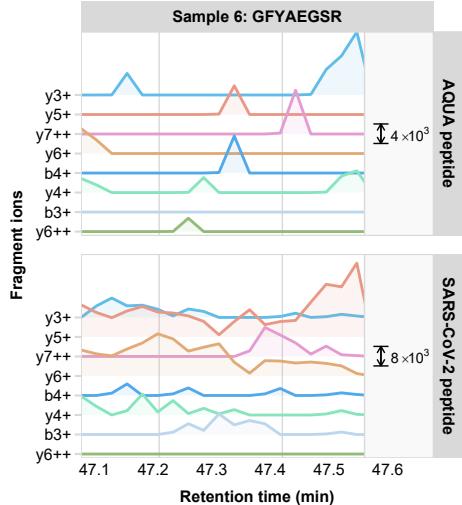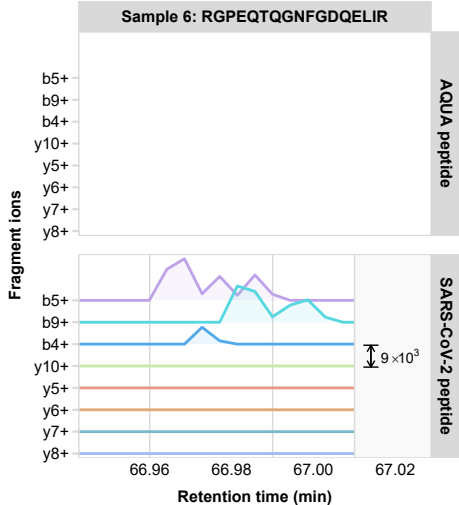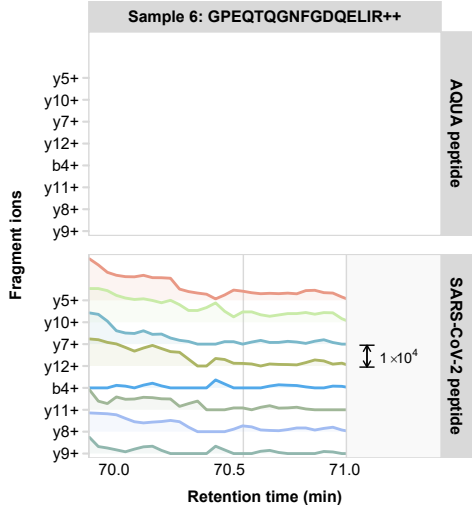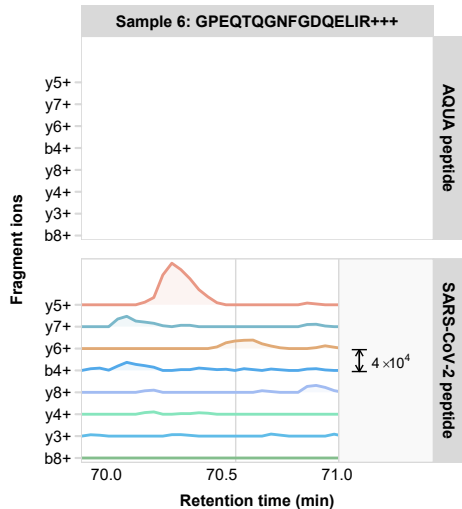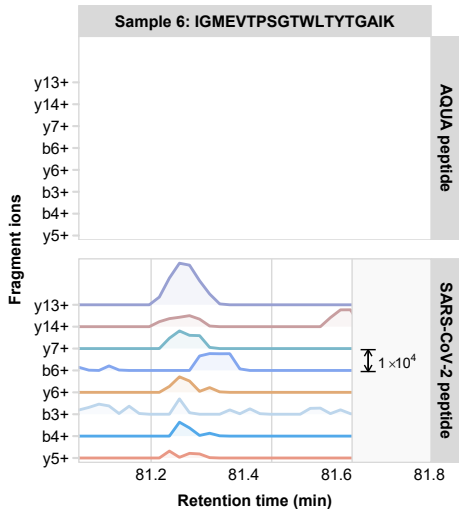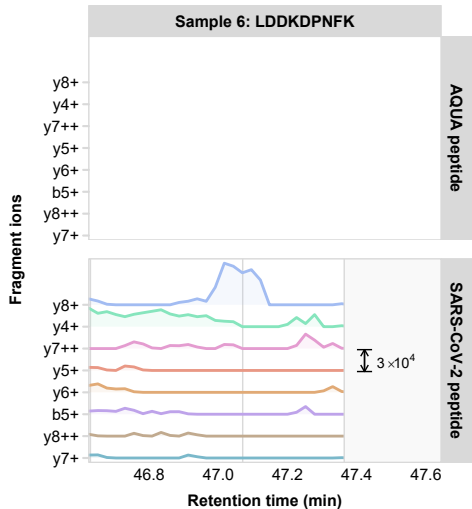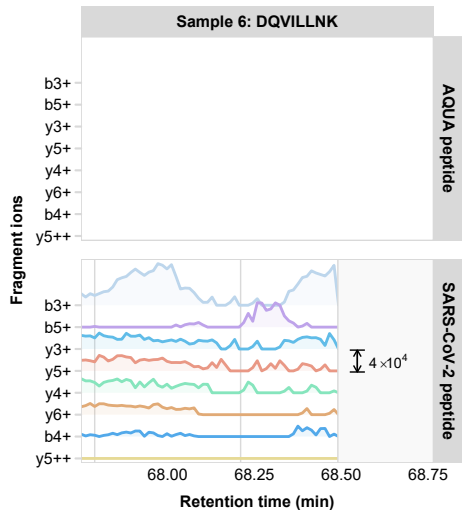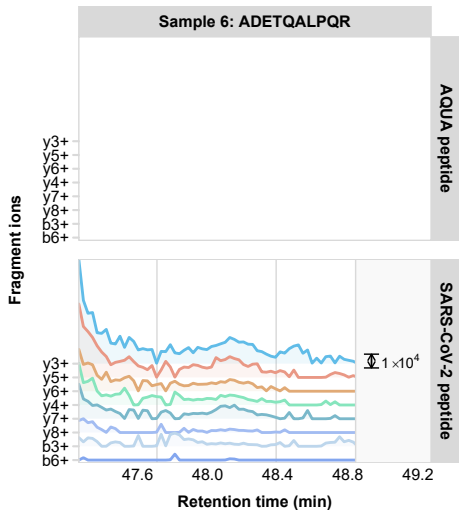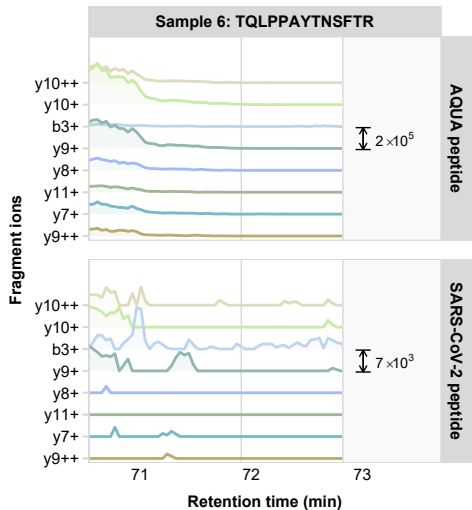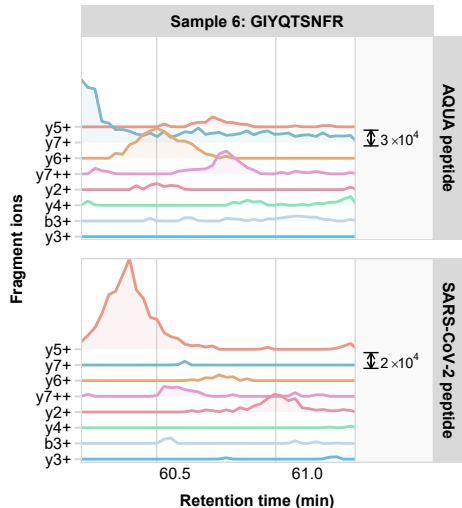

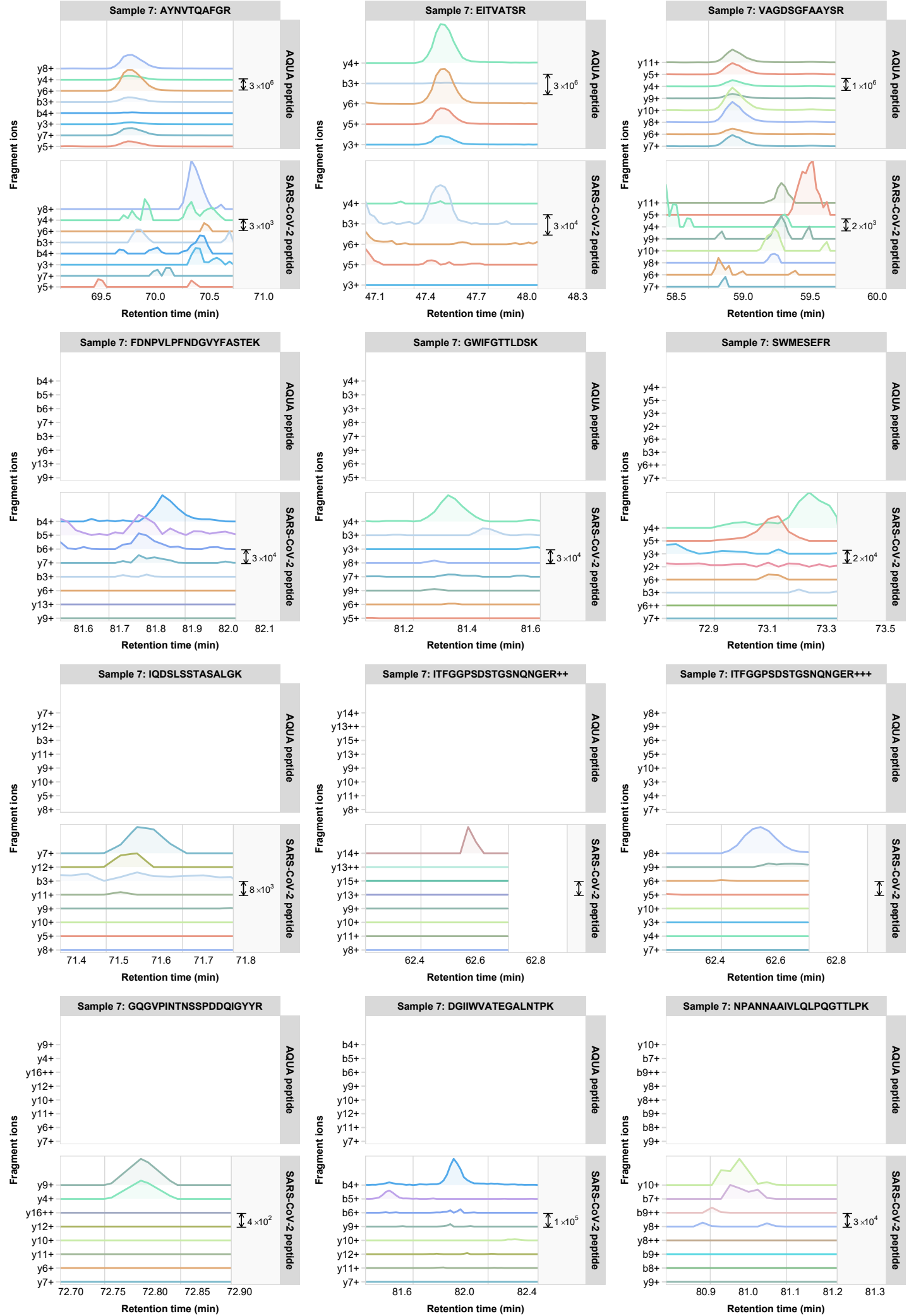

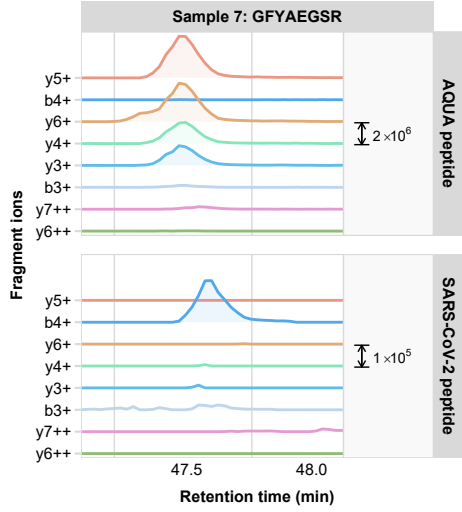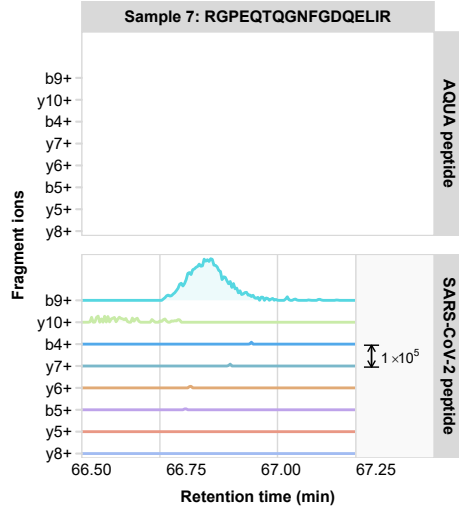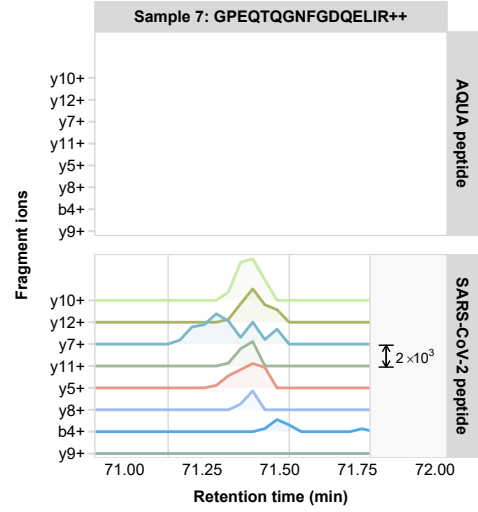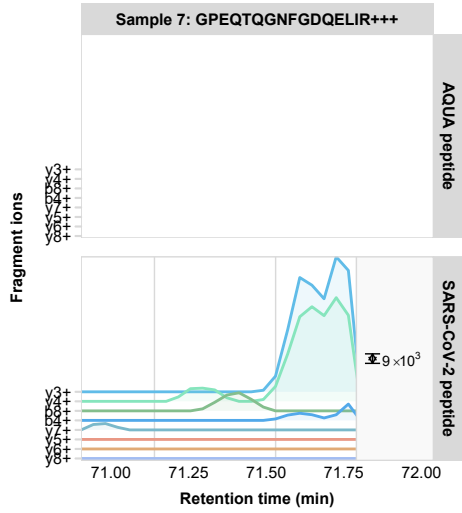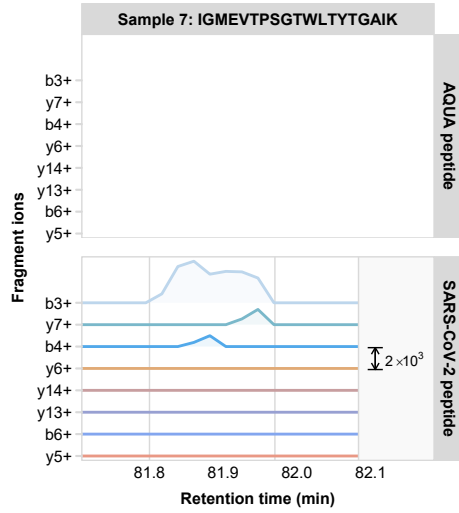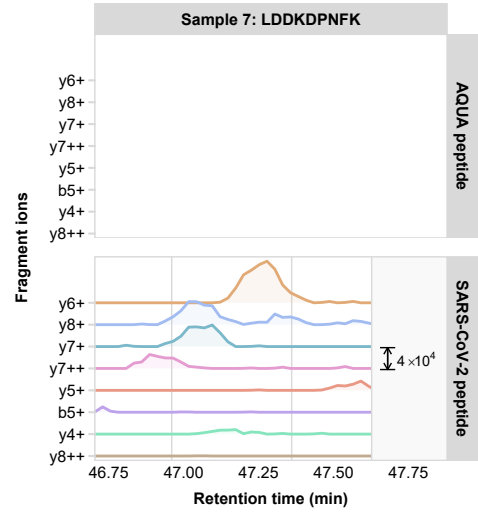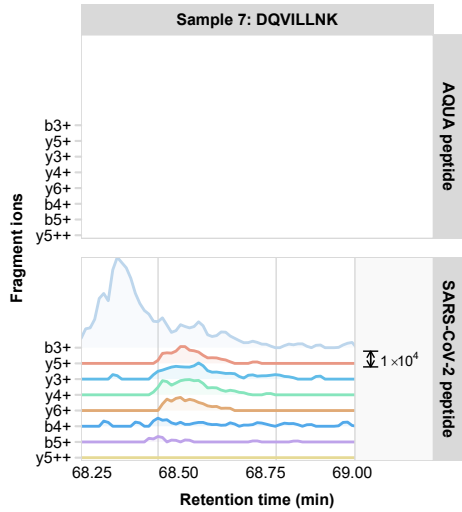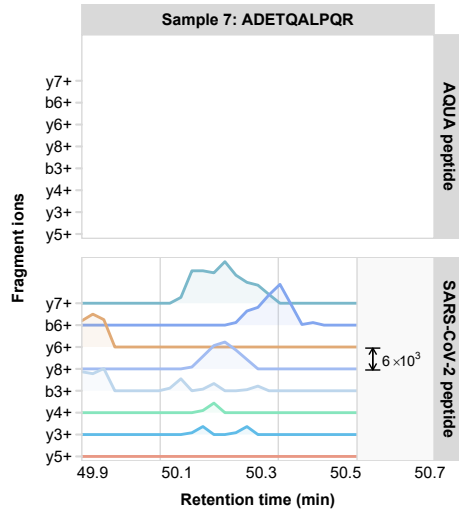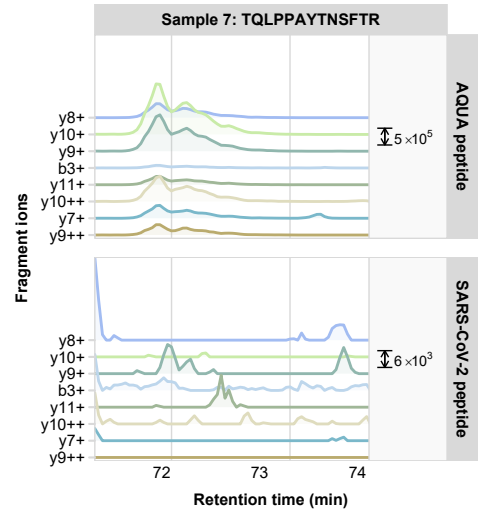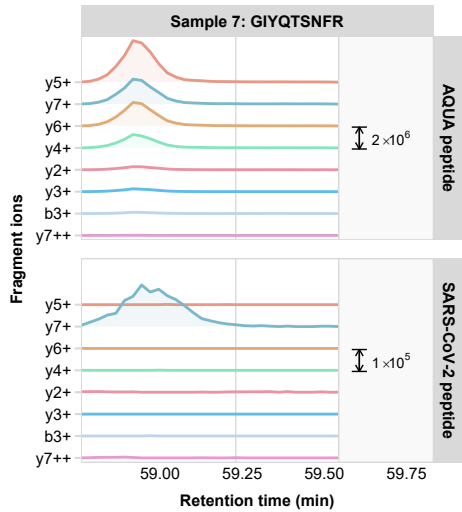

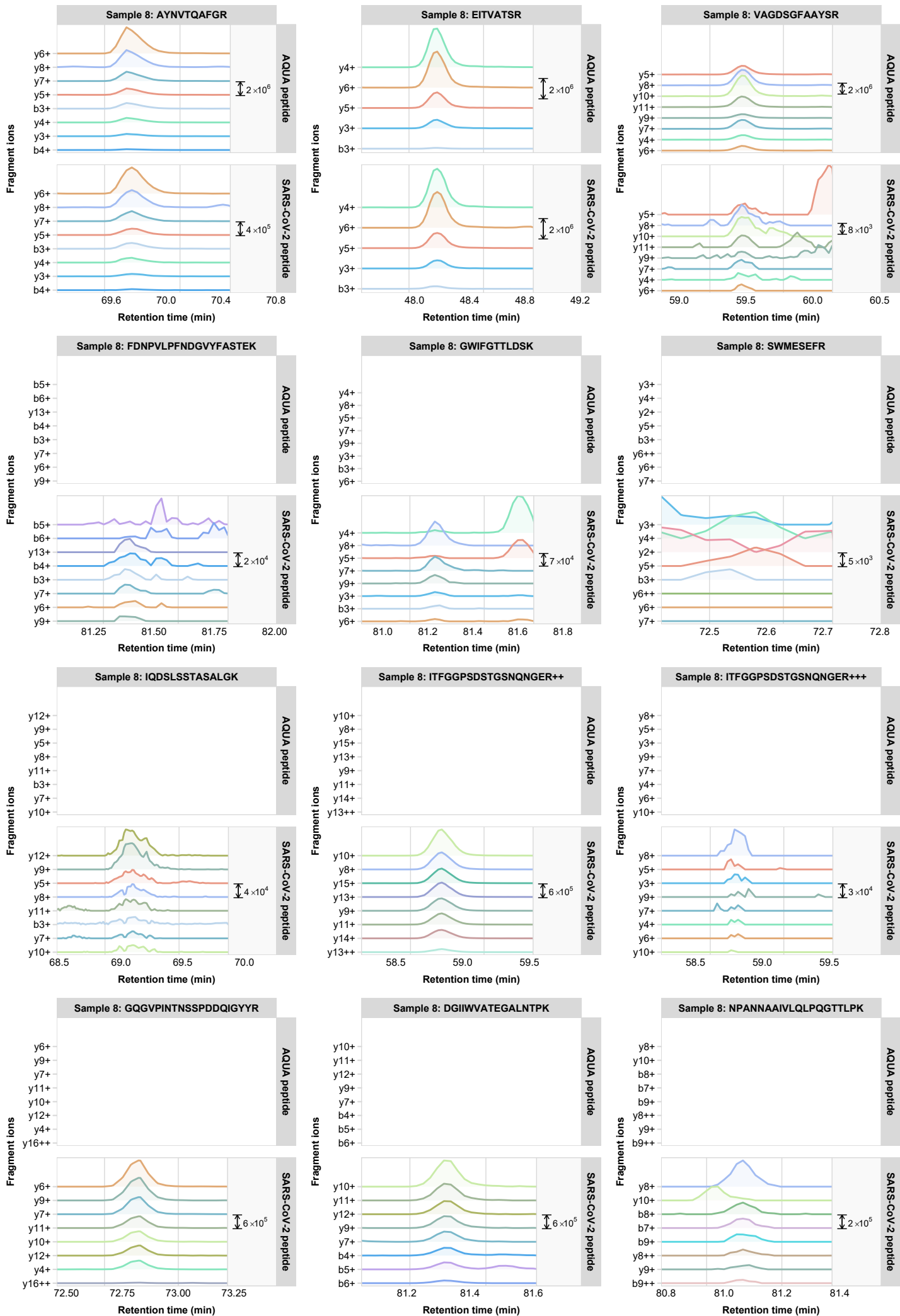

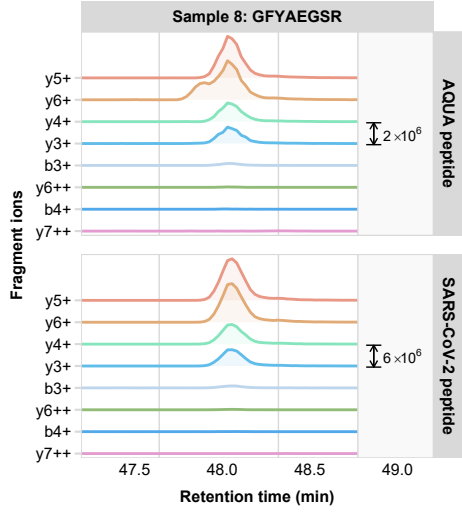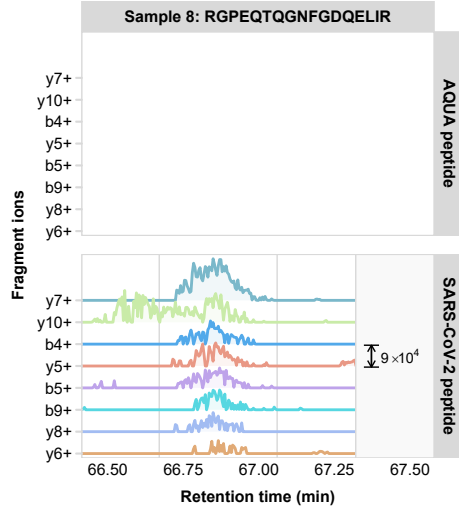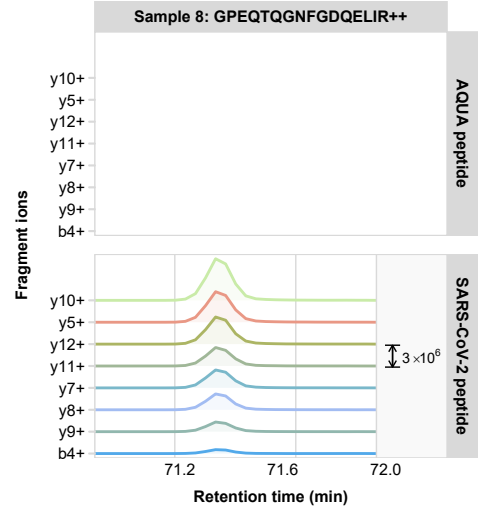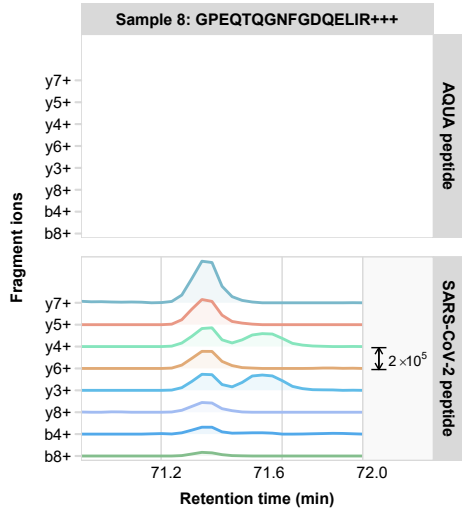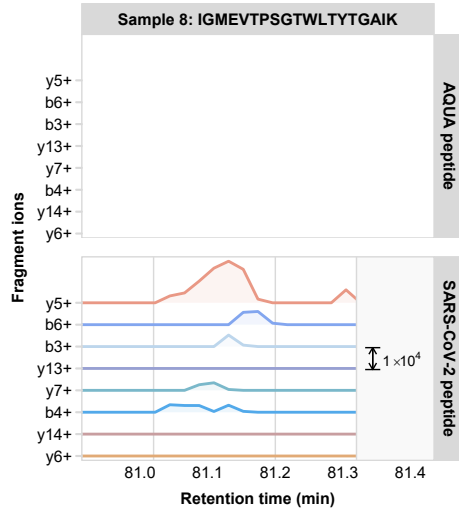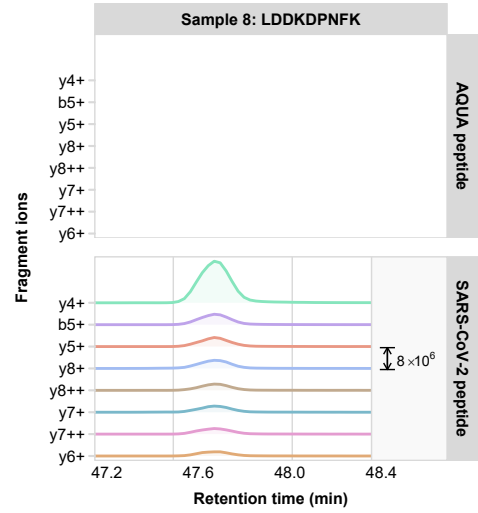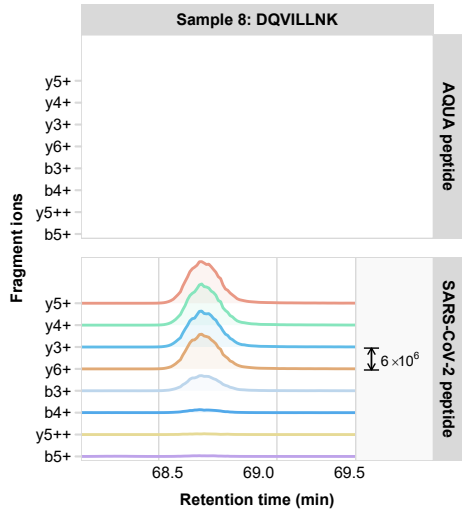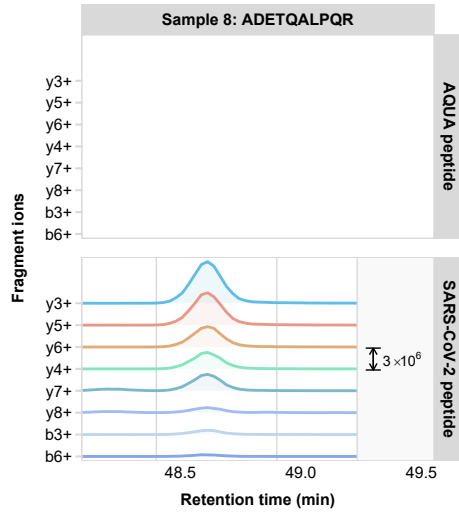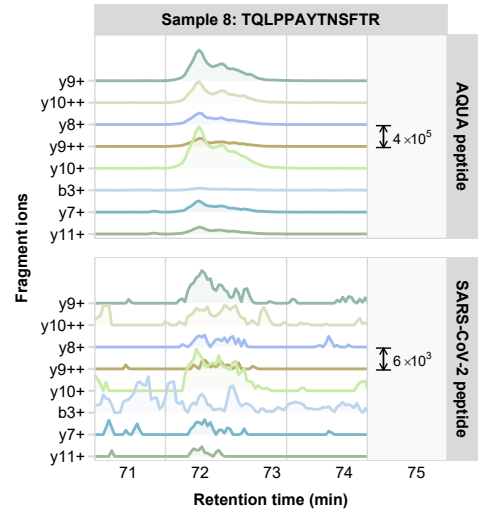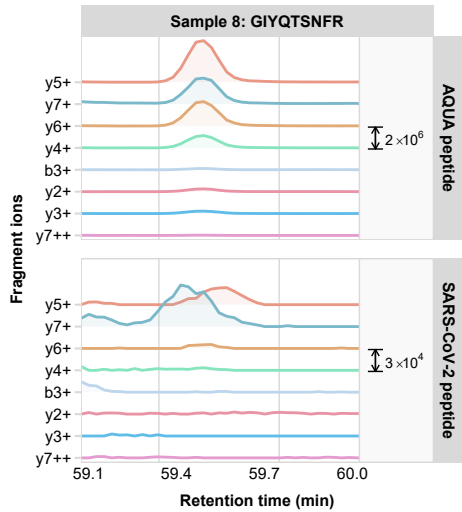

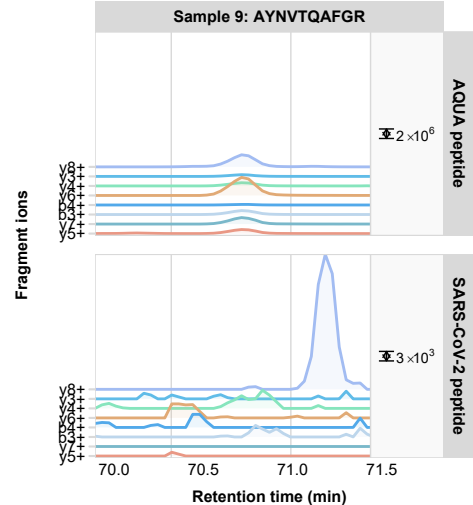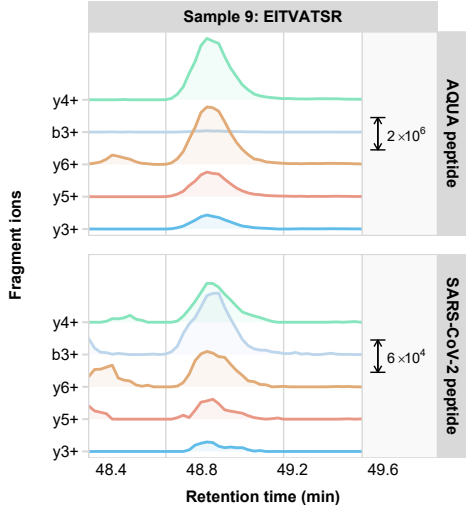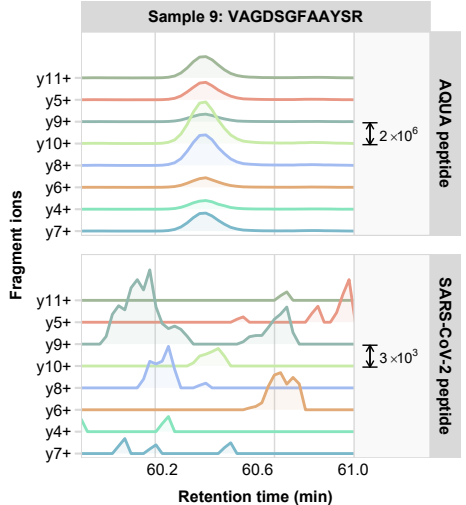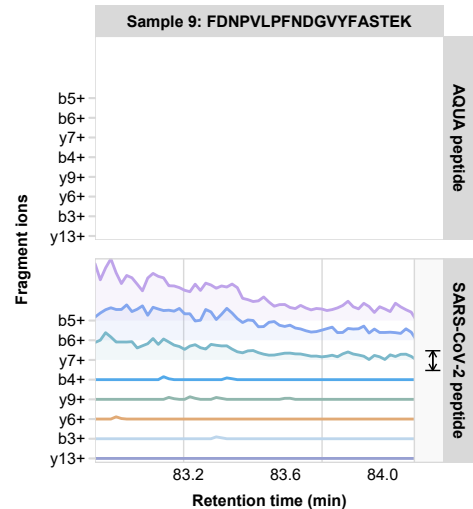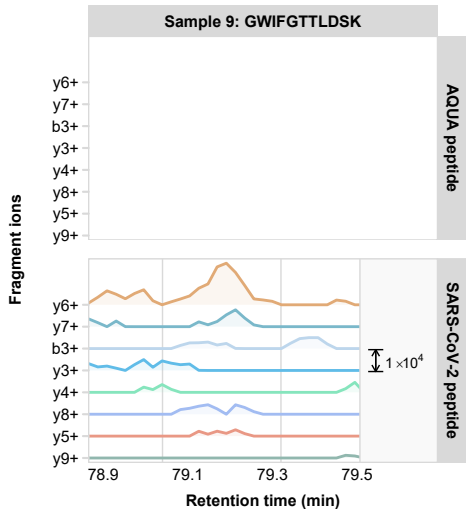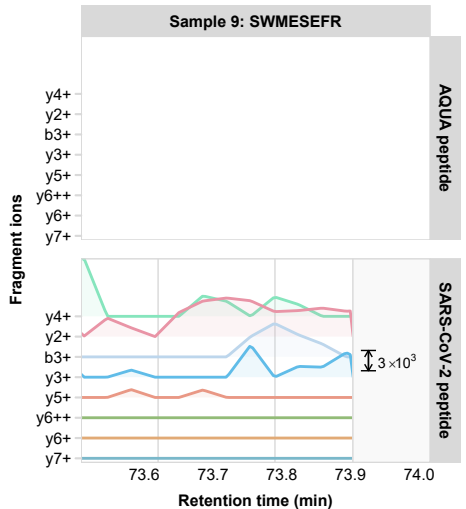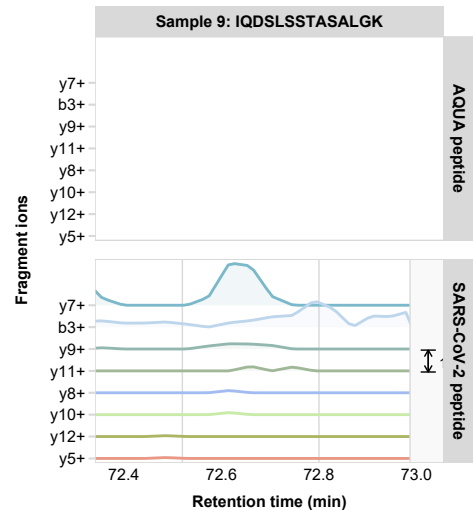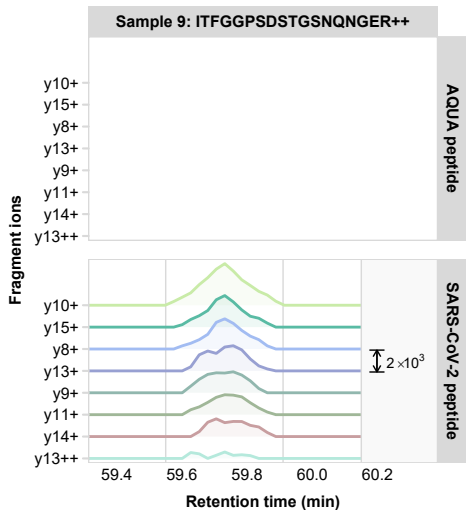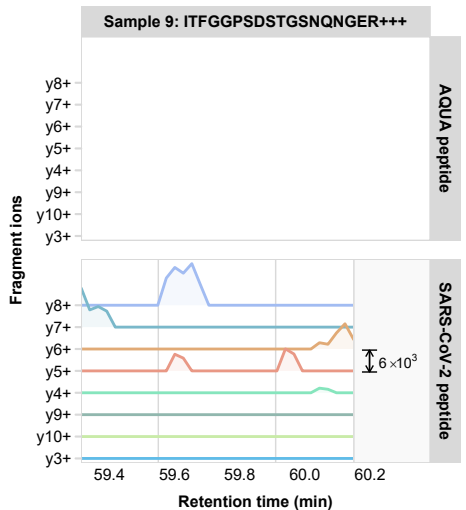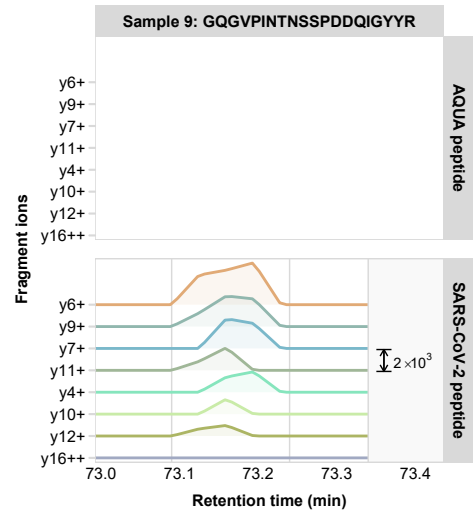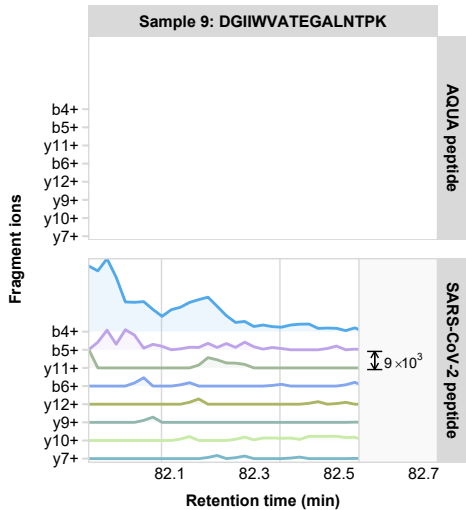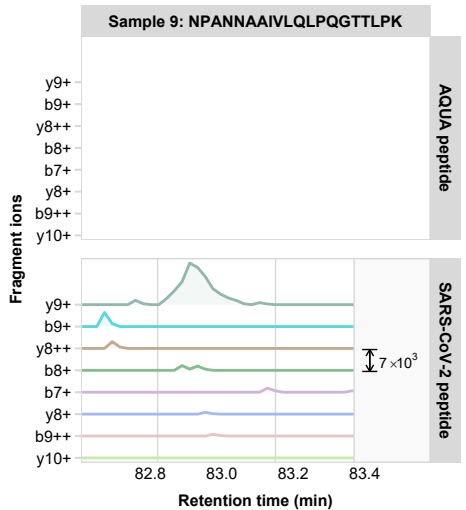

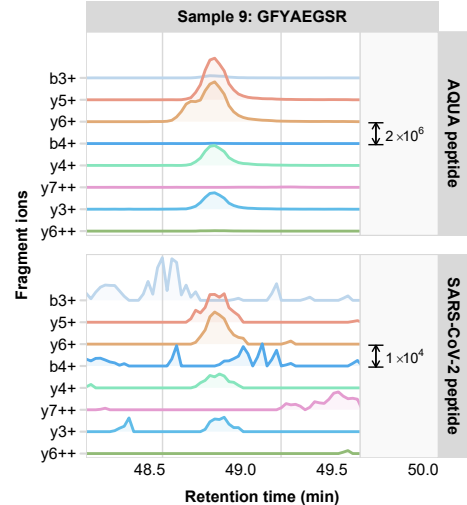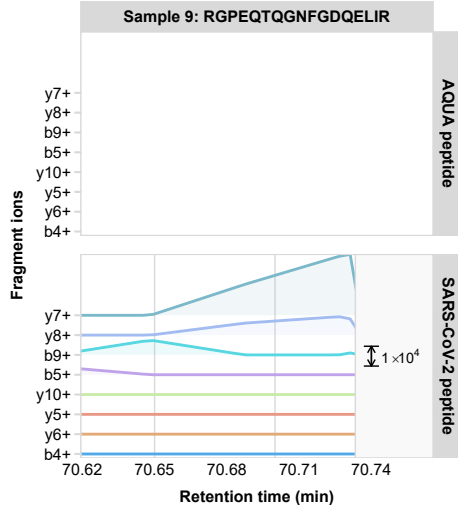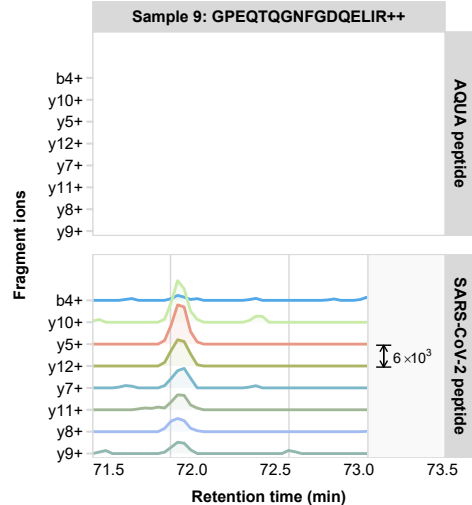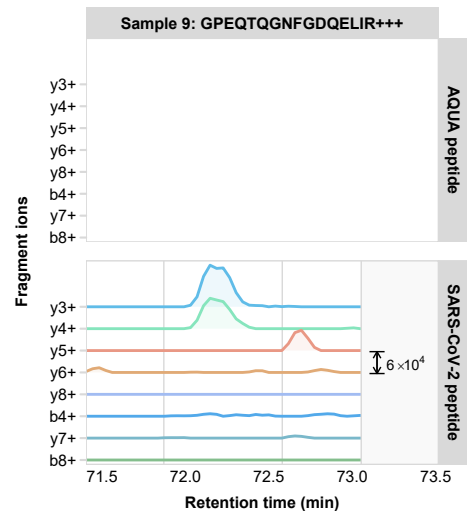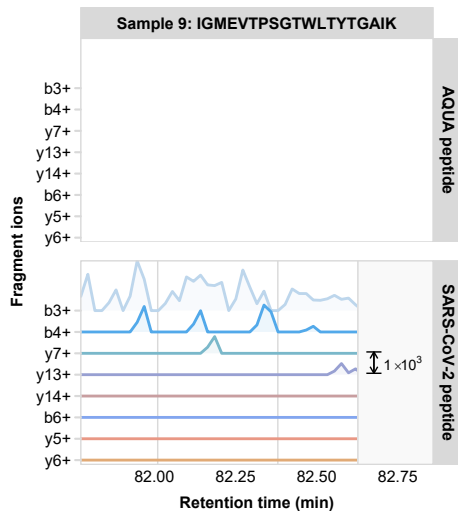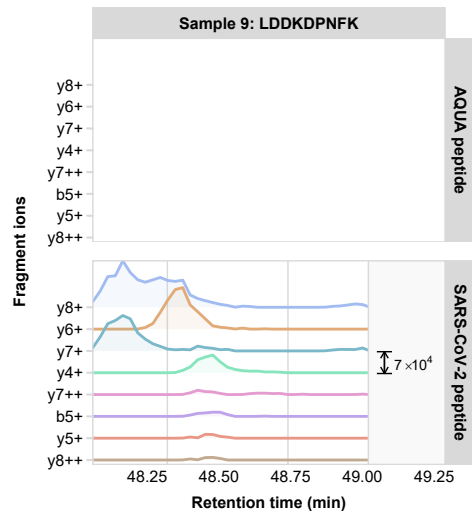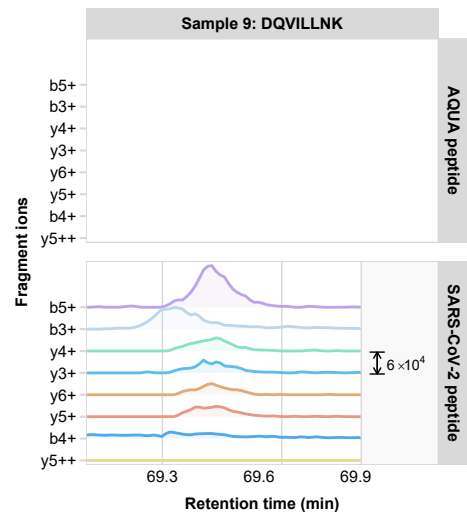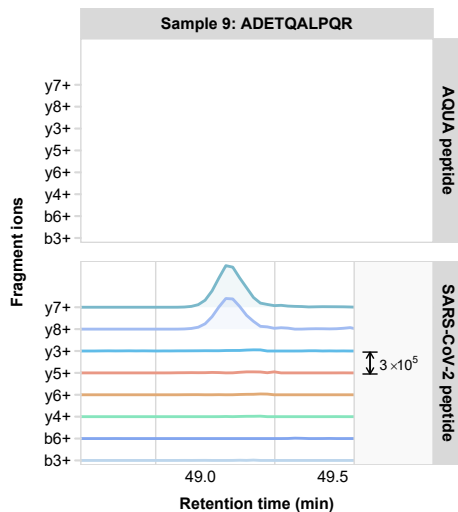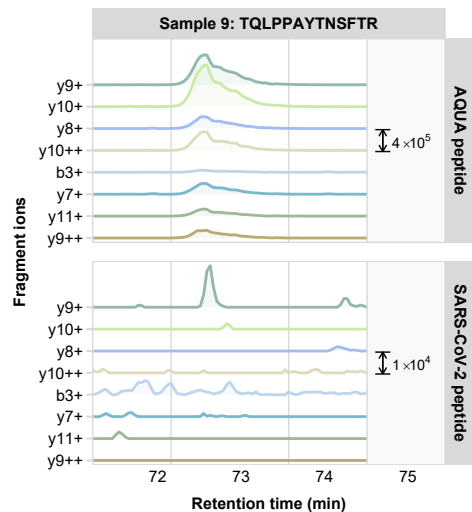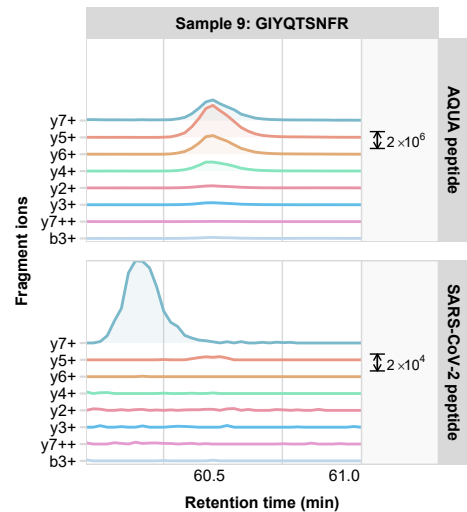

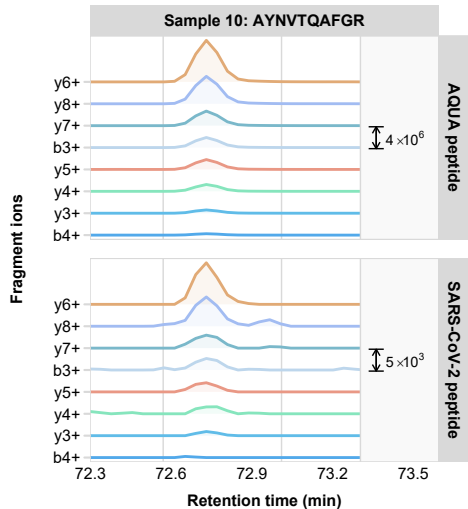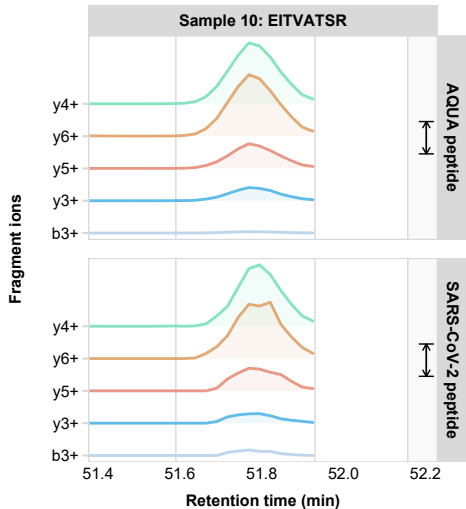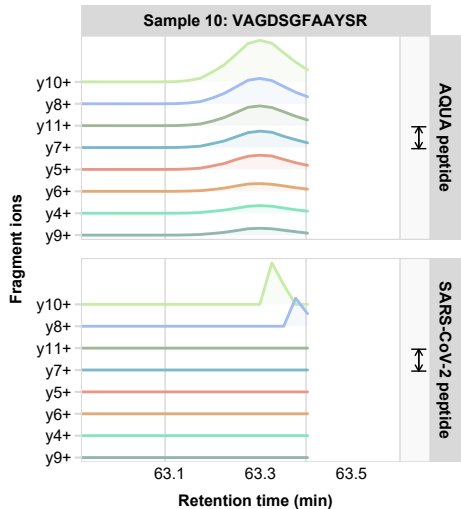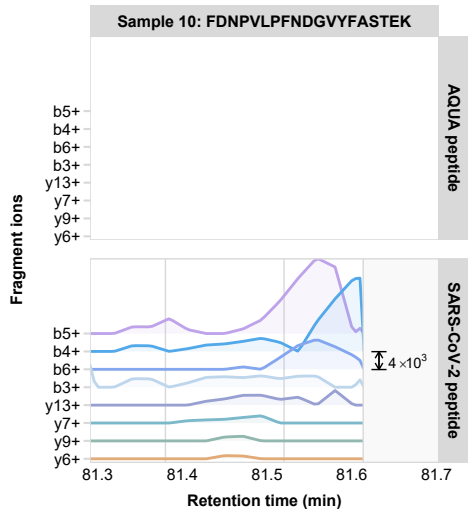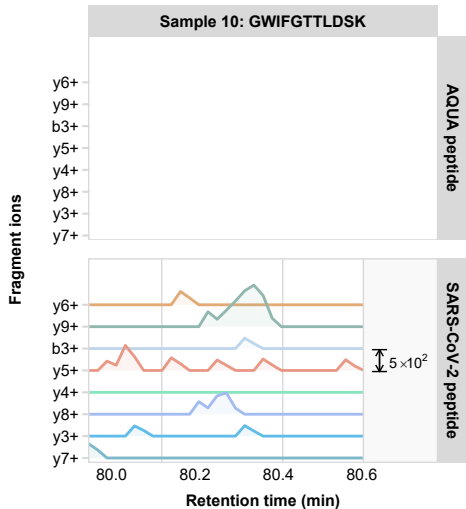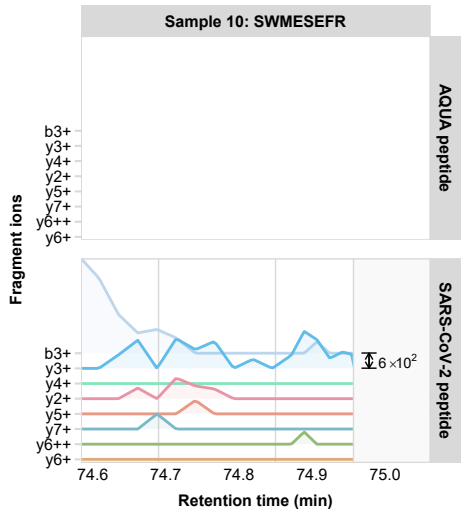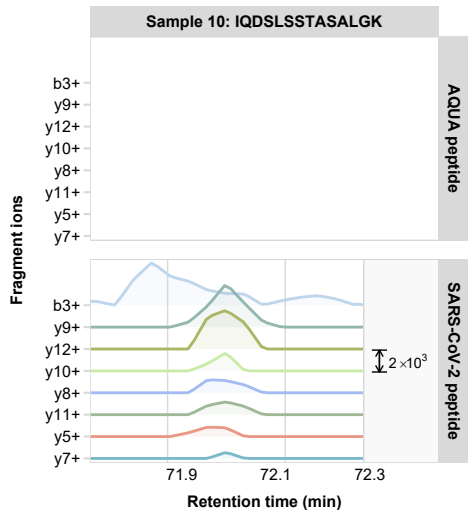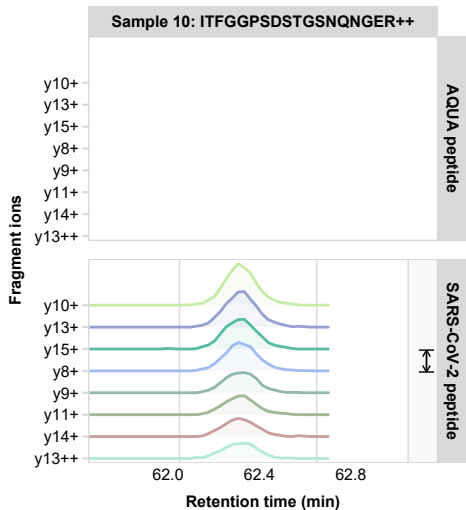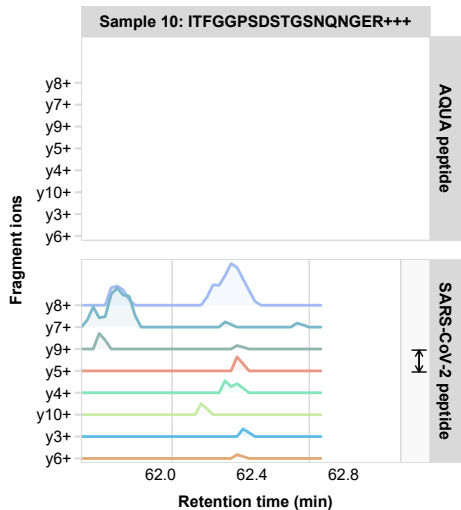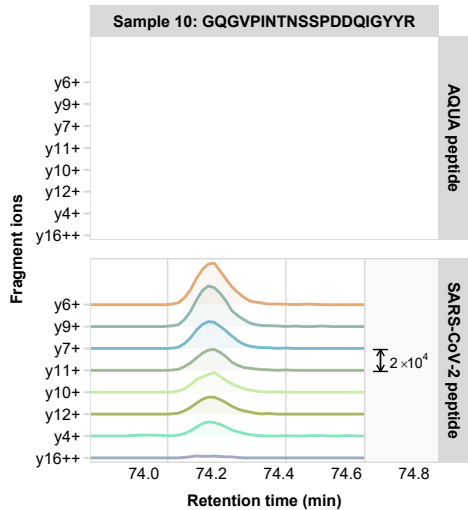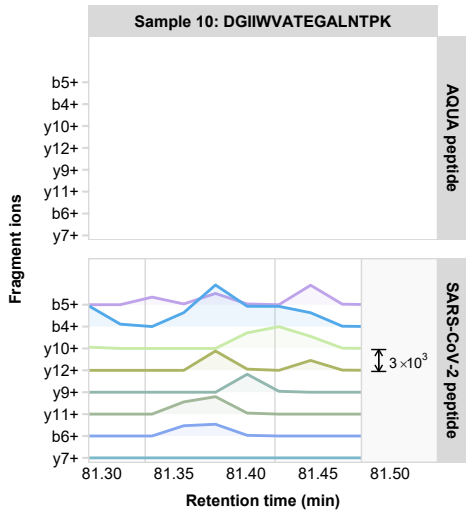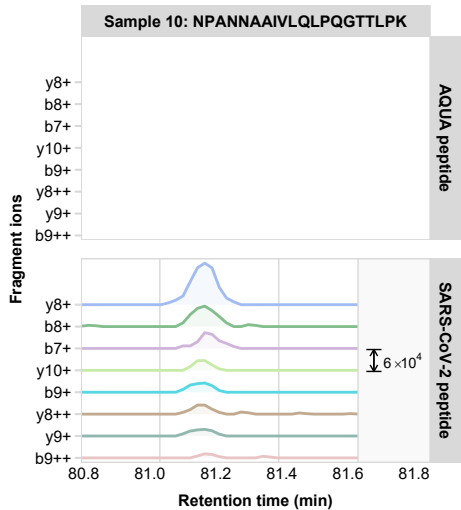

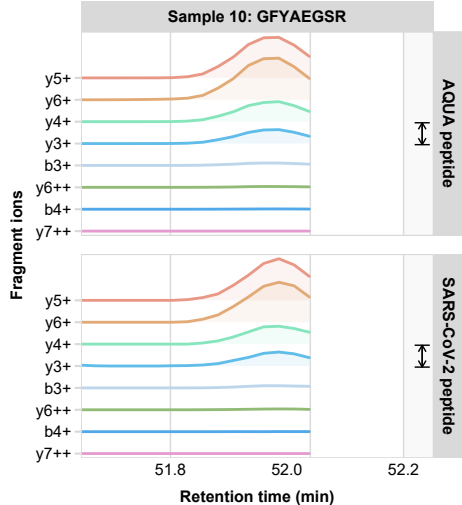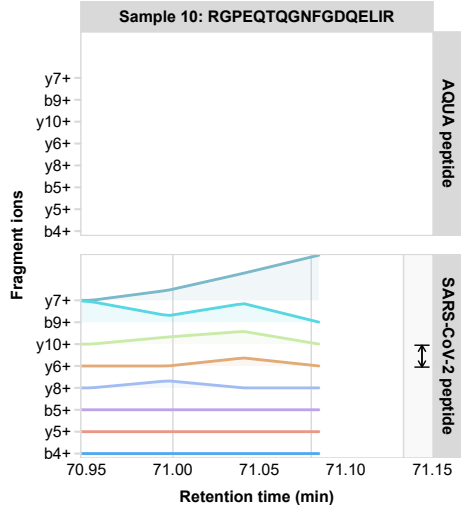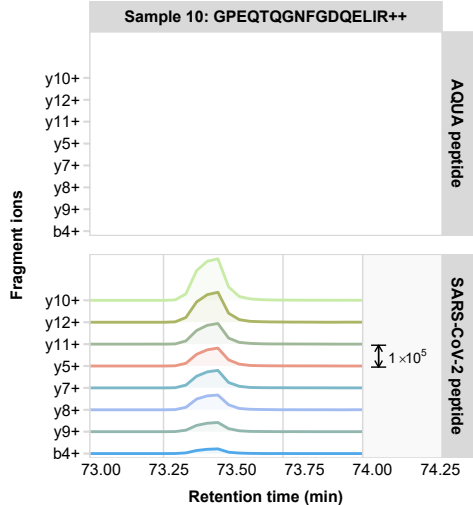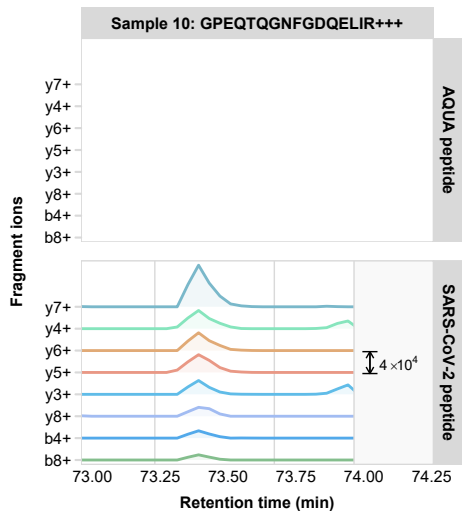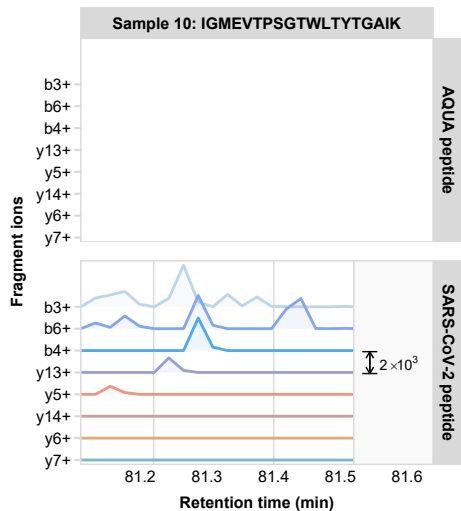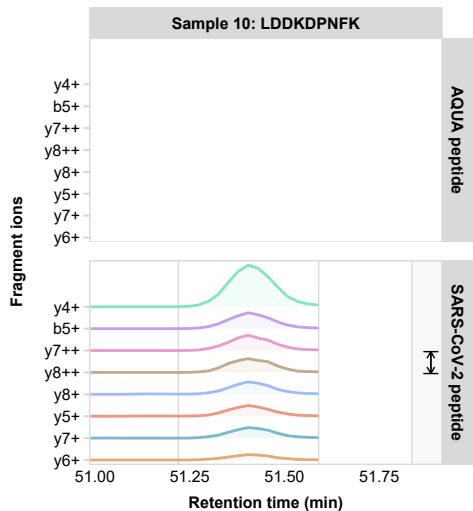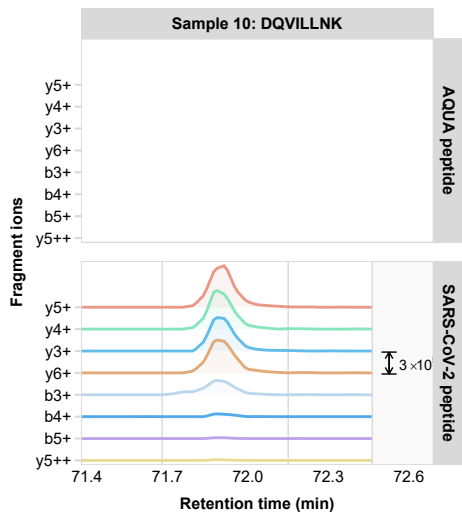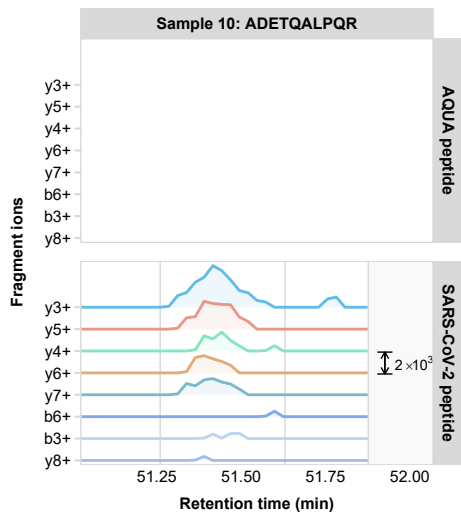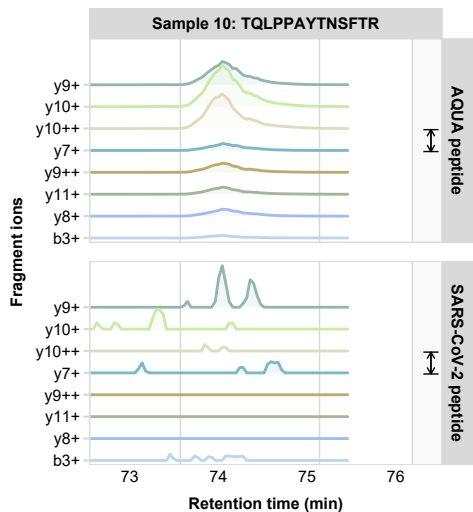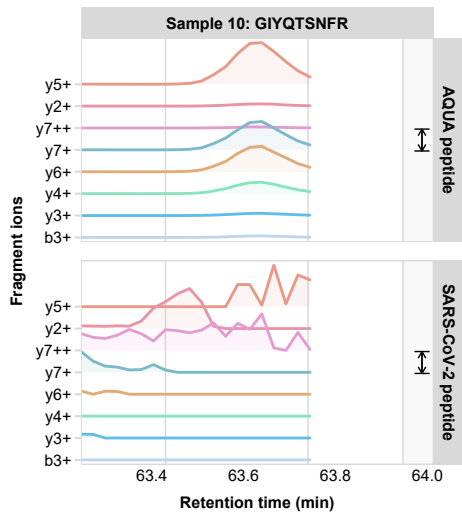

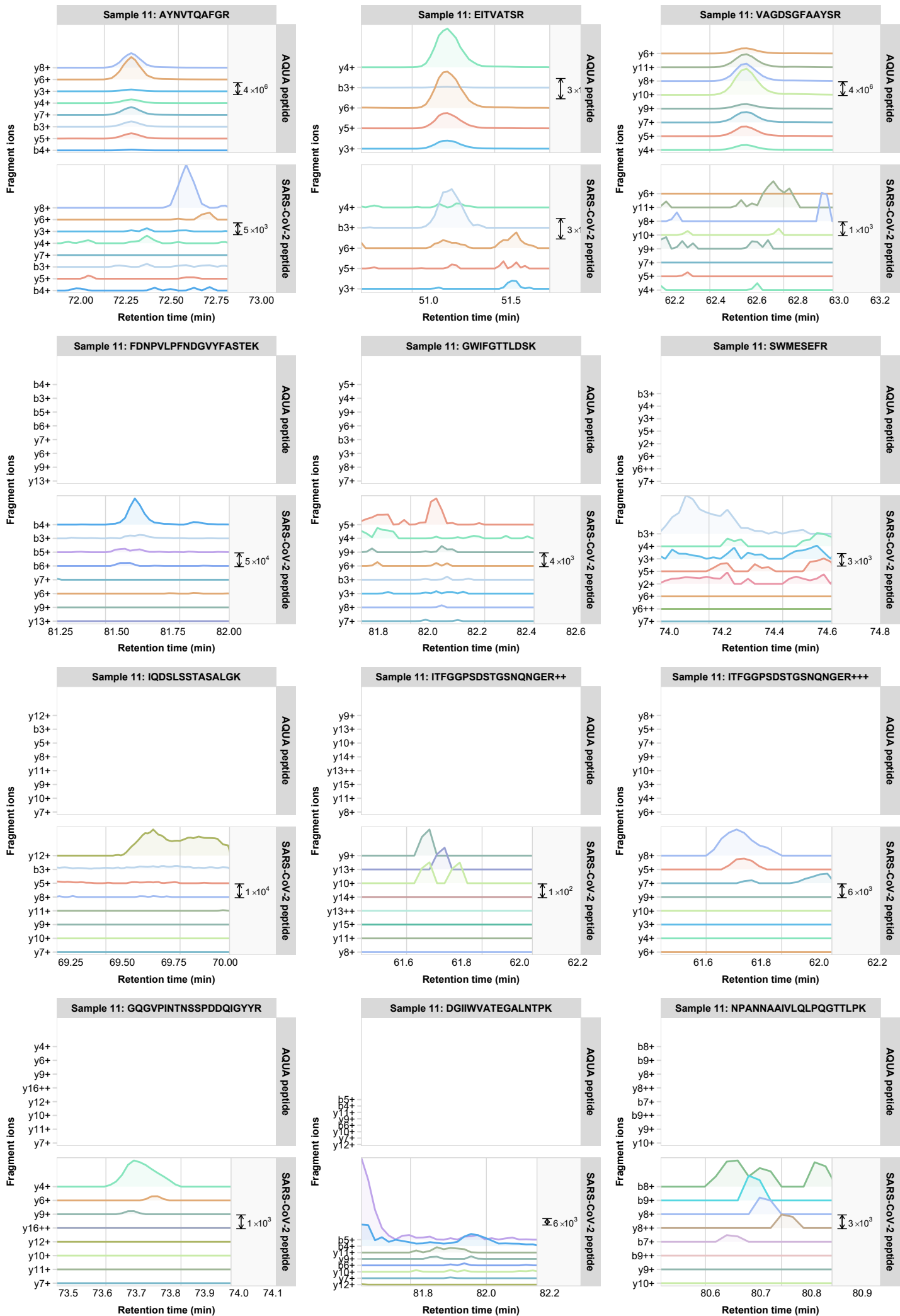

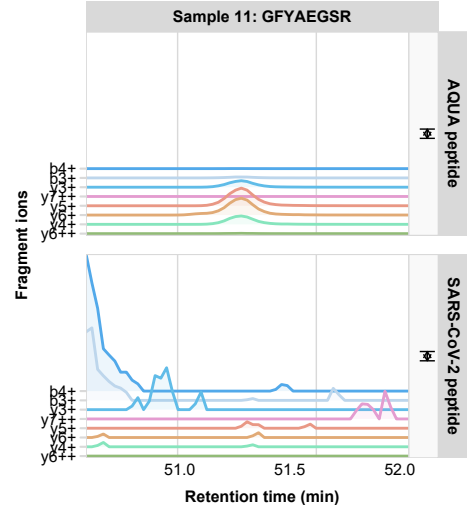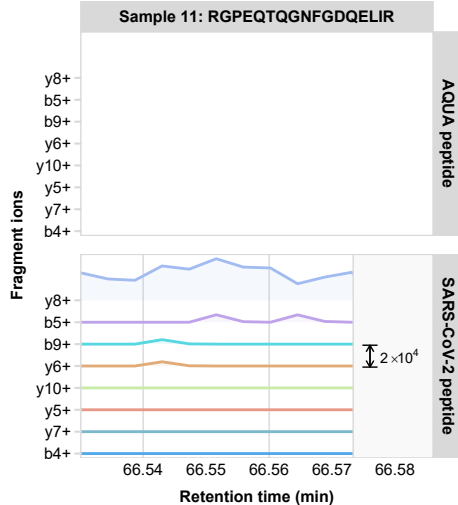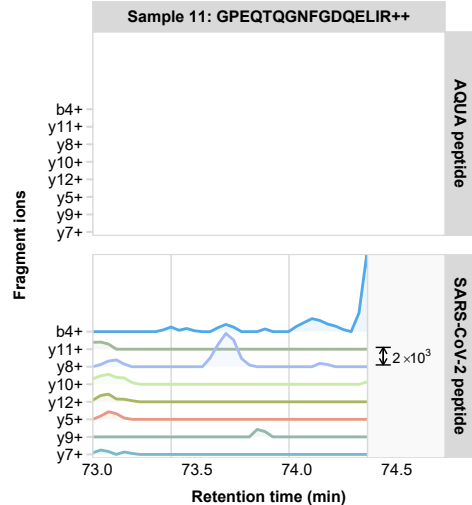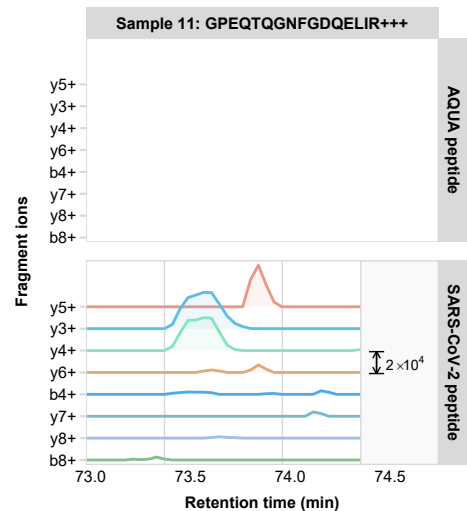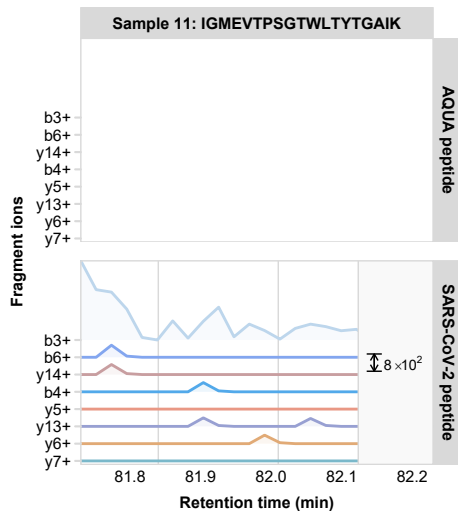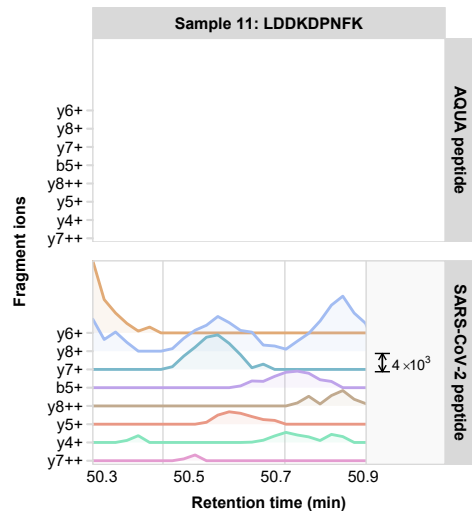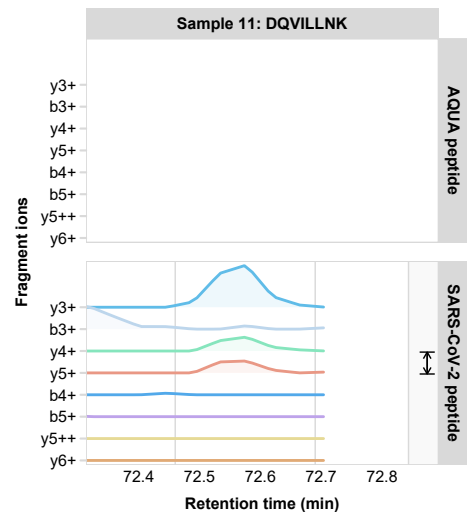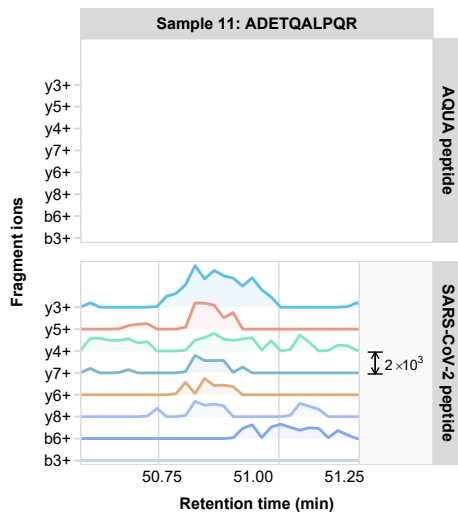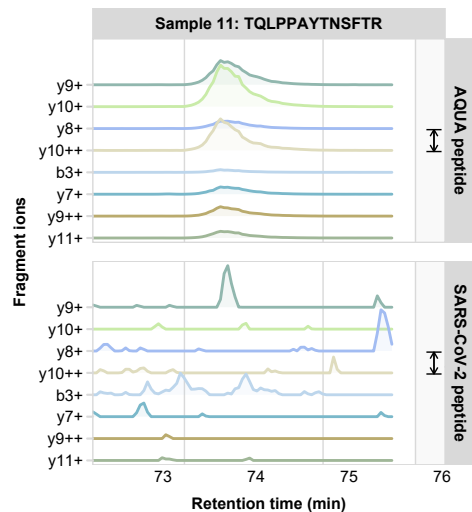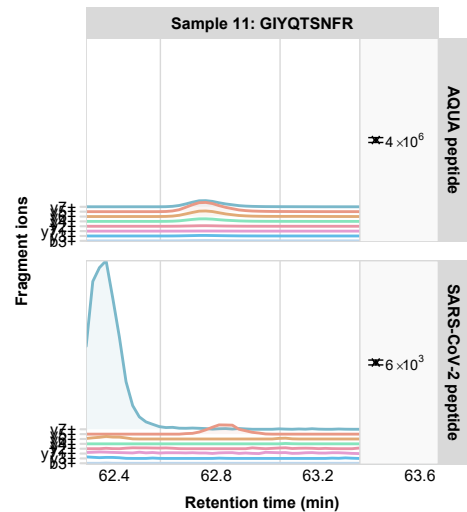

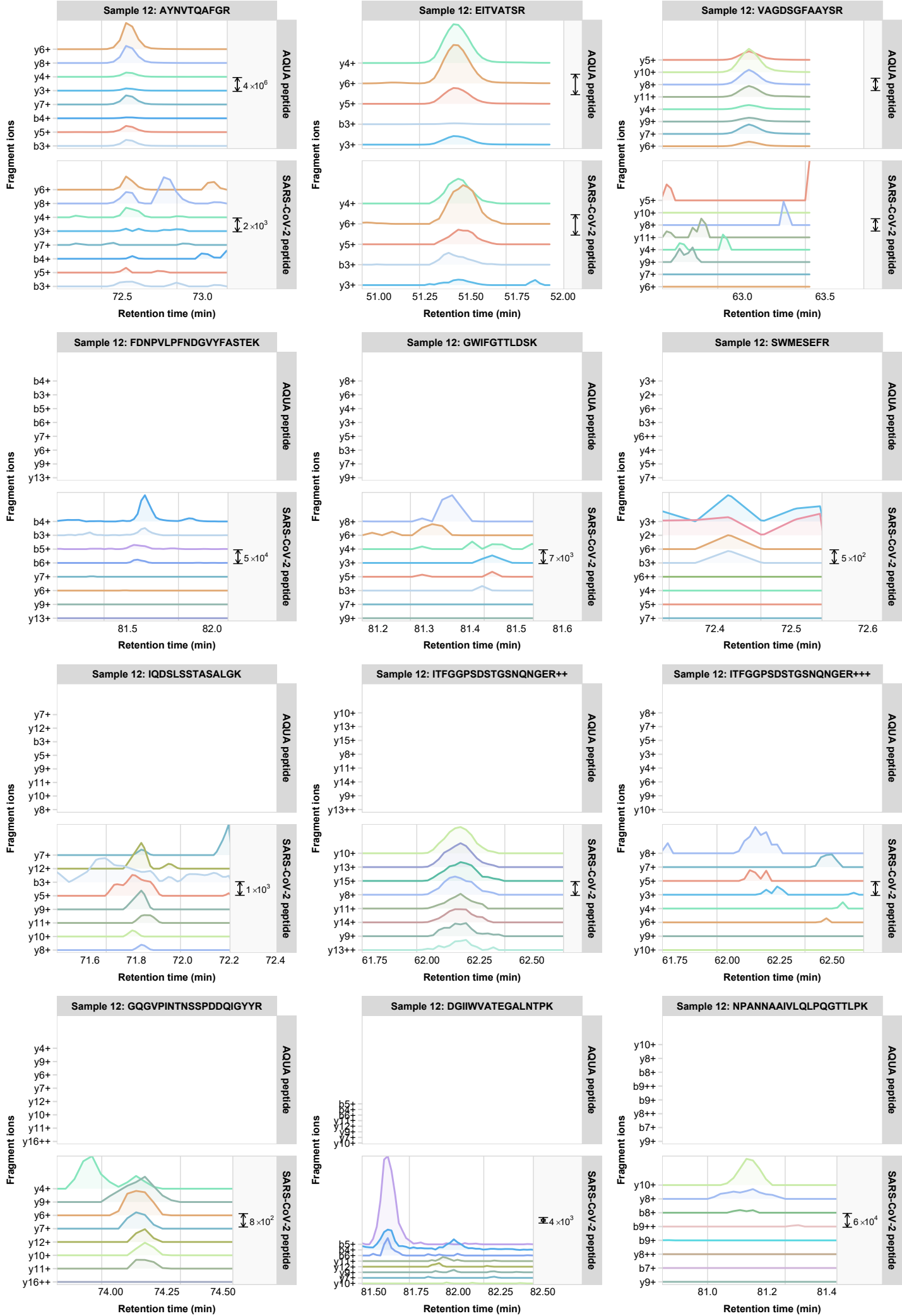

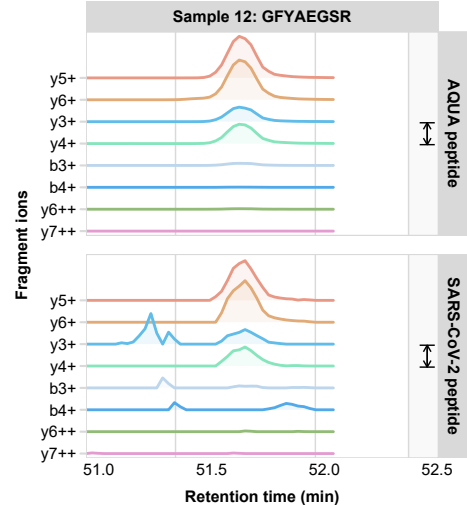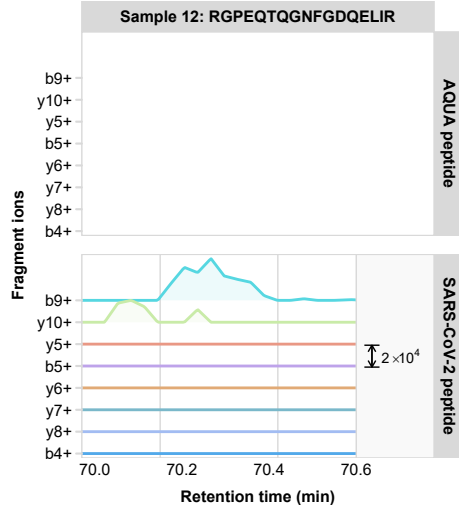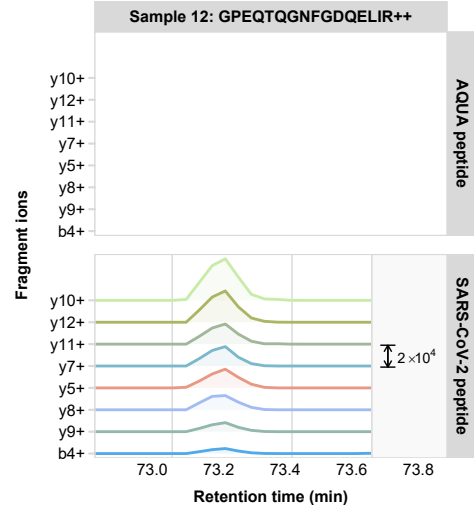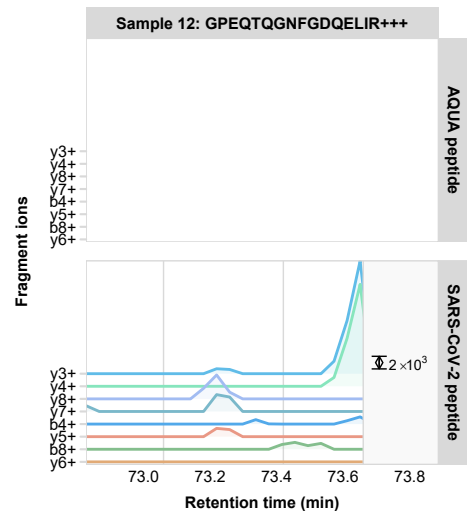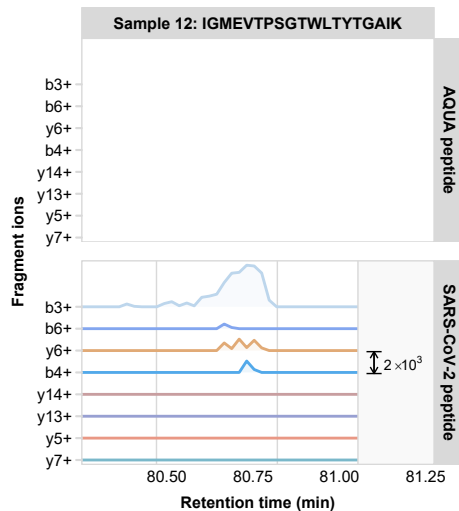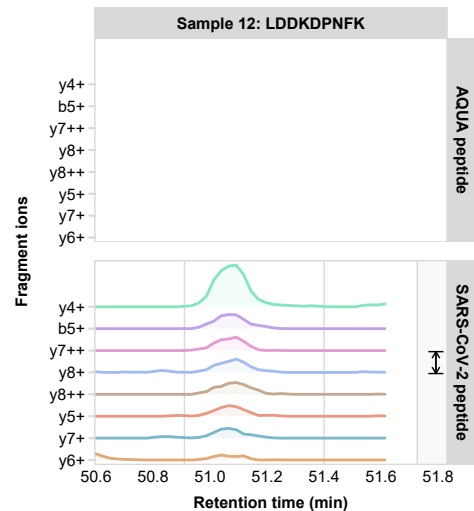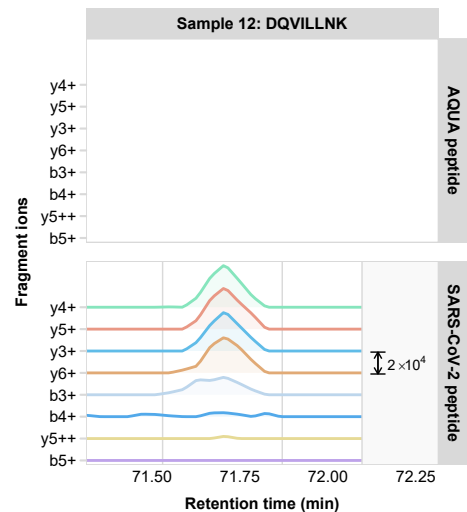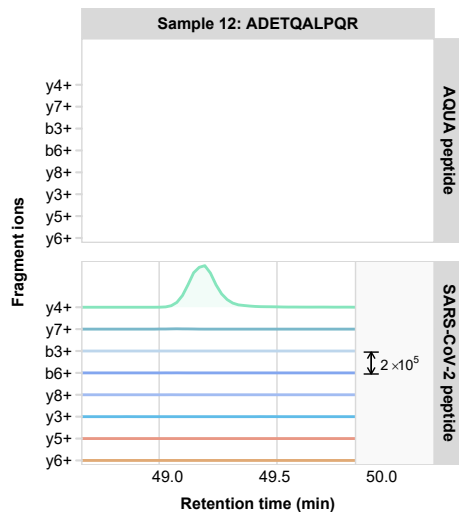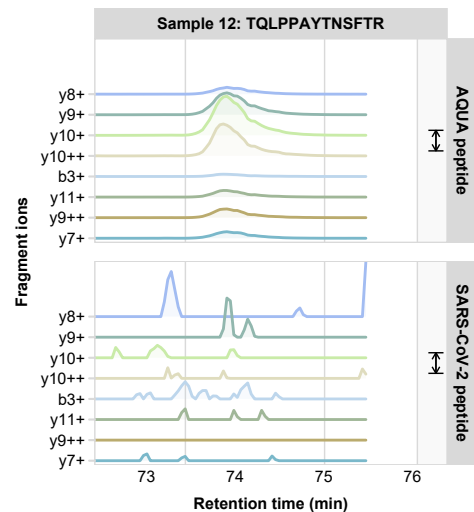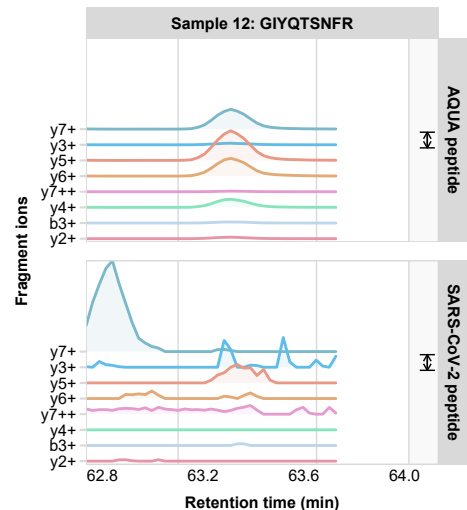

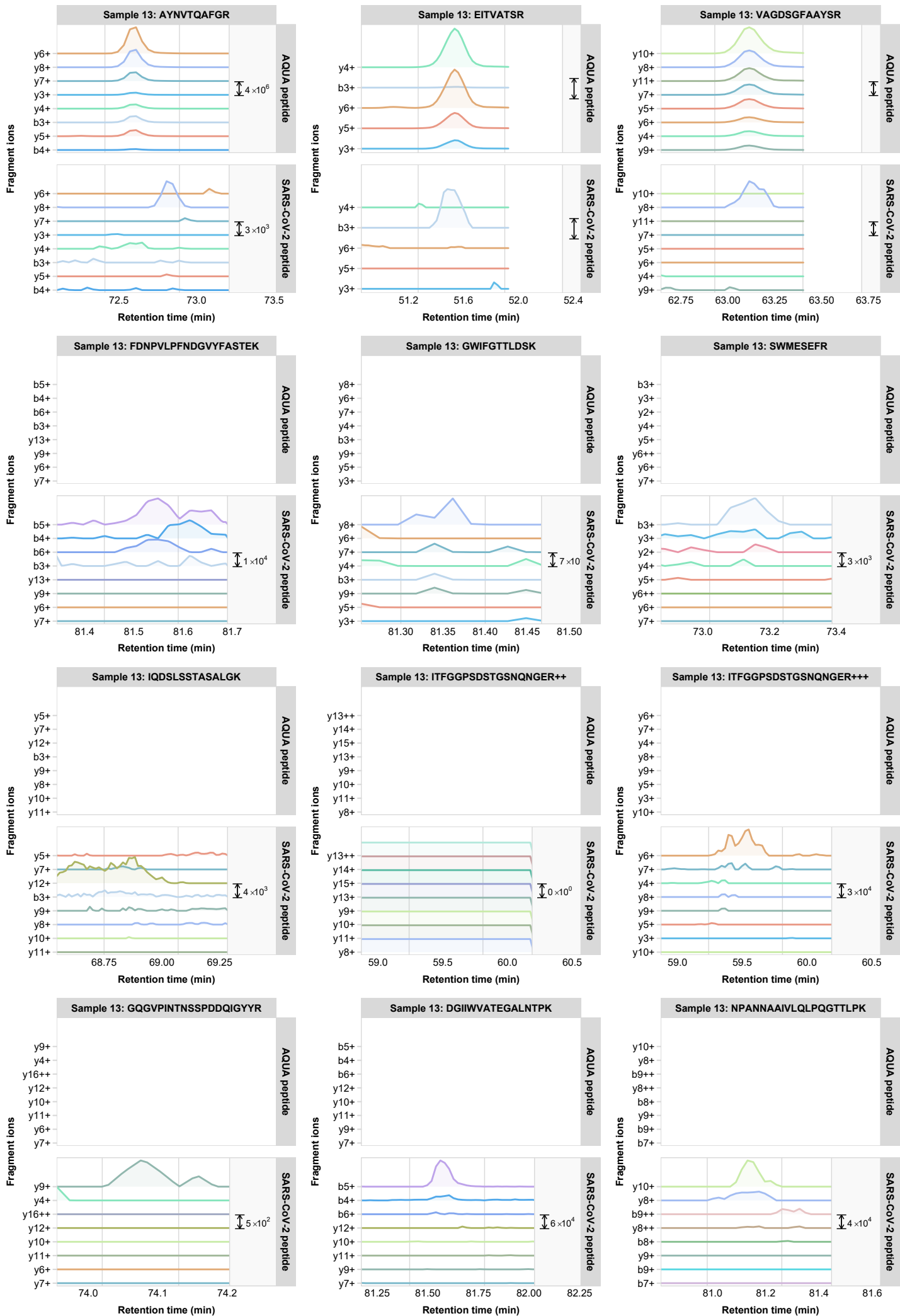

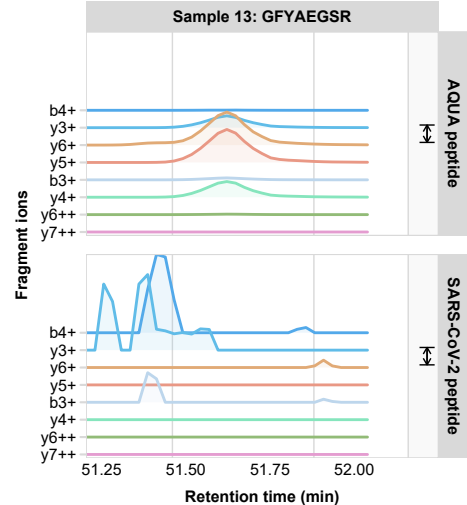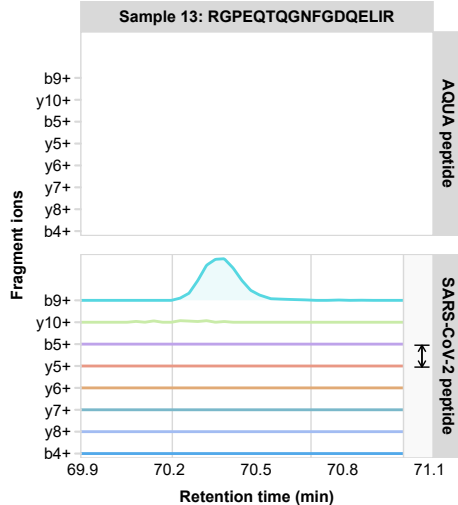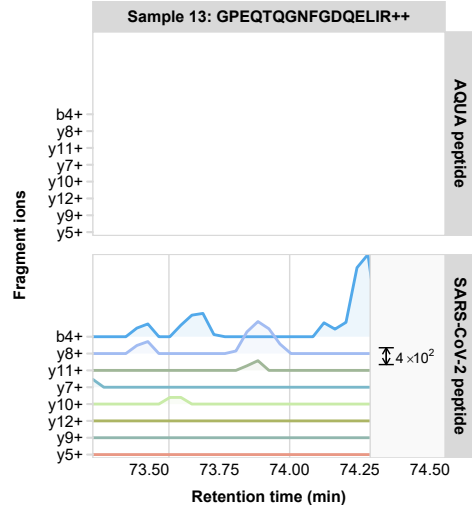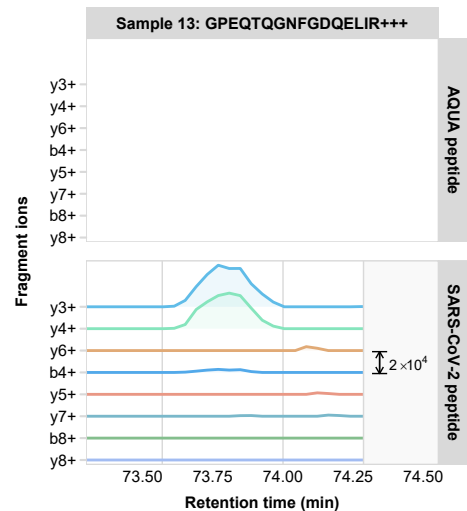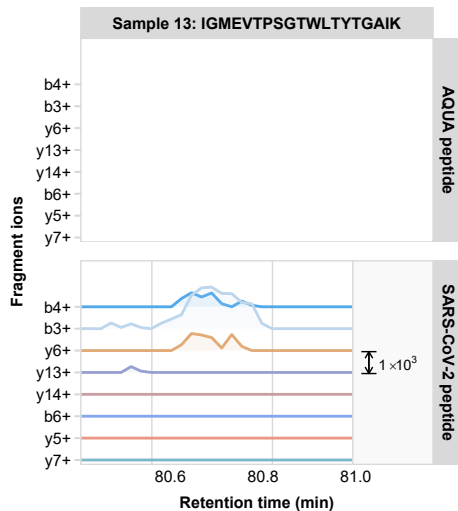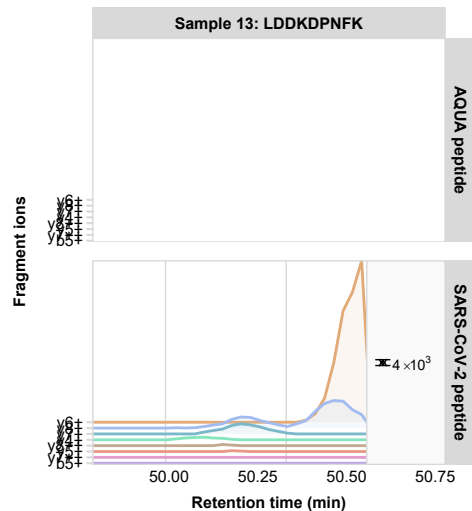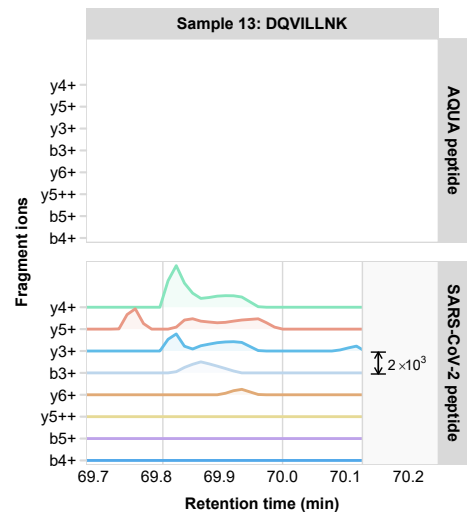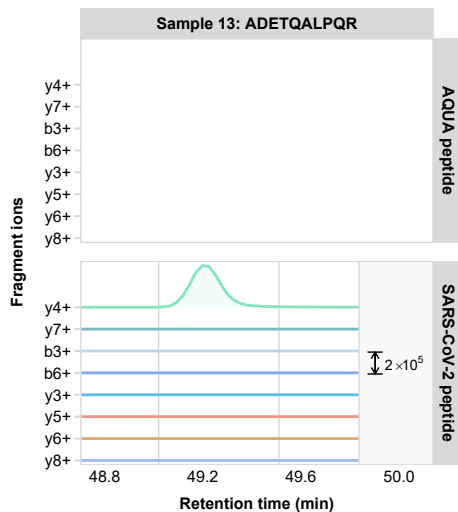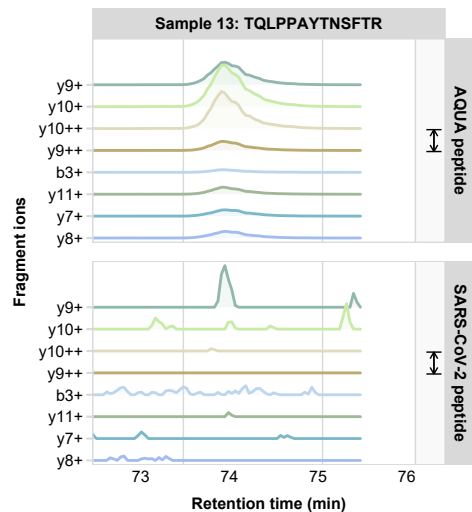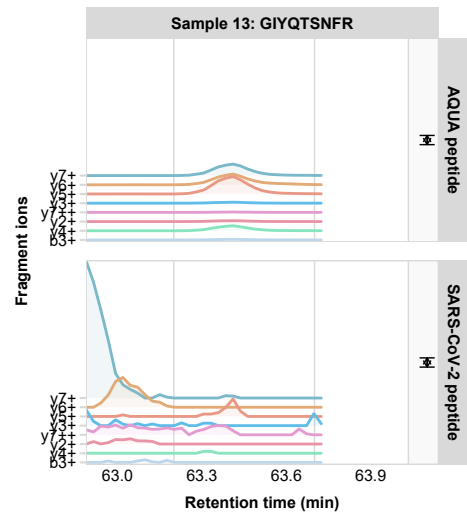

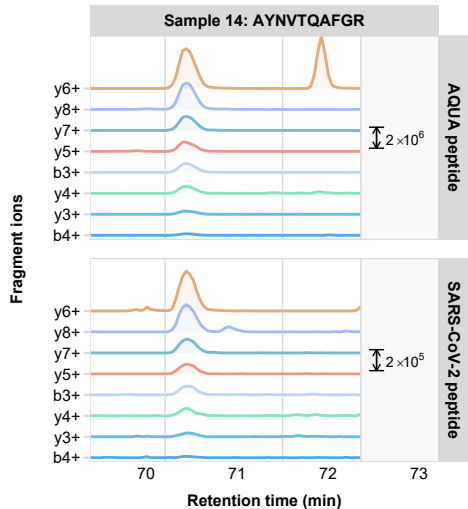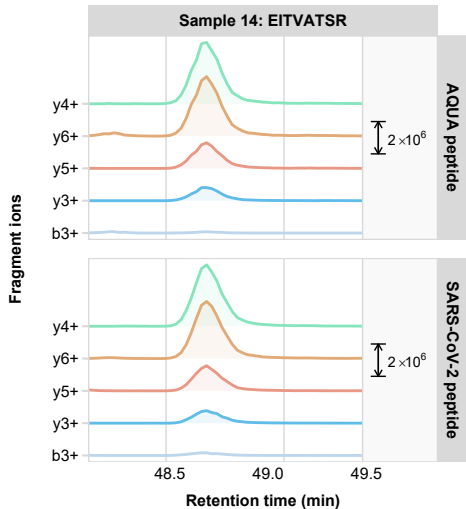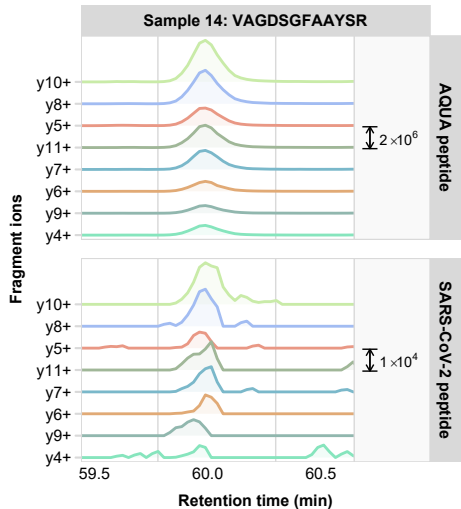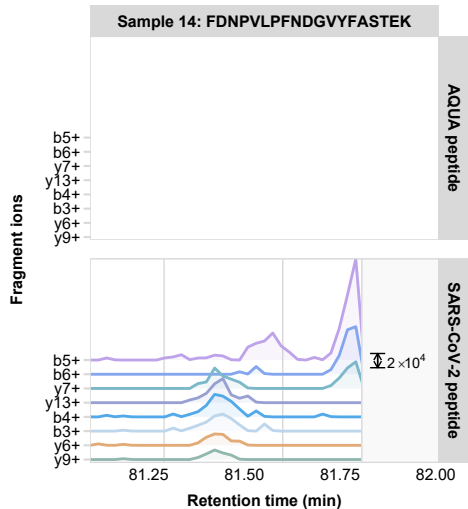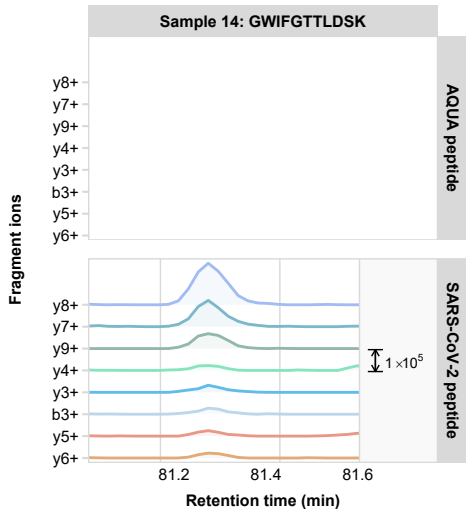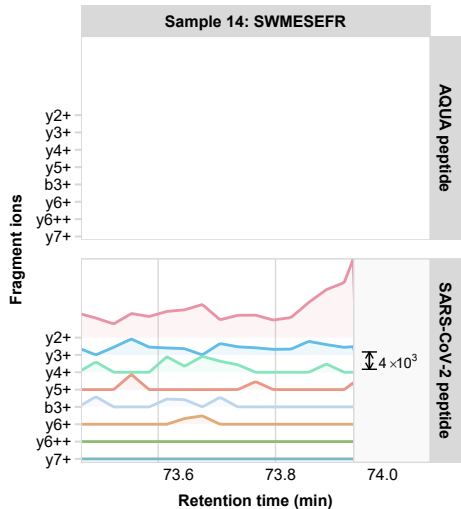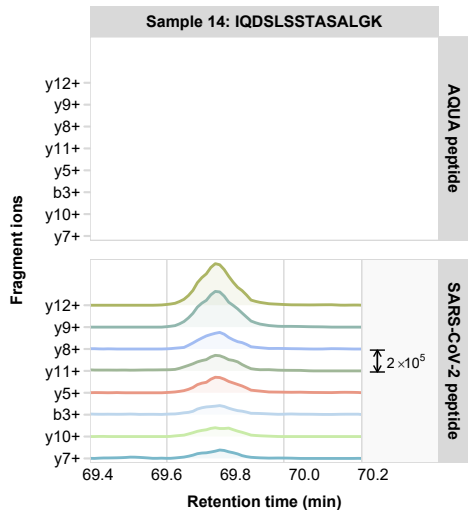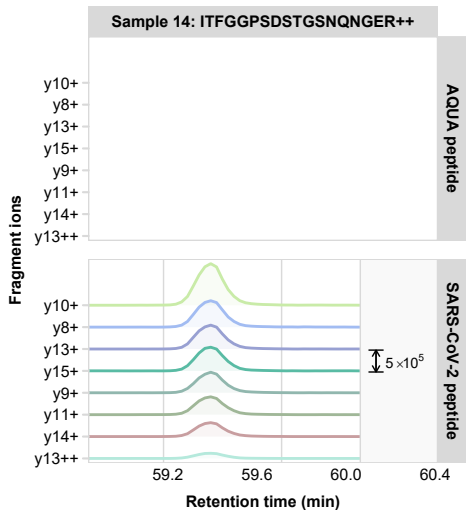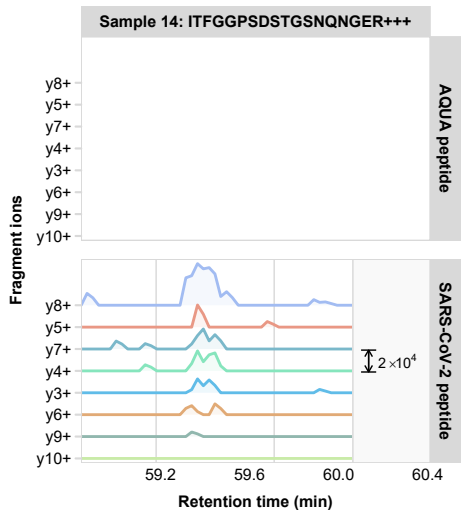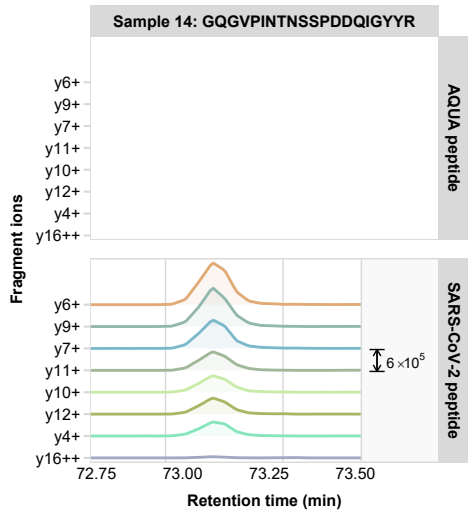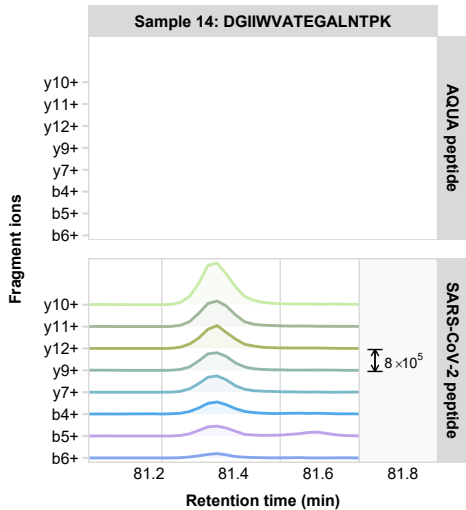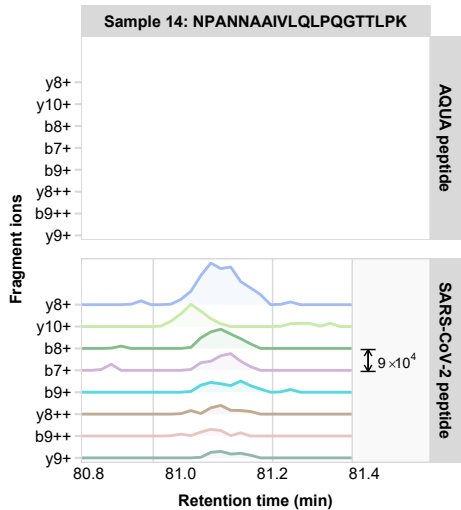

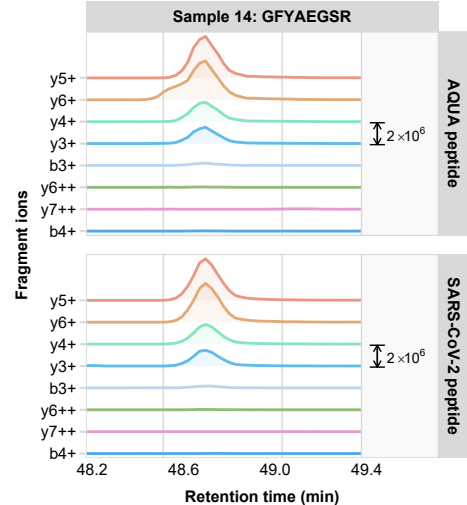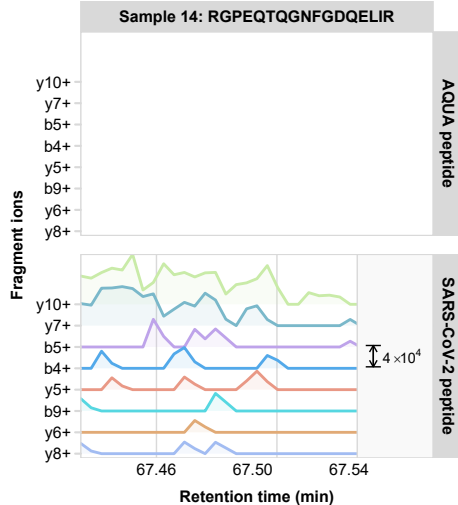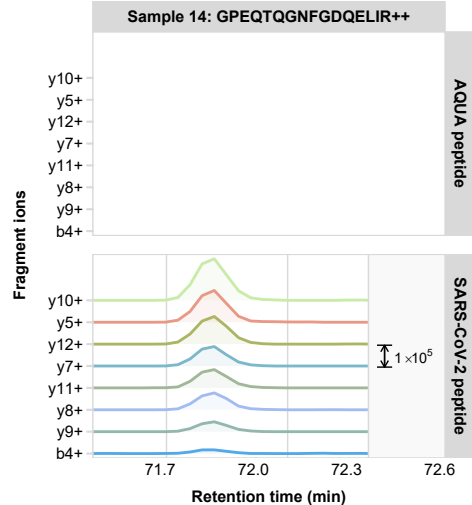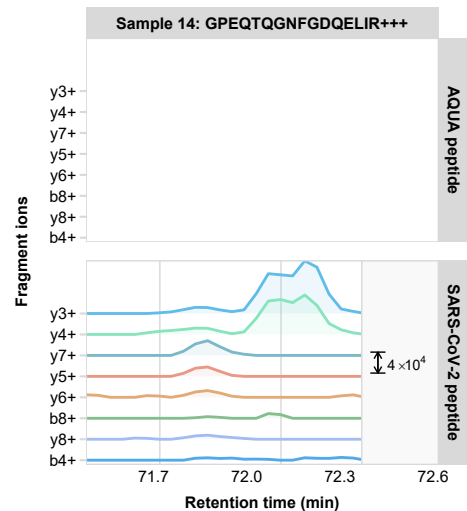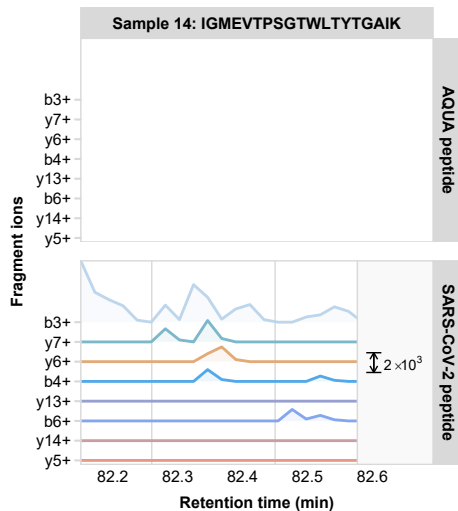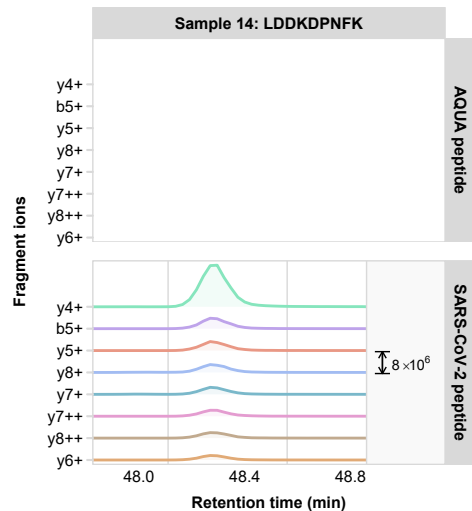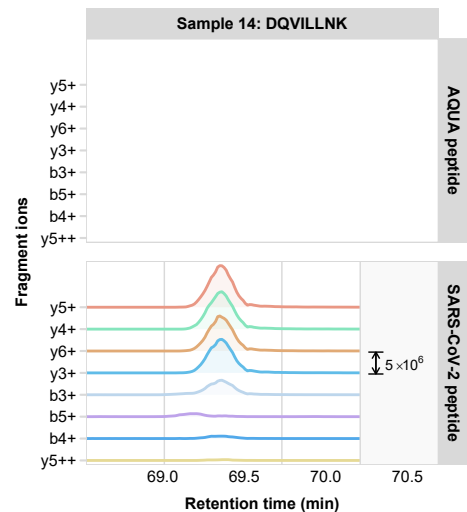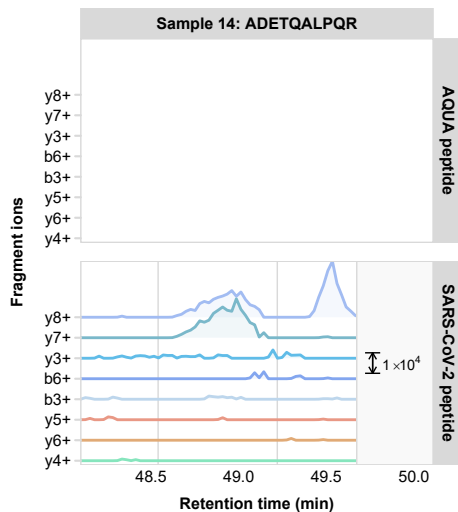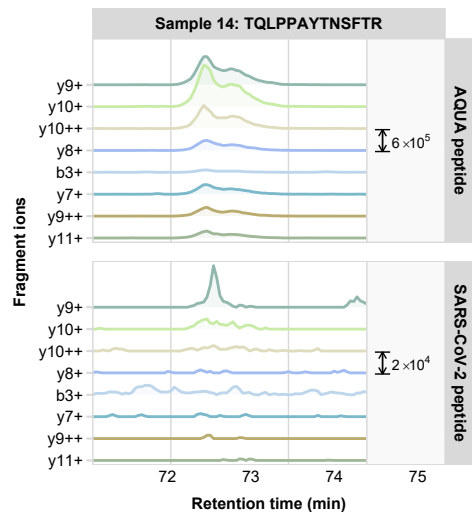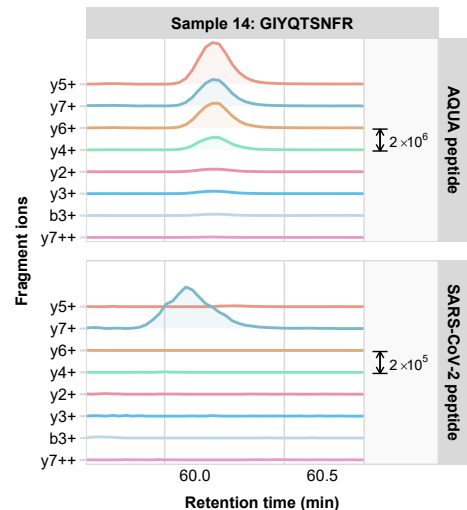

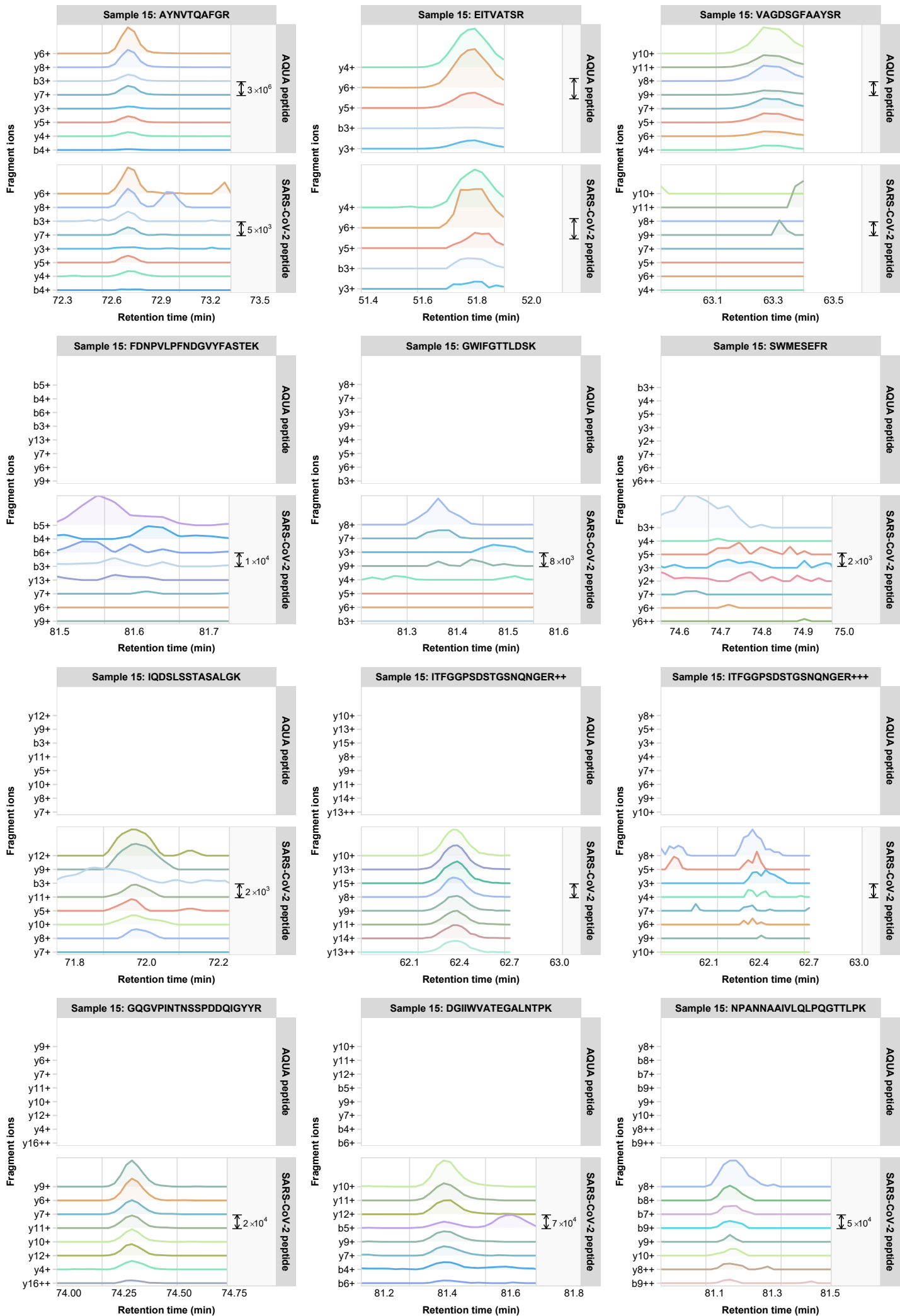

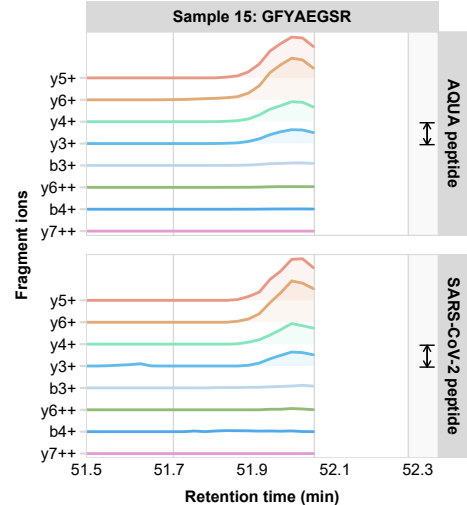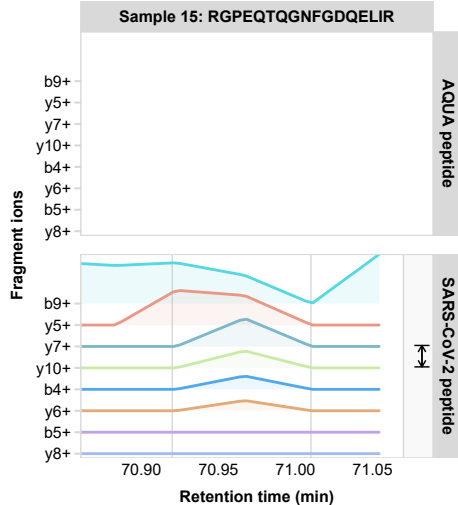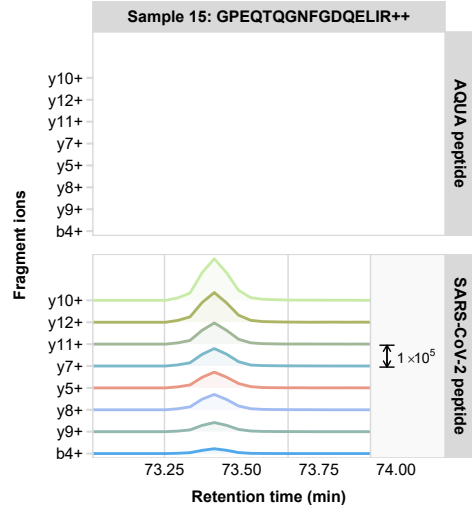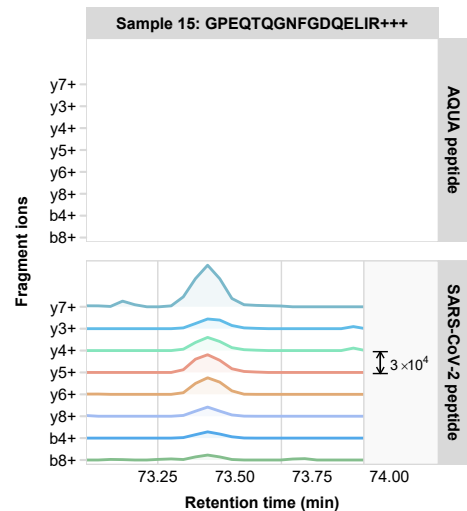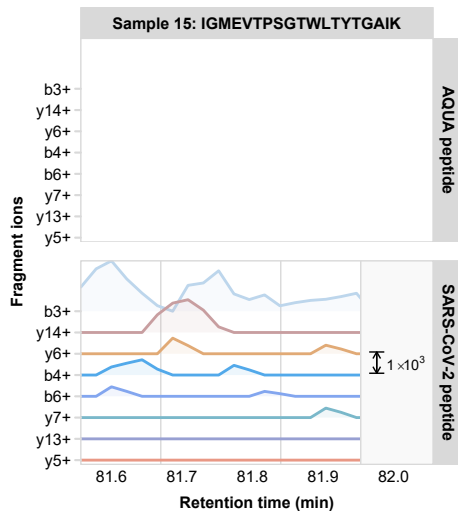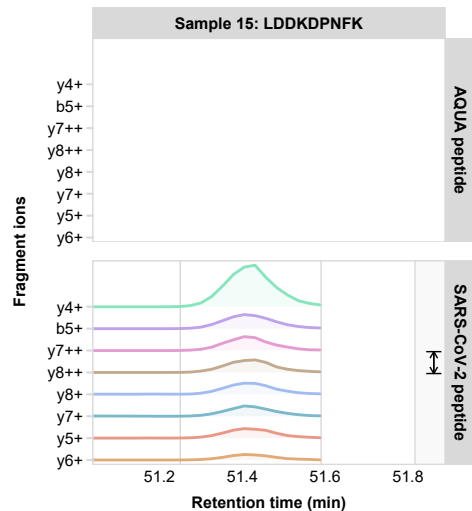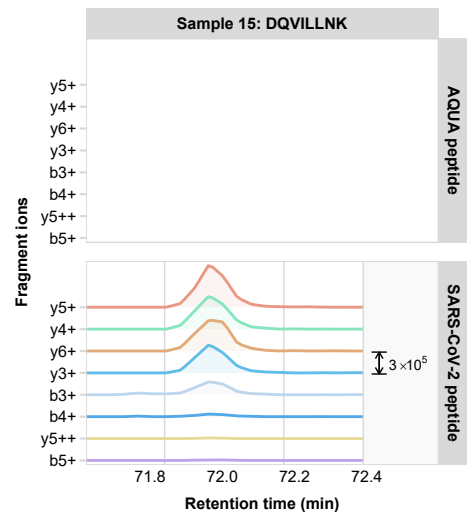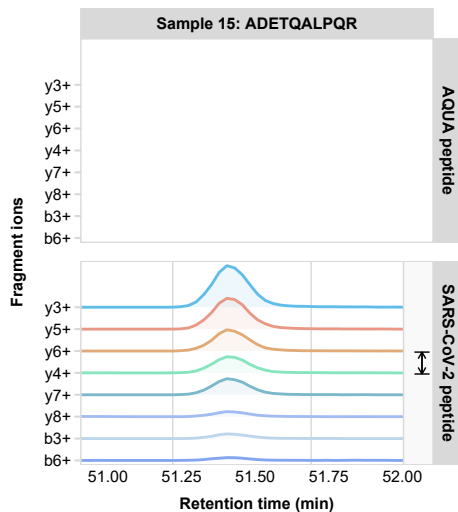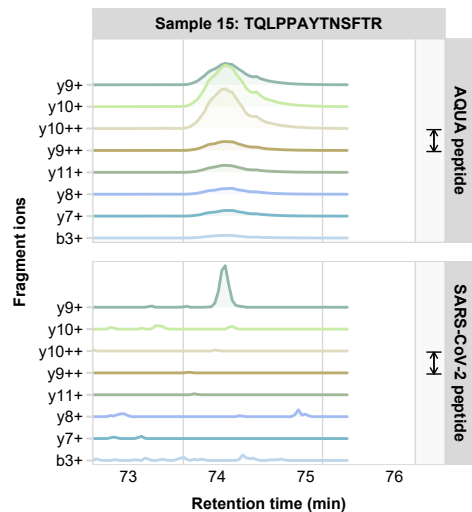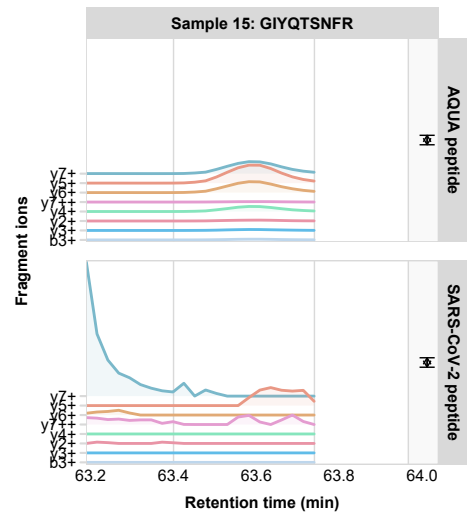

Supplement: S8 File — (PDF) [file pone.0259165.s014.pdf]
