# Supplementary material for: Targeted proteomics as a tool to detect SARS-CoV-2 proteins in clinical specimens
Source: PLoS One. 2021 Nov 11;16(11):e0259165. doi: 10.1371/journal.pone.0259165 (PMC8584957; doi:10.1371/journal.pone.0259165)
Supplement: Attachment — Submitted filename: Response_to_Reviewers.docx [file pone.0259165.s016.docx]

**Reply to editorial comments with respect to journal requirements (in bold blue font)**

1. Please ensure that your manuscript meets PLOS ONE's style requirements, including those for file naming. The PLOS ONE style templates can be found at

<https://journals.plos.org/plosone/s/file?id=wjVg/PLOSOne_formatting_sample_main_body.pdf> and

[https://journals.plos.org/plosone/s/file?id=ba62/PLOSOne_formatting_sample_title_authors_affiliations.pdf](about:blank)

**We have formatted the main text, title, author affiliations, file naming, etc. according to the *PLOS ONE* style requirements.**

2. Please provide additional details regarding participant consent. In the ethics statement in the Methods and online submission information, please ensure that you have specified (1) whether consent was informed and (2) what type you obtained (for instance, written or verbal, and if verbal, how it was documented and witnessed). If the need for consent was waived by the ethics committee, please include this information.

**The Jeroen Bosch Hospital and the Star-shl Medical Diagnostic Center, the institutes that have provided us with clinical samples, have obtained the proper informed consent from all tested individuals. We have included an ethics statement in the manuscript, which reads:**

***Ethics statement
The Institutional Review Boards of both the Jeroen Bosch Hospital and Star-shl Medical Diagnostic Center approved this study. The boards approve anonymous use of remnant biospecimens for scientific purposes. All patients were informed of the possibility that residual samples could be used anonymously for research purposes with right of refusal. All samples were anonymized before they arrived at the Erasmus MC Proteomics Center for further analysis.***

3. Please include your tables as part of your main manuscript and remove the individual files. Please note that supplementary tables (should remain/ be uploaded) as separate "supporting information" files.

**We have now included table 1 in the main text and removed the separate table. Supplementary tables have been renamed to ‘Supporting Information’ and uploaded along with the manuscript.**

4.  Thank you for stating the following in the Financial Disclosure section: "The author(s) received no specific funding for this work."

**The following statement has been included in the manuscript:**

***Financial disclosure
The authors received no specific funding for this work and declare no competing interests. Star-shl Medical Diagnostic Center is a non-profit organization, which provides routine microbiology diagnostic services for primary health care facilities of general practitioners (GPs), nursing homes and STI-clinic of the public health services.***

We note that one or more of the authors are employed by a commercial company: "Star-shl Diagnostic Laboratories,"

a) Please provide an amended Funding Statement declaring this commercial affiliation, as well as a statement regarding the Role of Funders in your study. If the funding organization did not play a role in the study design, data collection and analysis, decision to publish, or preparation of the manuscript and only provided financial support in the form of authors' salaries and/or research materials, please review your statements relating to the author contributions, and ensure you have specifically and accurately indicated the role(s) that these authors had in your study. You can update author roles in the Author Contributions section of the online submission form.

Please also include the following statement within your amended Funding Statement.

“The funder provided support in the form of salaries for authors [insert relevant initials], but did not have any additional role in the study design, data collection and analysis, decision to publish, or preparation of the manuscript. The specific roles of these authors are articulated in the ‘author contributions’ section.”

If your commercial affiliation did play a role in your study, please state and explain this role within your updated Funding Statement.

b) Please also provide an updated Competing Interests Statement declaring this commercial affiliation along with any other relevant declarations relating to employment, consultancy, patents, products in development, or marketed products, etc.

Within your Competing Interests Statement, please confirm that this commercial affiliation does not alter your adherence to all PLOS ONE policies on sharing data and materials by including the following statement: "This does not alter our adherence to PLOS ONE policies on sharing data and materials.” (as detailed online in our guide for authors [http://journals.plos.org/plosone/s/competing-interests](about:blank)). If this adherence statement is not accurate and  there are restrictions on sharing of data and/or materials, please state these. Please note that we cannot proceed with consideration of your article until this information has been declared.

Please include both an updated Funding Statement and Competing Interests Statement in your cover letter. We will change the online submission form on your behalf.

**We would like to stress that Star-shl Medical Diagnostic Center is a *non-profit* organization, which provides laboratory services in the South-West part of the Netherlands. Routine microbiology diagnostics (*e.g.*, serology, molecular testing, bacterial cultures) are performed for primary health care facilities of general practitioners, nursing homes and STI-clinics of the public health services. Please see** [**https://www.rva.nl/en/accredited-organisations/details/539**](https://www.rva.nl/en/accredited-organisations/details/539) **for further information about this organization. Therefore, we take the liberty to waive the above described requirements to include statements concerning the involvement of a commercial party. Obviously, the adherence to *PLOS ONE* policies on sharing data and materials is true for this foundation just like for all other, academic affiliations listed on the title page.**

**Reply to Reviewer 1’s comments (in bold blue font)**

**We thank the Reviewer for the very positive comments regarding the relevance of the manuscript and the novelty of the methodology in diagnostics and clinical care. We will reply to the Reviewer’s comments in detail below.**

1. Is the manuscript technically sound, and do the data support the conclusions?

Reviewer #1: Yes

**No comments.**

2. Has the statistical analysis been performed appropriately and rigorously?

Reviewer #1: Yes

**No comments.**

3. Have the authors made all data underlying the findings in their manuscript fully available?

Reviewer #1: Yes

**No comments.**

4. Is the manuscript presented in an intelligible fashion and written in standard English?

PLOS ONE does not copyedit accepted manuscripts, so the language in submitted articles must be clear, correct, and unambiguous. Any typographical or grammatical errors should be corrected at revision, so please note any specific errors here.

Reviewer #1: Yes

**No comments.**

5. Review Comments to the Author

Reviewer #1: The manuscript, “Targeted proteomics as a tool to detect SARS-CoV-2 proteins in clinical specimens” by Bezstarosti et al, is an interesting and informative article. The paper addresses a timely question regarding the COVID-19 pandemic which has devastated the whole world. In this paper, the authors have done comprehensive research to establish a PRM MS-based method that can be utilized for COVID-19 diagnosis. Authors have cultured Vero E6 cells and infected them with SARs-CoV-2 to optimize the MS-based method which they have eventually extended to detect SARs-CoV-2 peptides in COVID-19 positive patient specimens. The authors were able to detect various SARS-CoV-2 tryptic peptides in nasopharyngeal swab samples of COVID-19 positive patients and have also investigated the relationship between amounts of viral RNA and protein abundances.

**We thank the Reviewer for the very positive comments. Below, we will reply to the comments and questions raised by this Reviewer.**

1) The study includes human sputum and swab samples. Did the authors get consent from the patients? Was this study been approved by any ethical committee? Please share the ethical committee documents and ICF used for the study. Please mention in the sample collection details of the methodology.

**Erasmus MC Rotterdam has declared that the study described in this manuscript is not subject to the Medical Research Involving Human Subjects Act (in Dutch: ‘wet medisch-wetenschappelijk onderzoek met mensen’ or WMO – see the uploaded statement (in Dutch) of the Medical Ethics Review Committee regarding this study). Clinical samples used in this study were all obtained by other institutes and considered waste material after initial use. Instead of being discarded according to routine procedures, they were kindly provided to us. In the uploaded Data and Material Transfer Agreement the section on ‘Informed Consent’ (6.2) reads:**

***“[…] provider represents that to the extent applicable it has obtained the proper Informed Consent from each Subject in accordance with its Applicable Law, which Informed Consent allows for the transfer of the Data and/or Material for the Purpose […]”***

**The Jeroen Bosch Hospital and the Star-shl Medical Diagnostic Center, the institutes that have provided us with clinical samples, have obtained the proper informed consent from all tested individuals. We have included an ethics statement in the manuscript, which reads:**

***Ethics statement
The Institutional Review Boards of both the Jeroen Bosch Hospital and Star-shl Medical Diagnostic Center approved this study. The boards approve anonymous use of remnant biospecimens for scientific purposes. All patients were informed of the possibility that residual samples could be used anonymously for research purposes with right of refusal. All samples were anonymized before they arrived at the Erasmus MC Proteomics Center for further analysis.***

2) Authors have selected three peptides while working with Vero E6 cells infected with SARS-CoV-2 for PRM based targeting to diagnose COVID-19. Were there any specific criteria used to select these peptides? What other peptides were tested which have the potential to be used for diagnostic purposes.

**In principle, all identified peptides for any of the SARS-CoV-2 proteins listed in Supplementary Table 1 could be used as targets for diagnostic purposes. However, since some tryptic peptides are more mass spectrometrically responsive than others, we have included the peptides with highest response in order to obtain highest assay sensitivity. The three peptides highlighted in Figure 2 GFYAEGSR (Nucleocapsid), ADETQALPQR (Nucleocapsid) and EITVATSR (Membrane protein) are among the most responsive peptides. Also, this selection contains peptides unique to SARS-CoV-2 compared to *e.g.* SARS-CoV or MERS. It is important to stress here that there are many other peptide candidates among these highly responsive peptides and that the peptides displayed in Figure 2 are just shown as examples of these. Another prominent tryptic peptide that we used for PRM experiments of clinical samples is AYNVTQAFGR (Nucleocapsid), which is also unique to SARS-CoV-2.**

3) In human sputum and swab samples, the authors performed PRM analysis on tryptic peptide AYNVTQAFGR and VAGDSGFAAYSR. Were these peptides analyzed in the first PRM experiment also where authors used three peptides from SARS-CoV-2 infected Vero E6 cells?

**Both peptides were certainly also identified in the first PRM experiments. In fact, AYNVTQAFGR and VAGDSGFAAYSR were also among the highest responsive peptides in the first PRM experiment and could have been added to Figure 2 if space would have allowed. For most highly responsive peptides we also have the synthesized AQUA counterparts.**

4) In the main text, while explaining figure 3 it is not clear what is panel C. There is an upper panel that shows AQUA peptide, the lower panel shows the corresponding endogenous peptide. Please clarify the text in the manuscript. The figure legend is explained correctly though.

**We thank the Reviewer for pointing out that the main text was not correct. We have updated the main text so that Figure 3 is explained correctly now.**

5) While generating the calibration curve, peptide VAGDSGFAAYSR has been quantified in both sputum and swab samples. However, in nasopharyngeal swabs of cohort 1, the peptide has not been identified in any of the samples as depicted in supplementary information 1. Please explain.

**The reviewer is correct in that peptide VAGDSGFAAYSR was identified in the sputum and swab samples. In Cohort 1 the VAGDSGFAAYSR panel was left out, because the peptide was not detected in any of the samples. Although a fragment ion chromatogram pattern similar to the corresponding AQUA peptide was indeed observed in sample 5, the quality filters that we set did not allow inclusion of that peptide in the final overview.**

**Generally, the clinical specimens that were kindly provided to us by others suffered from heterogeneity and non-optimal storage conditions. Different sample cohorts were obtained from different COVID-19 testing sites and thus collected by different health care workers. It is impossible to compare the sample preparation and especially the storage conditions between samples from different cohorts. For instance, proteins may have been partially degraded while specimens were left at room temperature for too long. Anyhow, these different conditions will inevitably lead to variability in the tryptic digestion process. We suspect that this may lead to some tryptic peptides of SARS-CoV-2 proteins being identified in one cohort, while a partially different set of peptides were identified in the other. Because of the shortage of proper clinical samples it was not possible to extensively vary sample preparation procedures to optimize the LC-MS analysis.**

**Although we believe that uniform sample preparation is key in proteomics experiments, the analyses described in this manuscript suffered from sample heterogeneity, possibly leading to slightly different tryptic peptide coverages for target proteins.**

6) The authors propose that higher sensitivity can be achieved by fractionating the samples. Since time is the limiting factor when one works with fractionated samples, what could be the least number of fractions that can be considered to improve the sensitivity of PRM based diagnosis.

**The Reviewer is correct in that a balance should be sought between analysis time and detection sensitivity. In this assay we have fractionated peptides into eight fractions by means of high pH reversed phase (RP) chromatography. The purpose of this fractionation assay was to show that the detection sensitivity can be increased upon sample fractionation. This experiment was merely meant as a proof-of-concept. Whether or not it is feasible to include extensive fractionation procedures depends on several other parameters, such as available time and budget. However, if crude fractionation into two or three fractions would help to increase the sensitivity by ~2-fold, it could be worthwhile to include this in the standard protocol.**

7) Authors have found correlation between PCR Ct values and summed mass spectral intensities of peptides from PRM analysis. The current standard for Covid-19 positivity is Ct value of less than 35. Is there any absolute value of peptide quantification that can be used as a threshold for positivity of infection or is it just identification of the peptides which can confirm the presence of SARs-CoV-2 virus?

**We have not performed any experiments to relate PRM sensitivity to infectiousness. While we have made comparisons between PCR Ct values and summed fragment ion chromatogram AUCs and found a reasonable correlation between them, viral infectiousness should be tested with other methods. Using the methodology described here we can relatively quantify peptide/protein abundances, which may vary by several orders of magnitude between the different samples tested. Obviously, we suspect that the more viral protein is present, the higher chances are that intact, infectious virus particles are present in such samples. However, the tryptic peptides that we measure could as well originate from digestions of non-infectious protein assemblies, non-assembled proteins or ‘old’ viral material that is still present in recently recovered COVID-19 patients.**

**Strikingly, for some high Ct value samples we could still detect SARS-CoV-2 tryptic peptides, while for other samples with lower Ct values (*i.e.*, more viral RNA present) we could not. Also, for samples having similar Ct values but originating from different testing sites, the summed fragment ion chromatogram AUCs in our assays were sometimes significantly different. We have no explanation for this yet and this is subject of current studies. Finally, we have noticed that the cutoff Ct value of 35 is subject to debate (*e.g.* 1) Bullard *et al. Predicting Infectious Severe Acute Respiratory Syndrome Coronavirus 2 From Diagnostic Samples*. Clinical Infectious Diseases 2020 and 2) Singanayagam *et al. Duration of infectiousness and correlation with RT-PCR cycle threshold values in cases of COVID-19, England, January to May 2020*. Euro Surveill. 2020).**

**In summary, additional studies should be performed to be able to relate mass spectral intensities to infection positivity.**

8) PRM analysis has been carried out using two cohorts of nasopharyngeal swab samples from Covid-19 positive patients in the current study. To make the PRM based diagnostic method feasible both in terms of analysis and time, authors should summarize the list of peptides that can serve as potential candidates to detect the presence of SARS-CoV-2 virus in nasopharyngeal swabs.

**We provide the *m/z* inclusion list for PRM studies as supporting information (Supporting_Information_2_Isolation_list_PRM_Eclipse) with the manuscript, the numbers in which can be used straight away by researchers who would wish to copy this methodology in their own labs. For the reviewer’s and the readers’ convenience, we have now added the peptide sequences to this list as well.**

**Reply to Reviewer 2’s comments (in bold blue font)**

**We thank the Reviewer for their very positive comment regarding the relevance of the manuscript and the novelty of the methodology in diagnostics and clinical care. We will reply to the Reviewer’s comments in detail below.**

1. Recommendation - Major Revision

**We have revised the manuscript and figures to the best of our abilities according to the Reviewer’s comments and suggestions.**

2. Is the manuscript technically sound, and do the data support the conclusions? - Partly (Proteomic and data analysis section requires major revisions)

**We have revised the proteomics and data analysis sections according to the Reviewer’s comments and suggestions. See below for a detailed description of the changes.**

3. Has the statistical analysis been performed appropriately and rigorously? - No, The authors should explain the statistical methods employed

**We have revised the description of the statistical methods applied according to the Reviewer’s comments and suggestions. See below for a detailed description of the changes.**

4. Does the manuscript adhere to the PLOS Data Policy? Additional details can be found at – Yes

**No comments.**

5. Is the manuscript presented in an intelligible fashion and written in standard English? – Yes

**No comments.**

6. Review Comments to the Author - Please see appended below:

The manuscript titled “Targeted proteomics as a tool to detect SARS-CoV-2 proteins in clinical specimens” is really a great initiative in showing the advantage of LC-MS-based diagnostic in clinical care, especially in our battle against the COVID-19 pandemic. The authors have claimed to have found good sensitivity for several SARS-CoV-2 proteins from their targeted LC-MS method (PRM). Nonetheless, there are several sections of the manuscript that require major changes prior to the publications.

**We thank the Reviewer for their very positive comment regarding the relevance of the manuscript and the novelty of the methodology in diagnostics and clinical care.**

-The authors did not mention the parameters used for Skyline analysis such as regression weightage, normalization method, LOD calculation method, among many other such parameters, which are crucial in case of absolute quantification. I request the authors to incorporate such information in the manuscript.

**We apologize for the omission of the Skyline settings in the manuscript. We have now added the Skyline settings for the generation of the calibration curves in the manuscript main text as well as in a table as supporting information as requested (regression fit: bilinear through zero; regression weighting: none; normalization method: none; LOD calculation method: bilinear turning point: mac LOQ bias: [*left empty*]; max LOQ CV: [*left empty*]; qualitative ion ratio threshold: [*left empty*]). The calculated LOD for *e.g.* target peptide** **GFYAEGSR is then 2.0E-11 g. Please note that for the input on the x-axis we use the amount of total protein digest from SARS-CoV-2 infected Vero E6 cells, while no AQUA peptides were used for absolute quantitation as explained in detail below. As a consequence, we cannot know the precise amount of the specific SARS-CoV-2 target tryptic peptides. This is clarified in the manuscript text.**

**We have adapted Figure 2C as well as the main text to clarify this point. Furthermore, in order to shift the focus of the manuscript to the clinical specimen data and to make it more concise, we have moved Figure 2C to Supplementary Figure 3. We feel that the reader is more appropriately exposed to the main message of the study now.**

-Based on the method sections, we can assume that the authors have used SARS-CoV-2 infected Vero E6 cell lysates to develop and generate a standard calibration curve. However, it is not clear whether the AUQA heavy labelled peptides were spiked to the same and if so, at what concentrations?

**For the calibration curves we use the amount of total protein digest from SARS-CoV-2 infected Vero E6 cells as the input, since we do not have access to isolated SARS-CoV-2 particles or proteins. No AQUA peptides were used for absolute quantitation. All identified and quantified SARS-CoV-2 proteins were present in a Vero E6 cell background as it is not possible to grow and collect the virus without the presence of these infected host cells. As a result, we do not know the exact input amounts of SARS-CoV-2 proteins that were used for the generating the calibration curve. The indicated total amount of protein on the x-axis of the calibration curves represents the total amount of digested proteome of SARS-CoV-2 infected Vero E6 cells. It is not possible, other than with *e.g.* LFQ mass spectrometry based methods – as described in this study -, to calculate the ratio of SARS-CoV-2 proteins versus Vero E6 host cell proteins. In fact, we have chosen for this experimental setup deliberately, since in all research or patient samples containing SARS-CoV-2 the levels of viral proteins were expected to be relatively low as compared to contaminating background proteomes.**

**We did not use AQUA peptides for the generation of the calibration curves for the following reasons:**

- **One practical reason is that at the time of generating the calibration curves we did not possess the AQUA peptides yet, since as a result of the Covid-19 crisis mid-last year the delivery of such (customized) consumables was largely delayed.**
- **The exact amount of commercially synthesized AQUA peptides that we obtained is not exactly known and so the final concentration of stock solutions could only be estimated to be in a range spanning at least a half order of magnitude.**
- **Although potentially relevant in other types of studies, we did not focus on the absolute quantitation of SARS-CoV-2 tryptic peptides/proteins here. For both the infected Vero E6 cell line and the clinical specimens, the contaminating background proteomes (the Vero E6 cellular proteome or the human saliva / mucus / epithelial cellular / etc. proteomes, respectively) and especially the heterogeneous character thereof suppress the SARS-CoV-2 target tryptic peptides substantially and – importantly - to variable extents. Therefore, in our opinion it would not make sense to focus on absolute quantitation in this case. From previous studies in our lab we know that limits of quantitation (LOQs) largely depend on the presence or absence of contaminating background proteomes. Especially in the clinical specimens, the contaminating background levels were extremely variable.**

**In order to avoid any confusion and to shift the focus of the manuscript from the calibration curves to the clinical specimen data, we have moved Figure 2C to Supplementary Figure 3. We feel that it is more concise now and that the reader is more appropriately exposed to the main message of the study.**

**The central message of the manuscript is the ability to detect SARS-CoV-2 proteins in clinical specimens and the comparison of mass spectral intensities of viral proteins to PCR Ct values of those same clinical specimens, not so much the absolute quantitation of SARS-CoV-2 proteins. The main reason to use heavy labeled AQUA peptides was for confirmative purposes: As we expected the MS intensity signals of SARS-CoV-2 tryptic peptides in patient samples to be very low and present in a complex matrix background, we spiked in AQUA peptides to be absolutely sure that the correct endogenous peptides were targeted and identified. Only by comparing the relative fragment ion intensities and retention times of both endogenous and spiked in AQUA peptides, the high quality and reliability of these analyses could be ensured.**

**AQUA peptides were commercially synthesized and, depending on the exact price/quality package chosen, the absolute amounts can be given only in ballpark figures. It is therefore not possible for us to know the exact amount of the specific AQUA peptides that we purchased and the exact concentration of the spiked in peptides other than in a ballpark concentration range.**

**Indeed, we show in Figure 6A that the expected concentration of endogenous peptide that was detected in fractionated sample #7 is in the ‘low to mid-attomole range’. We have deliberately not specified this concentration range in more detail, since this estimation is based on the abovementioned purchased AQUA peptide batch. Consequently, this concentration should be considered as merely as an educated guess or rough estimation. We have adapted the manuscript text to state that the indicated numbers should only be considered as an educated guess.**

-How did the authors handle the presence of endogenous viral tryptic peptides (non-labeled) while generating the calibration curve?

**We wish to point out that we used only endogenous tryptic viral peptides (present in a complex matrix background) for the generation of the calibration curves, no AQUA internal standards were used to generate them for the reasons listed above.**

-Why an Area Under Curve (AUC) was used for the generation of the calibration curve? In general, it is the area ratio of the non-labeled:labeled was used to generate the calibration curve and quantification. Why did the authors not used internal standards to do as it is the highly recommended approach in targeted mass-spectrometry based absolute quantification analysis?

**We wish to point out that we used only endogenous tryptic viral peptides for the calibration curves, no AQUA internal standards were used to generate them for the reasons listed above. We apologize for the confusion and have adapted the manuscript text to clarify this issue.**

**To our knowledge, the determination of the area under the curve (AUC) is an established method for target peptide quantitation. In fact, the use of calculating the background subtracted area under the curve of the precursor or fragment ion chromatograms over the peptide elution as a quantitative metric for each peptide in Skyline is described in e.g. Egertson JD, MacLean B, Johnson R, Xuan Y, MacCoss MJ. Multiplexed peptide analysis using data-independent acquisition and Skyline. Nature Protocols 2015;10(6):887-903. doi:10.1038/nprot.2015.055. In Skyline, the peak area, or AUC, refers to the total integrated area within the peak boundaries, minus the background area. Background area is defined as the total integrated area of the minimum of background height and intensity at each point, where background height is the minimum intensity of the two points where the chromatogram crosses the integration boundaries, which is assumed to be the level of intensity contributed not by the transitions themselves but from chemical noise (background) in the measurement. The background area is subtracted from the total integrated area within the peak boundaries to return the final reported peak area. Although Skyline allows display of chromatograms with various smoothing options (2D, 1D, Savitzky-Golay) applied, it uses the interpolated points displayed in the unsmoothed graphs to calculate peak area. Total area values sum the AUC values of individual chromatograms, rather than performing a separate AUC calculation on a summed chromatogram. As such, the AUCs can thus also be used for quantitation and for the generation of calibration curves.**

-How the authors have selected fragment ions for the quantification? Based on Skyline document shared in PRIDE repo it is clear that both b and y ions were selected for the analysis. However, it is the 'y' ions considered the more reliable over 'b' ions as these b ions are not stable post fragmentation.

**The selection of fragment ions was based on relative intensities: the (up to ten) highest intense fragment ions were selected, all based on previously recorded spectra using the identical LC-MS setup and the same mass spectrometer, rather than on *in silico* predicted spectra. Since all peptide fragmentation experiments were performed under identical conditions (in terms of collision energy, mass spectrometer, etc.) we do not expect the suggested instability of b ions to have an effect on peptide quantitation in this case. Also, we observe the exact same fragment ion intensity patterns for the endogenous and AQUA peptides (except for the y ions being 10 Da higher in mass because of the isotope labeled C-terminal Arginine residues). We do not observe any differences between the relative shapes or heights of the b and y fragment ion chromatograms at extremely low and extremely high input concentrations of peptides.**

**In summary, these observations confirm that the selections of fragment ions, including b ions, were chosen correctly and adequately.**

-While performing the acquisition of both calibration curve and patient samples, are there any QC samples acquired in between? If not, why the authors have not used QC samples as the acquisition of QC is crucial while performing the absolute quantification experiments to maintain any human or instrument errors.

**QC measurements are certainly performed on a regular basis. In between all patient samples, both blank runs and QC runs are performed to ensure optimal performance of the LC-MS system. QC controls are usually tryptic digests of HeLa cells, of purified albumin and mixtures of standard synthesized iRT peptides, which should always meet the minimum standards that we set. If they do not, the LC-MS system is thoroughly cleaned, recalibrated and settings are optimized until the requirements are met. This procedure is common practice in many proteomics labs. Also, calibration curves are obviously performed starting with the blank and lowest concentration first and then moving to higher concentrations.**

-The authors are requested to write the PRM and DDA parameters in detail either in the method section or as supplementary.

**We have added supplementary tables containing all essential parameters for the DDA and PRM mass spectrometry analyses, as well as the Skyline settings used. In addition, we have adapted the manuscript text, including the Materials and Methods section as requested by the Reviewer. The text now contains all necessary details about the DDA and PRM parameters, so that other researchers can copy the described methods in their own labs.**
